# Soyauxinine, a New Indolopyridoquinazoline Alkaloid from the Stem Bark of *Araliopsis soyauxii* Engl. (Rutaceae)

**DOI:** 10.3390/molecules27031104

**Published:** 2022-02-07

**Authors:** Cédric Guy Tchatchouang Noulala, Judith Laure Nantchouang Ouete, Albert Fouda Atangana, Gabin Thierry Bitchagno Mbahbou, Ghislain Wabo Fotso, Hans-Georg Stammler, Bruno Ndjakou Lenta, Emmanuel Ngeufa Happi, Norbert Sewald, Bonaventure Tchaleu Ngadjui

**Affiliations:** 1 Department of Organic Chemistry, Faculty of Science, University of Yaoundé 1, Yaoundé P.O. Box 812, Cameroon; cedric.noulala@hotmail.fr (C.G.T.N.); ouetejudit@yahoo.fr (J.L.N.O.); ngadjuibt@yahoo.fr (B.T.N.); 2 Organic and Bioorganic Chemistry, Department of Chemistry, Bielefeld University, 33501 Bielefeld, Germany; bmgt198716@gmail.com; 3 Department of Chemistry, Faculty of Science, University of Maroua, Maroua P.O. Box 55, Cameroon; atangana_albert@yahoo.fr; 4 Department of Chemistry, Faculty of Science, University of Dschang, Dschang P.O. Box 67, Cameroon; 5 Department of Chemistry, Inorganic and Structural Chemistry, Bielefeld University, 33501 Bielefeld, Germany; georg.stammler@uni-bielefeld.de; 6 Department of Chemistry, Higher Teacher Training College, University of Yaoundé 1 1, Yaoundé P.O. Box 47, Cameroon; lentabruno@yahoo.fr; 7 Department of Chemistry, Faculty of Science, University of Douala, Douala P.O. Box 24157, Cameroon

**Keywords:** *Araliopsis soyauxii*, Rutaceae, alkaloids, soyauxinine, cytotoxic activity

## Abstract

The chemical investigation of the total alkaloid extract (TAE) of the stem bark of *Araliopsis soyauxii* (Rutaceae) afforded an unreported indolopyridoquinazoline (compound **1**) along with nine previously known alkaloids **2**–**10**. In addition, six semi-synthetic derivatives **3a**–**c**, **4b**, **5a** and **6a** were prepared by allylation and acetonidation of soyauxinium nitrate (**5**), edulinine (**3**), ribalinine (**4**) and arborinine (**6**). The structures and spectroscopic data of five of them are reported herein for the first time. The suggested mechanism for the formation of the new *N*-allylindolopyridoquinazoline **5a** is presented. The structures of natural and derived compounds were determined employing extensive NMR and MS techniques. The absolute configuration of stereogenic centers in compounds **2**–**4** were determined using NOESY technique and confirmed by the single-crystal X-ray diffraction (SC-XRD) technique. The use of SC-XRD further enabled us to carry out a structural revision of soyauxinium chloride recently isolated from the same plant to soyauxinium nitrate (**5**). The TAE, fractions, compounds **1**–**7** and **9**, and semi-synthetic derivatives **3a**–**c**, **4b**, **5a** and **6a** were evaluated for their cytotoxic activity towards the cervix carcinoma cell line KB-3-1. No significant activity was recorded for most of the compounds except for **9**, which showed moderate activity against the tested cancer cell lines.

## 1. Introduction

*Araliopsis soyauxii* Engl. [syn.*Vepris soyauxii* (Engl.) Mziray] (Rutaceae) is a rich source of new bioactive substances. Our previous reports provided valuable insights on the biology and chemistry of the studied plant, which contains different types of alkaloids, including quinazoline, furoquinoline, indolopyridoquinazoline, and oxazole [1,2,3] examples, as almost every other species of the genus *Araliopsis* does. Furoquinoline-like alkaloids form the most representative class of compounds, with more than twenty derivatives already isolated in the genus and five encountered in the studied species. These compounds are reputed for their cytotoxicity. They include 5-methoxymaculine, 5,8-dimethoxymaculine, 4,5,6,7,8-pentamethoxyfuroquinoline, skimmianine, flindersiamine, maculine and kokusaginine, which showed potent activity against the A2780 cell line with IC_50_ values ranging from 2.0 to 4.0 µM [4,5]. Their antibacterial and antioxidant activities were also reported [6,7]. However, none of the authors have applied a well-known total alkaloid extraction procedure to the crude plant extract before any isolation and purification of the alkaloids.

## 2. Results and Discussion

Herein we report the isolation and structural characterization of ten natural compounds from the total alkaloid extract of *A. soyauxii,* including a new indolopyridoquinazoline and the structural revision of soyauxinium chloride [1,2]. Furthermore, six semi-synthetic alkaloid derivatives were prepared. The crude extracts, fractions, isolated compounds, and the semi-synthetic alkaloid derivatives were investigated for their cytotoxic activity against the cervix carcinoma cell line KB-3-1.

**Figure 1:**
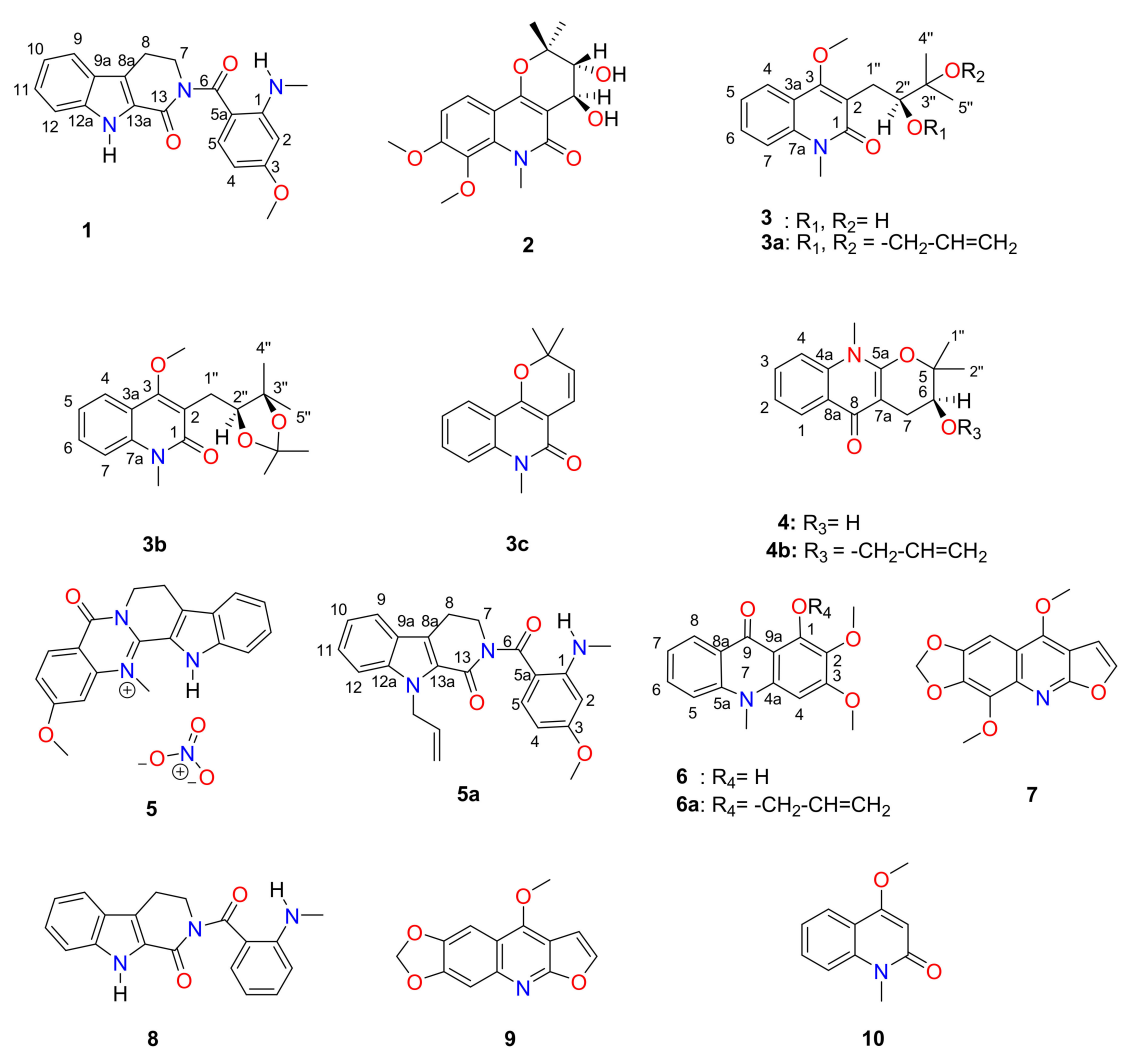
Chemical structures of compounds **1**–**10**.

The total alkaloid extract of *A.* *soyauxii* was subjected to open silica gel column chromatography and on a normal-phase medium pressure liquid chromatography (NP-MPLC), affording ten compounds **1**–**10**, including the new indolopyridoquinazoline-type alkaloid **1** (Figure 1). Six semi-synthetic derivatives (compounds **3a**–**c**, **4b**, **5a** and **6a**) were prepared by allylation and acetonidation of some of the isolates. The isolated alkaloids and synthetic derivatives structures were determined by extensive interpretation of the NMR, MS, and SC-XRD data.

### 2.1. Structural Identification of Compounds

**Figure 2:**
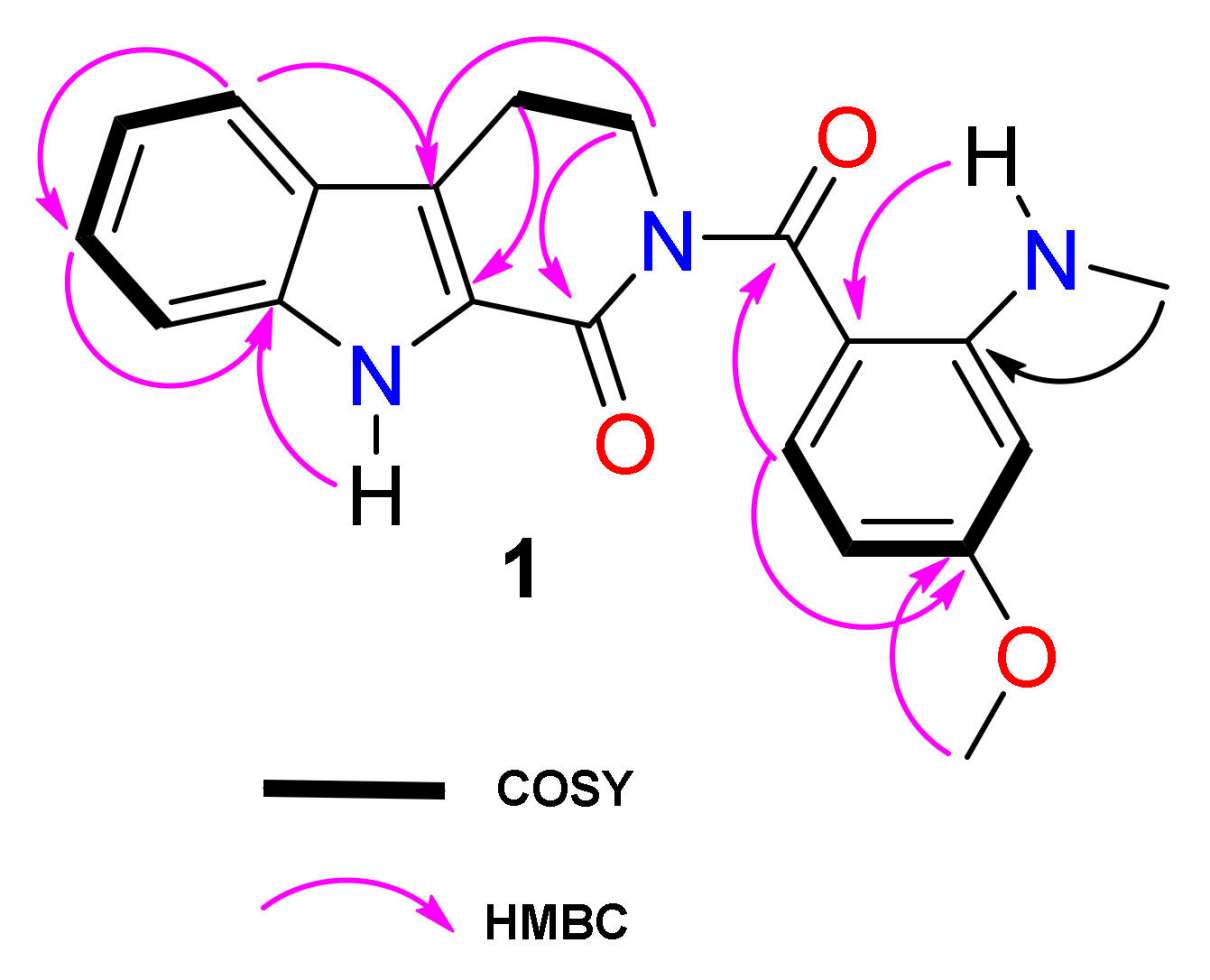
COSY and key HMBC correlations of compound **1**.

Compound **1** was isolated as yellow needles soluble in CDCl_3_. Its molecular formula C_20_H_19_N_3_O_3_ was deduced from its HRESIMS (Figure S1), which showed the sodium adduct ion peak at *m*/*z* 372.1313 (calcd. *m*/*z* 372.1318 for C_20_H_19_N_3_O_3_Na). The ^1^H-NMR spectrum (Figure S2, Table 1) of compound **1** exhibited an exchangeable signal at *δ_H_* 9.69 (1H, *brs*, NH), two triplets at δ 4.15 (*J* = 6.4 Hz) and 3.20 (*J* = 6.4 Hz), respectively characteristic of the C-7 and C-8 methylene protons of an indoloquinazoline alkaloid [1]. Several ^13^C-NMR and ^1^H-NMR resonances (Figures S2–S4) were ascribed to the indole moiety including those of an *ortho*-disubstituted aromatic ring at *δ_H_/*δ_C_7.63 (d, *J* = 8.0 Hz, H–9)/120.7; 7.16 (t, *J* = 8.0 Hz, H–10)/120.7; 7.28 (t, *J* = 8.0 Hz, H–11)/126.0 and 7.14 (t, *J* = 8.0 Hz, H–12)/113.0. The quaternary carbons were found at *δc* 122.3 (C-8a), 125.0 (C-9a), 138.4 (C-12a) and 126.4 (C-13a) [1]. Two aliphatic methylene groups resonated at *δ_H_/δ_C_* 4.15 (t, *J* = 6.4 Hz, H–7) / 47.8 and 3.20 (t, *J* = 6.4 Hz, H–8)/21.3. These were assigned to a six-membered ring with a carbamide group at *δ*c 162.1 (C-13) *ortho*-fused to the indole moiety [1]. The fused rings were confirmed by HMBC interactions of H-7 (*δ_H_* 4.15), H-8 (*δ_H_* 3.20), H-10 (*δ_H_* 7.16) and H-11 (*δ_H_* 7.28) to carbons C-8a (*δc* 122.3), C-13a (*δc* 126.4), C-9a (*δc* 125.0) and C-11(*δc* 126.0) (Figure 2).

The NMR spectra (Figures S2–S6) also showed resonances of another exchangeable proton of an amine at 7.80 (1H, q, *J* = 5.0 Hz) linked to an aminomethyl group at *δ_H_/δ_C_* 2.96 (3H, d, *J* = 5.0 Hz, NH-CH_3_)/29.7. An ABX system of a trisubstituted aromatic ring was observed at *δ_H_/δ_C_* 6.18 (1H, d, *J* = 2.4 Hz, H–2) / 94.8; 6.11 (1H, dd, *J* = 9.0, 2.4 Hz, H–4)/102.2 and 7.45 (1H, d, *J* = 9.0 Hz, H–5)/135.5. The spectra also displayed signals of a methoxy group at *δ_H_/δ_C_* 3.81(3H, s)/55.3, which was located on the trisubstituted aromatic ring based on the HMBC correlation (Figure 2) between the methoxy proton at *δ_H_* 3.81 and C-3 (*δ_C_* 165.4). Both moieties of the tri-fused rings and the trisubstituted aromatic were linked through a second carbamide at *δ*c 175.1 (C-6) as confirmed by the long-range interaction (^3^*J*) of H–5 and H-7 to the carbamide C–6 on HMBC spectrum (Figure S7).

Therefore, the structure of **1** was unambiguously determined as a methoxylated derivative of rhetsinine (**8**), an indolopyridoquinazoline-like alkaloid also reported in this study. To fully characterize compound **1**, it was subjected to *SC-XRD analysis*.

Other compounds were identified as: edulinine (**3**), arborinine (**6**), flindersiamine (**7**), rhetsinine (**8**), maculine (**9**), 4-methoxy-1-methyl-2(1*H*)-quinolinone (**10**) and *N*-methylflindersine (**3c**) [1,6,8]. Their structures were determined by comparing their NMR data with those reported in the literature.

### 2.2. SC-XRD Analyses

**Figure 3:**
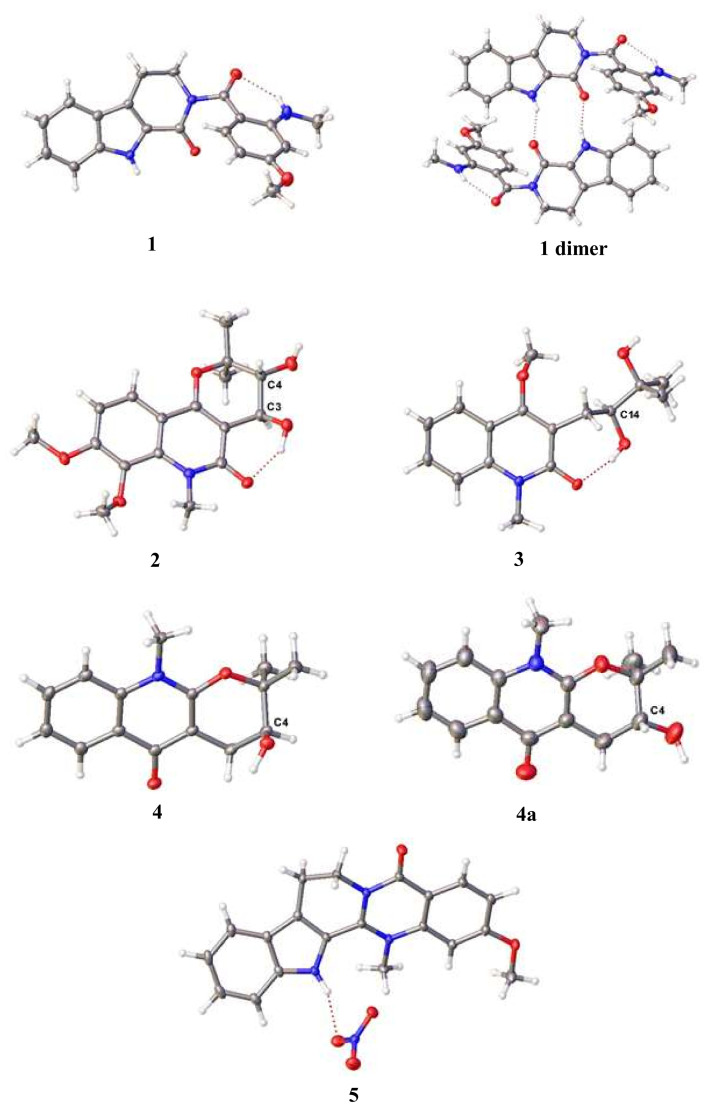
Molecular structures of the crystals of **1**–**4**, **4a** and **5** in ORTEP style at 50% probability level.

Suitable crystals of **1**, obtained by recrystallization by slow evaporation from dichloromethane, were applied to SC-XRD. The crystal structure is shown in Figure 3. Indeed, the phenyl unit is rotated 62.3 (1) ^o^ from the indole unit, as a result building a hydrogen bond between the bridged carbonyl and the methylamine proton. In that stable conformation, the N-H^…^O angle is 134 (3) ^o,^ and the N^…^O distance is 2.80(1) Å. This interaction could also explain the downfield resonance of the amine proton NHCH_3_ at C-1. The *peri*-labile proton of the indole moiety and the carbonyl group are involved in an intermolecular interaction that forms hydrogen-bonded dimers (Figure 3, 1dimer). Thus, compound 1 was fully characterized as 3-methoxyrhetsinine, an unreported indolopyridoquinazoline alkaloid to which the trivial name soyauxinine was proposed.

The crystal structure of racemic (±)-araliopsinine (**2**) reveals the opposite chirality of the two neighbouring asymmetric carbon atoms. It crystallizes as a racemate in the space group *P2_1_/c*. Optically pure (*S*)-edulinine (**3**) crystallizes in space group C2. The asymmetric unit contains two molecules of edulinine, and both show the chirality of *S* at the asymmetric carbon atom. One of these two molecules shows a positional disorder of the methyl diol unit with a ratio 57:43. This disorder contains the asymmetric carbon atom, but both disordered atoms show the chirality of *S*. The chirality is confirmed by a Flack parameter which refines to 0.06(6). In Figure 3, only the non-disordered molecule is shown. (*S*)-Ribalinine (**4**) crystallizes in the space group *P2_1_2_1_2_1_*. Its stereochemistry *S* was confirmed with a Flack parameter of 0.00(7). Additionally, ribalinine could be obtained as a racemate (**4a**) in the centrosymmetric space group *P2_1_/n*.

In Figure 3, only the asymmetric carbon atoms are labelled. Compounds **2** and **4a** are racemic mixtures, the shown enantiomers in this figure are arbitrary due to centrosymmetric space groups.

We recently published a contribution to the taxonomy and chemistry of *A. soyauxii*. We reported the isolation of a new mesomeric form of quaternary indoloquinazoline alkaloid named soyauxinium chloride [1,2]. At that time, neither the NMR nor the MS data (Supplementary Materials) were enough to finalize the structure. Nevertheless, we tentatively assigned chloride as the counter ion of our compound because of previous pieces of evidence in the literature [9,10]. However, compound **5** turned out to be soyauxinium nitrate as it crystallizes in the space group *P2_1_/c* (Figure 3). Therefore, the previously published soyauxinium chloride structure (Figures S28–S35) [1,2] should be revised to soyauxinium nitrate (**5**). The presence of nitrate as counter-ion is not surprising as nitrate ions (NO_3_^−^) are assimilated by plants from the soil for nutritional, environmental, or physiological purposes [11].

### 2.3. Semi-Synthesis of Allylated and Acetonitaded Derivatives of Some Naturally Isolated Alkaloids

**Scheme 1:**
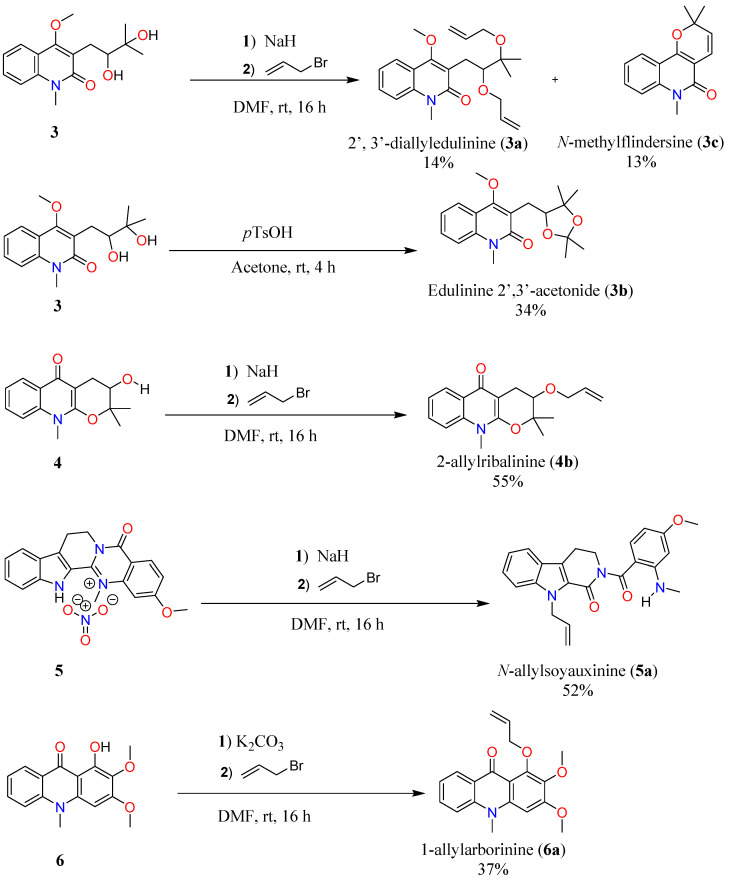
Semi-synthesis of **3a**–**6a** by allylation or acetonidation reactions of **1, 3, 4** and **6**.

Barron and co-workers observed that the addition of hydrophobic groups increases the lipophilicity of molecules, therefore enhancing the cytotoxicity activity [12]. Based on this observation, allylation and acetonidation reactions were performed on soyauxinium nitrate (**5**), edulinine (**3**), ribalinine (**4**) and aborinine (**6**) which led to six semi-synthetic derivatives, among which five (compounds **3a, 3b, 4b, 5a** and **6a**) are reported here for the first time (Scheme 1).

2′,3′-Diallyledulinine (**3a**, 2.4mg, 14%) (Figures S8–S12) and *N*-methylflindersine (**3c**, 2.1 mg, 13%) (Figures S16–S18) were prepared from the reaction between edulinine (**3**) and allyl bromide with sodium hydride as base. They were obtained as yellowish oils isolated by preparative TLC of the dried mixture eluting with petroleum ether/EtOAc (70:30).

**Scheme 2:**
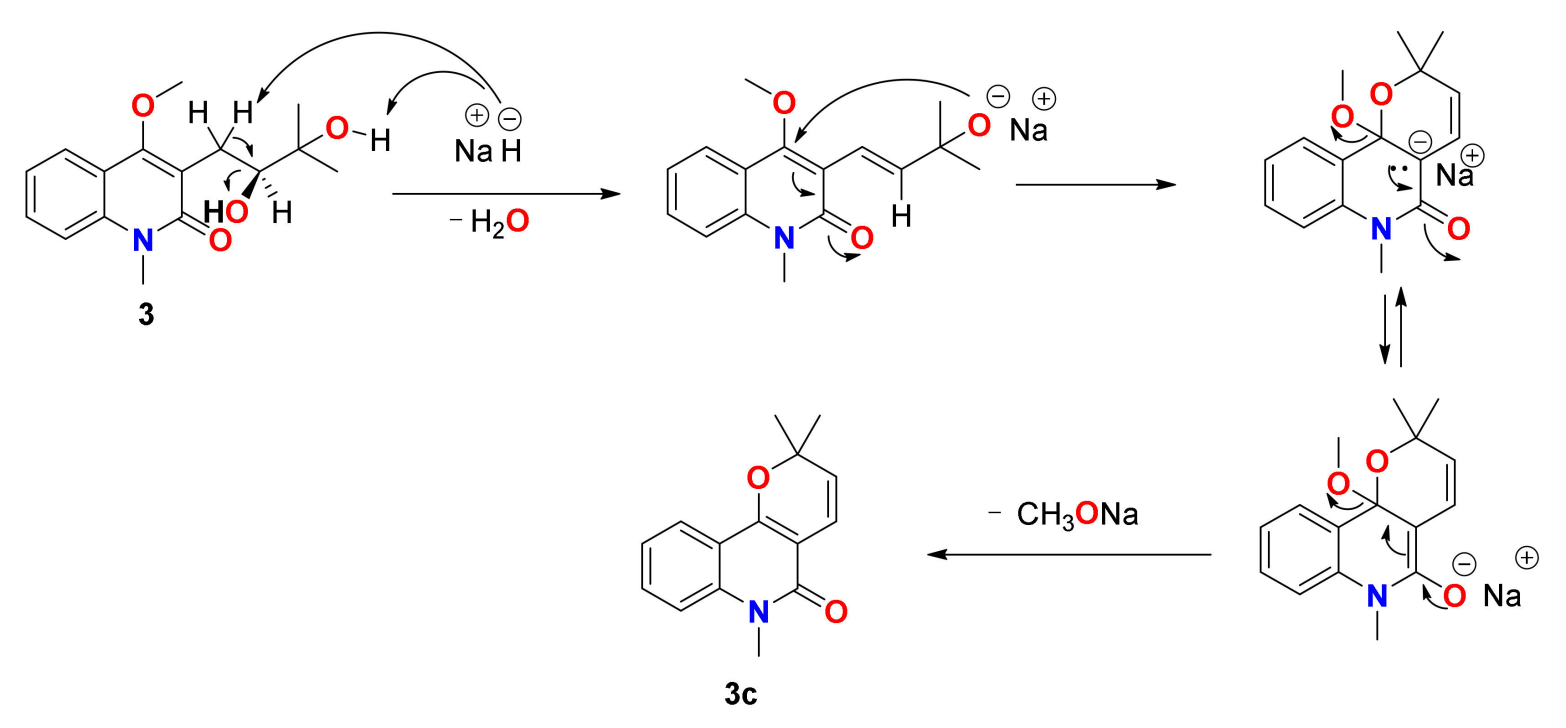
Proposed mechanism for the formation of compound **3c**.

The formation of **3c** can be explained by the following mechanism of a nucleophilic substitution on an unsaturated carbon favoured by the presence of a carbonyl group, as shown in Scheme 2.

Edulinine-2′,3′-acetonide (**3b**, 10.2 mg, 34%) (Figures S13–S15) was prepared from edulinine (**3**) and *p*-toluene sulfonic acid and obtained as white needles.

Reactions between ribalinine (**4**) and sodium hydride with allyl bromide as reagent gave 2-allylribalinine (**4b**, 6.2 mg, 55%) as a yellow oil (Figures S19–S21) while arborinine (**6**) and potassium carbonate afforded semisynthetic 1-allylarborinine (**6a**, 1.1 mg, 37%) also obtained as a yellow oil (Figures S25–S27).

**Scheme 3:**
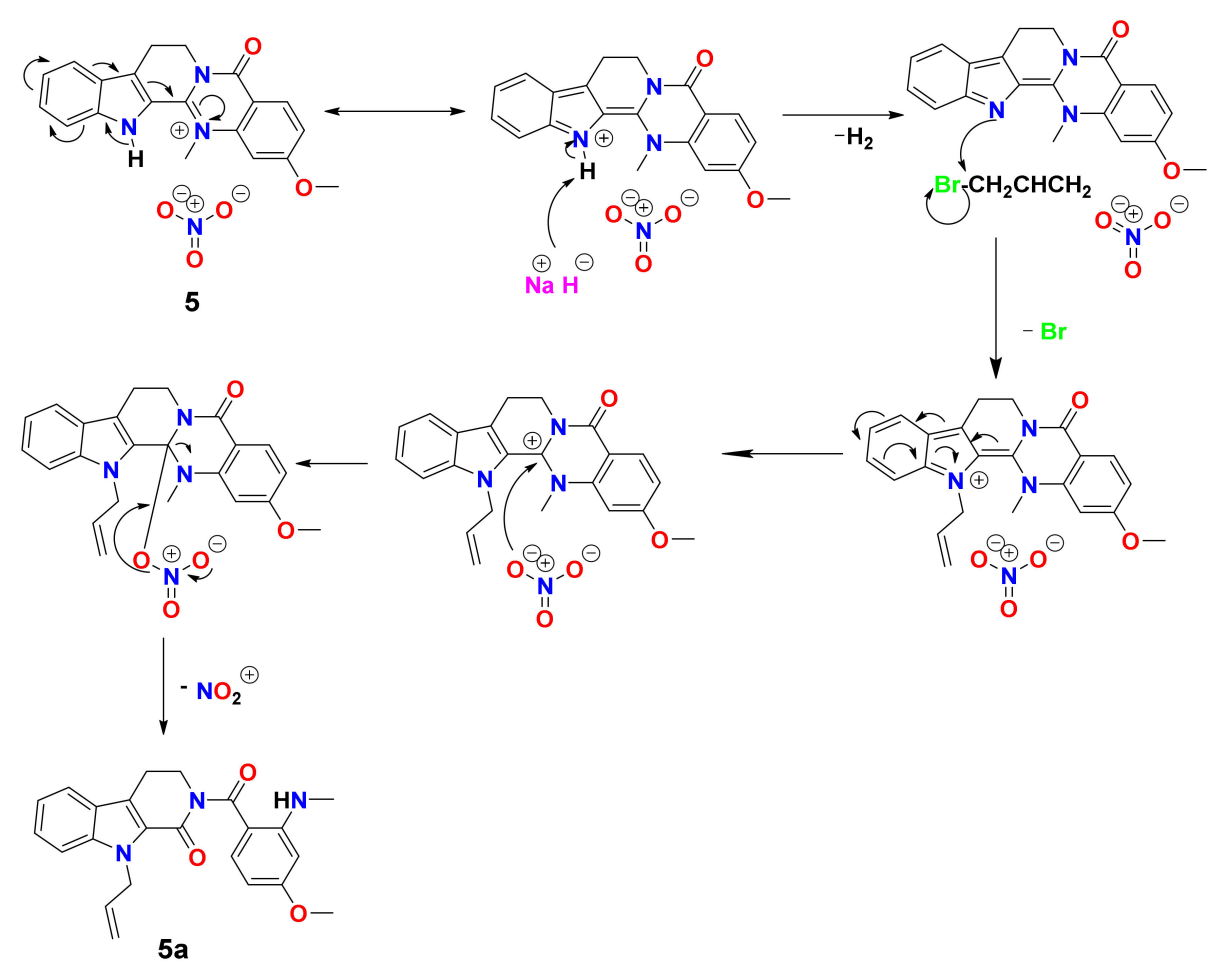
Proposed mechanism for the formation of compound **5a**.

The same protocol when applied to soyauxinium nitrate (**5**) afforded *N*-allylsoyauxinine (**5a**, 10.2 mg, 52%) (Figures S22–S24). The mechanism for the formation of compound **5a** is shown in Scheme 3.

### 2.4. Cytotoxic Activity of the Extract, Fractions, and Isolated Compounds

The total alkaloid extract of the stem bark of *A. soyauxii* (TAE), fractions obtained from TAE (FTAE1-FTAE3), natural compounds **1**–**7** and **9**, and semi-synthetic derivatives **3a–c, 4b, 5a, 6a** were evaluated for their anticancer properties against the cervix carcinoma cell line (KB-3-1) as described in previous reports [13,14,15,16]. Extracts and fractions were inactive. Maculine (**9**) showed moderate cytotoxic activity against KB-3-1 with IC_50_ of 10 µM compared to griseofulvin (IC_50_ = 17–21 μM), used as a reference drug. Other compounds were found inactive against the same cancer cell line. Arborinine (**6**) has been previously proven to exhibit toxicity against HeLa, MCF-7, and A431 cancer cell lines with IC_50_ values of 1.84, 11.74, and 12.95 µM, respectively [16]. Likewise, maculine (**9**) was also found to display moderate cytotoxic activity (IC_50_ = 100 μM) against the colorectal HT-29 adenocarcinoma cells [1]. The difference in efficacy of these compounds could be related to the sensitivity of cancer cells towards each compound. Maculine is known to be more harmful to cervical tumour than colorectal cells, while arborinine presents a similar response towards epidermal and breast cancer cells. However, compound **6** was active against cervical cancer cells HeLa but inactive towards KB-3-1 cells [17]. As a result, the semi-synthetic derivatives, *N*-allylsoyauxinine (**5a**) and 1-allylarborinine (**6a**), were transparent compared to soyauxinium nitrate (**5**) arborinine (**6**), suggesting that the moderate response of compounds **5** and **6** might be related to their labile heteroatom protons. Semi-synthetic compounds 2′, 3′-diallyledulinine (**3a**), *N*-methylflindersine (**3c**), edulinine-2′,3′-acetonide (**3b**), 2-allylribalinine (**4b**) and compounds edulinine (**3**), ribalinine (**4**) were also inactive against the KB-3-1 cancer cells. The obtained results indicate that the increase of lipophilicity of the studied alkaloids by the addition of hydrophobic groups is not enough to enhance their cytotoxicity activity. Results are shown in Table 2.

## 3. Materials and Methods

### 3.1. General Information

Electrospray ionization (ESI) mass spectra were recorded on a 1200-series HPLC-system or a 1260-series Infinity II HPLC-system (Agilent Technologies, Santa Clara, CA, USA) with binary pump and integrated diode array detector coupled to an LC/MSD-Trap-XTC-mass spectrometer (Agilent Technologies) or an LC/MSD Infinity lab LC/MSD (G6125B LC/MSD). High-resolution mass spectra were recorded on a Micromass-Q-TOF-Ultima-3-mass spectrometer (Waters, Milford, MA, USA) with Lock Spray-interface and a suitable external calibrant. UV-Vis spectra were recorded on an Evolution 201 UV-Visible Spectrophotometer (Thermo Scientific, Waltham, MA, USA) and infrared (IR) spectra on a Tensor 27 FTIR-spectrometer (Bruker, Billerica, MA, USA) equipped with a diamond ATR unit and are reported in terms of frequency of absorption in cm^–1^. The NMR spectra were recorded on a Bruker Avance III 500 HD (^1^H: 500 MHz, ^13^C: 126 MHz) or Avance 600 (^1^H: 600 MHz, ^13^C: 151 MHz) spectrometer. Chemical shifts *δ* (ppm) are reported relative to residual solvent signal and/or tetramethylsilane (TMS). 2D spectra (COSY, HMQC, HMBC) and DEPT-135 spectra were used for signal assignment. The NMR spectra were recorded on a Bruker Avance-III (^1^H-NMR: 600 MHz and ^13^C-NMR: 151.1 MHz) spectrometer. Column chromatography was carried out on silica gel 230–400 mesh and silica gel 70–230 mesh (Merck, Darmstadt, Germany). Automated column chromatography was also performed on a Reveleris^®^ X2 system (Büchi, Flawil, Switzerland) equipped with a binary pump and ELSD detector using a flash up column of 4 g, 12 g, and 24 g, depending on the sample mass or a Snap Ultra C18 column (Biotage, Uppsala, Sweden) with a gradient at various flow rate. Thin-layer chromatography (TLC) was performed on Merck precoated silica gel 60 F_254_ aluminum foil and was revealed using a UV lamp (254–365 nm) and 10% H_2_SO_4_ reagent followed by heating.

### 3.2. Plant Material

The stem bark of *A. soyauxii* Engl. was collected at UNALOR, Kumba, South-West Region of Cameroon in January 2016. The plant material was identified by the botanist Mr. NANA Victor of the Cameroon National Herbarium where a voucher specimen (Ref. 38959 HNC) has been deposited.

### 3.3. Extraction and Isolation

The stem bark crude extract of *A. soyauxii* (125.7 g) was dissolved in a sulfuric acid solution (c = 2 M). The acidified extract was then washed with EtOAc to afford the aqueous and organic layers, which were separated. An ammonia solution was used to basify the aqueous layer to obtain an organic solution with a pH value of 12. The organic solution containing alkaloids was washed with DCM to afford the total alkaloid extract (10.5 g). A portion of the total alkaloid extract (TAE) (10.3 g) was fractionated on a silica gel column eluted with a stepwise gradient of CH_2_Cl_2_-MeOH mixtures of increasing polarity then with MeOH. A total of 50 fractions (ca. 100 mL each) were collected as follows: CH_2_Cl_2_ (1–4), CH_2_Cl_2_/MeOH 5% (5–12), CH_2_Cl_2_/MeOH 10% (13–20), CH_2_Cl_2_ /MeOH 20% (21–29), CH_2_Cl_2_/MeOH 30% (30–37), CH_2_Cl_2_/MeOH 50% (38–46) and CH_2_Cl_2_/MeOH 70% (47–50). Three main fractions FTAE1 (1–21, 1.5 g); FTAE2 (22–32, 5.7 g), and FTAE3 (33–50, 1.7 g), were obtained based on their TLC profiles. Part of fraction FTAE1 (1.4 g) was further fractionated on a normal-phase medium pressure liquid chromatography (NP-MPLC) with a stepwise gradient of EtOAc-MeOH mixtures to afford 60 sub-fractions of 18 mL each. Based on their analytical TLC profiles, they were combined into five major fractions labelled FTAE1A (1–15, 250.8 mg), FTAE1B (16–26, 400.5 mg), FTAE1C (27–40, 350.3 mg), and FTAE1D (41–60, 120.4 mg). Maculine (**9**; 3.5 mg) and 4-methoxy-1-methyl-2(1*H*)-quinolinone (**10**; 2.6 mg) were obtained from the purification of fraction FTAE1B over a silica gel column with isocratic *n*-hexane-EtOAc 20%. Fraction FTAE1C was subjected to column chromatography using silica gel column with *n*-hexane-EtOAc 30% to afford flindersiamine (**7**; 2.8 mg), while *S*-edulinine (**3**; 72.6 mg) and (*4S*)-ribalinine (**4**; 15.6 mg) were obtained from the purification of fraction FTAE1D with *n*-hexane-EtOAc 40% and *n*-hexane-EtOAc 45% respectively. Part of fraction FTAE2 (5.0 g) was further fractionated on NP-MPLC with a stepwise gradient of EtOAc-MeOH mixtures to afford 40 sub-fractions of 18 mL each. Based on their analytical TLC profiles, these sub-fractions were grouped into three major fractions labelled FTAE2A (1–12, 1.2 g), FTAE2B (13–25, 1.5 g), and FTAE2C (26–40, 1.1 g). Further purification of fractions FTAE2B and FTAE2C gave arborinine (**6**; 3.2 mg) and (+)/(−)-araliopsinine (**2**; 5.2 mg) at *n*-hexane-EtOAc 25% and *n*-hexane-EtOAc 35% respectively. Part of fraction FTAE3 (1.6 g) was also flashed on NP-MPLC with a stepwise gradient of CH_2_Cl_2_-MeOH mixtures to afford 30 sub-fractions of 18 mL each. Based on their analytical TLC profiles, these sub-fractions were combined into two major fractions labelled FTAE3A (1–19, 800.7 mg) and FTAE3B (20–40, 450.6 mg). Similar purification methods applied to FTAE3A afforded rhetsinine (**8**; 1.1 mg) and soyauxinine (**1**; 3.1 mg), while sub-fraction FTAE3B gave soyauxinium nitrate (**5**; 30.8 mg).

### 3.4. X-ray Diffraction Analyses

All XRD data of compounds **1**, **2**, **3**, **4**, **4a**, and **5** were collected on a SuperNova diffractometer (Rigaku, Tokyo, Japan ). Using Olex2 [18], the structures were solved and refined with the ShelX program package [19] using direct methods and least-squares minimization. The crystal of soyauxinine (**1**) was pseudo-merohedrically twinned; the second domain was rotated by 180° around 100 (reciprocal) with a ratio 66:34. Both domains were taken into account for data reduction and refinement. The asymmetric unit of edulinine (**3**) contains two molecules with the same chirality *S* at C14 resp. C35A/B, the Flack parameter refines to 0.06(6). The second molecule shows a disorder with a ratio 57:43 from C34 to C40. Except in this structure, the hydrogen atoms have been refined isotropically. CCDC 2039617 (**1**), 2039618 (**2**), 2069085 (**3**), 2039619 (**4**), 2069087 (**4a**) and 2069086 (**5**) contain the supplementary crystallographic data for this paper. This data is freely available at the Cambridge Crystallographic Data Centre via www.ccdc.cam.ac.uk/conts/retrieving.html (accessed on 20 November 2021).

### 3.5. Cytotoxic Activity of the Extract, Fractions, Isolates, and Semi-Synthetic Derivatives

The cytotoxicity assay of some isolated compounds was performed on human cervix carcinoma (KB-3-1) cancer cell lines using the previously reported method [20]. The KB-3-1 cells were cultivated as a monolayer in Dulbecco’s modified Eagle medium (DMEM) with glucose (4.5 g/L), l-glutamine, sodium pyruvate and phenol red, supplemented with 10% (KB-3-1) foetal bovine serum (FBS). On the day before the test, the cells (70% confluence) were detached with trypsin-ethylenediamine tetraacetic acid (EDTA) solution (0.05%; 0.02% in DPBS) and placed in sterile 96-well plates in a density of 10 000 cells in 100 μL medium per well. The dilution series of the compounds were prepared from stock solutions in DMSO with concentrations of 1 mM or 10 mM. The stock solutions were diluted with culture medium (10% FBS [KB-3-1]) at least 50 times. Some culture medium was added to the wells to adjust the volume of the wells to the required dilution factor. The dilution prepared from stock solution was added to the wells, and each concentration was tested in six replicates. The control contained the same concentration of DMSO as the first dilution. After incubation at 37 °C and 5.3% CO_2_-humidified air for 72 h, 30 μL of an aqueous resazurin solution (175 μM) was added to each well. The cells were incubated for 6 h at the same conditions. Thereafter, the fluorescence was measured. The excitation was affected at a wavelength of 530 nm, whereas the emission was recorded at 588 nm. The IC_50_ were recorded as the values equal to the drug concentrations, at which vitality was 50%, and was calculated as a sigmoidal dose-response curve using GraphPad Prism 4.03.

### 3.6. Preparation of the Semis-Ynthetic Derivatives

#### 3.6.1. Allylation of Edulinine

Edulinine (**3**, 15.0 mg, 0.051 mmol) was dissolved in dimethylformamide (300 µL), and sodium hydride (12.5 mg, 0.313 mmol) was added to the medium. After complete dissolution, allyl bromide (11.5 µL, 0.133 mmol) was added and stirred at room temperature for 16 hrs. The reaction progress was monitored with LC-MS, and after the complete conversion of starting material, the reaction was quenched with water. Then, the organic layer was washed with brine water and reduced to dryness using a rotary evaporator. 2′, 3′-diallyledulinine (**3a**, 2.4mg, 14.2%) and *N*-methylflindersine (**3c**, 2.1 mg, 13%) were obtained

*2, 3*-*Diallyledulinine* (**3a**): Yellowish oil; ^1^H-NMR (500 MHz, CD_3_OD): δ_H_ (mult, *J* in Hertz) 7.88 (dd, *J* = 8.0, 1.4 Hz, H-4), 7.63 (ddd, *J* = 8.4, 6.9, 1.5 Hz, H-6), 7.59 (dd, *J* = 8.6, 1.1 Hz, H-7), 7.34 (ddd, *J* = 8.1, 6.9, 1.2 Hz, H-5), 5.86 (ddt, *J* = 17.2, 10.4, 5.2 Hz, H-2′), 5.54 (ddt, *J* = 17.3, 10.4, 5.7 Hz, H-5′), 5.22 (dq, *J* = 17.2, 1.9 Hz, H-3′), 5.01 (dq, *J* = 10.5, 1.7 Hz, H-3′), 4.94 (dq, *J* = 17.2, 1.7 Hz, H-3′), 4.81 (ddt, *J* = 10.4, 2.3, 1.3 Hz, H-3′), 4.06 (ddt, *J* = 7.0, 5.3, 1.7 Hz, H-1′), 4.00 (s, 3-OCH_3_), 3.92 (m, H-1′), 3.74 (s, NCH_3_), 3.35 (s, H-2′’), 3.03 (dd, *J* = 13.4, 9.8 Hz, H-1′’), 2.82 (dd, *J* = 13.4, 3.7 Hz, H-1′’), 1.30 (s, H-4′’), 1.30 (s, H-5′’); ^13^C-NMR (125 MHz, CD_3_OD): δ_C_ 165.8 (C-1), 163.4 (C-3), 140.2 (C-7a), 137.4(C-2′), 136.4(C-2′), 131.6 (C-6), 124.5 (C-5), 123.5 (C-4), 121.8 (C-2), 119.0 (C-3a), 116.3 (C-3′), 115.8(C-7), 115.4, 83.3 (C-3′’), 79.4 (C-2′’), 64.1 (C-1′), 62.8 (C-1′), 30.3 (NCH_3_), 21.8 (C-4′’), 23.4 (C-5′’), 21.8 (C-5′’); HRESIMS [M + Na]^+^ at *m*/*z* 394.1978 (calcd. *m*/*z* 394.1988 157 for C_22_H_29_NO_4_Na).

*N*-*Methylflindersine* (**3c**): Yellowish oil; ^1^H-NMR (500 MHz, CDCl_3_); δ 7.96 (dd, *J* = 8.0, 1.6 Hz, H-4), 7.32 (m, H-5), 7.54 (ddd, *J* = 8.6, 7.1, 1.6 Hz, H-6), 7.23 (ddd, *J* = 8.1, 7.1, 1.0 Hz, H-7), 6.75 (d, *J* = 9.9 Hz, H-2′), 5.53 (d, *J* = 9.9 Hz, H-3′), 3.69 (s, NCH_3_), 1.51 (s, 6H); ^13^C-NMR (125 MHz, CDCl_3_): δ_C_ 161.1 (C-1), 118.0 (C-2), 155.3 (C-3), 114.1 (C-3a), 123.2 (C-4), 126.4 (C-5), 130.9 (C-6), 116.2 (C-7), 139.4 (C-7a), 78.6 (C-1′), 118.0 (C-2′), 126.4 (C-3′), 115.4, 28.3 (C-4′), 29.3 (NCH_3_); HRESIMS [M + Na]^+^ at *m*/*z* 264.0999 (calcd. *m*/*z* 264.0995 for C_15_H_15_NO_2_Na).

#### 3.6.2. Acetonidation of Edulinine

Edulinine (**3**, 26.2. mg, 0.090 mmol) was dissolved in acetone (3.0 mL), and a catalytic amount of *p*-toluene sulfonic acid (23.3 mg, 0.135 mmol) was added to the reaction mixture and was allowed to stir for four hours at room temperature. The reaction progress was monitored with LC-MS, and after the complete conversion of starting the material, the reaction was quenched with water. The reaction mixture was dissolved in water and extracted with chloroform. The organic layer was washed with brine water and reduced to dryness using a rotary evaporator. *Edulinine-2′,3′-acetonide* (**3b**, 10.2 mg, 34%) was thus obtained. Yellow oil; ^1^H-NMR (500 MHz, CDCl3): δ_H_ (mult, *J* in Hertz) 7.85 (dd, *J* = 8.0, 1.6 Hz, H-4), 7.55 (ddd, *J* = 8.6, 7.1, 1.6 Hz, H-6), 7.38 (dd, *J* = 8.6, 0.9 Hz, H-7), 7.27 (m, H-5), 4.27 (dd, *J* = 9.4, 3.0 Hz, H-1′), 3.03 (dd, *J* = 13.3, 9.4 Hz, H-2′), 2.70 (dd, *J* = 13.3, 3.0 Hz, H-1′), 4.04 (s, 3-OCH_3_), 3.73 (s, NCH_3_), 1.36 (s, 4′-CH_3_), 1.40 (s, 5′-CH_3_), 1.25 (s, 4′-CH_3_)1.26 (s, 5′-CH_3_); ^13^C-NMR (125 MHz, CDCl_3_): δ_C_ 163.9 (C-1), 161.6 (C-3), 139.4 (C-7a), 130.4 (C-6), 123.8 (C-5), 122.0 (C-4), 119.9 (C-3a), 117.9 (C-2′’), 114.3 (C-7), 106.6 (C-2), 80.6 (C-3′), 80.4 (C-2′), 62.6 (3-OCH_3_), 29.7 (NCH3), 28.7 (1′’-CH_3_), 27.0 (3′’-CH_3_), 26.0 (5′-CH_3_), 25.9 (4′-CH_3_), 23.2 (1′-CH2); HRESIMS [M + Na]^+^ at *m*/*z* 354.1681 (calcd. *m*/*z* 354.1675 for C_19_H_25_NO_4_Na).

#### 3.6.3. Allylation of Ribalinine

Ribalinine (**4**, 10.0 mg, 0.038 mmol) was dissolved in dimethylformamide (300 µL), and sodium hydride (6.6 mg, 0.165 mmol) was added to the medium. After complete dissolution, allyl bromide (4.3 µL, 0.050 mmol) was added and stirred at room temperature for 16 h. The reaction progress was monitored with LC-MS, and after the complete conversion of the starting material, the reaction was quenched with water and reduced to dryness on a lyophilisator, and *2-allylribalinine* (**4b**, 6.2 mg, 55%) was thus obtained. Yellow oil; ^1^H-NMR (500 MHz, CD_3_OD): δ_H_ (mult, *J* in Hertz) 7.86 (dd, *J* = 8.1, 1.6 Hz, H-1), 7.23 (ddd, *J* = 8.6, 7.0, 1.6 Hz, H-3), 7.12 (m, H-4), 6.91 (ddd, *J* = 8.0, 6.9, 1.0 Hz, H-2), 5.44 (m, H-2′), 4.82 (dq, *J* = 17.2, 1.6 Hz, H-3′), 4.72 (dq, *J* = 10.4, 1.4 Hz, H-3′), 3.58 (ddt, *J* = 12.8, 6.1, 1.4 Hz, H-6), 3.17 (t, *J* = 5.4 Hz, H-1′), 2.46 (dd, *J* = 16.7, 4.9 Hz, H-7), 2.33 (dd, *J* = 16.7, 5.8 Hz, H-7), 1.02 (d, *J* = 5.1 Hz, 1′-CH_3_); 1.02 (d, *J* = 5.1 Hz, 1′-CH_3_); ^13^C-NMR (125 MHz, CD_3_OD): δ_C_ 178.0 (C-8), 156.2 (C-5a), 139.8 (C-4a),135.3 (C-3),132.8 (C-2′),126.3 (C-8a), 124.1 (C-1), 123.8 (C-2), 117.7 (C-3′), 115.9 (C-4), 97.7 (C-7a), 83.2 (C-5), 75.6 (C-6), 71.3 (C-1′), 31.0 (NCH_3_), 25.6 (C-7), 22.6 (C-2′’), 22.4 (C-1′’); HRESIMS [M + Na]^+^ at *m*/*z* 322.1407 (calcd. *m*/*z* 322.1413 for C_18_H_21_NO_3_Na)

#### 3.6.4. Allylation of Soyauxinium Nitrate

Soyauxinium nitrate (**5**, 20.4 mg, 0.051 mmol) was dissolved in dimethylformamide (300 µL) and sodium hydride (6.2 mg, 0.259 mmol) was added to the medium. After complete dissolution, allyl bromide (13.4 µL, 0.155 mmol) was added and stirred at room temperature for 16 hrs. The reaction progress was monitored with LC-MS, and after the complete conversion of the starting material, the reaction was quenched with water and reduced to dryness on a lyophilisator. *N-allylsoyauxinine* (**5a**, 10.2 mg, 52%) was obtained. Yellow oil; ^1^H-NMR (600 MHz, CDCl_3_); δ_H_ (mult, *J* in Hertz) 7.78 (brs, 1-HN), 7.66 (dt, *J* = 8.1, 1.1 Hz, H-9), 7.42 (m, H-5), 7.38 (m, H-10), 7.19 (m, H-12), 6.12 (d, *J* = 6.0 Hz, H-4), 6.12 (d, *J* = 6.0 Hz, H-2), 5.94 (ddt, *J* = 17.1, 10.4, 5.2 Hz, H-2′), 5.22 (m, H-1′), 5.06 (dq, *J* = 10.2, 1.4 Hz, H-3′), 4.92 (dq, *J* = 17.1, 1.6 Hz, H-3′). 4.11 (t, *J* = 6.4 Hz, H-7), 3.20 (t, *J* = 6.4 Hz, H-8), 2.92 (d, *J* = 4.0, 1-NH_3_), 3.84 (s, 3-OCH_3_); ^13^C-NMR (125 MHz, CDCl_3_): δ_C_ 94.7(C-2), 102.4 (C-4), 108.9 (C-5a), 111.0 (C-12), 116.2 (C-3′), 120.7 (C-9), 120.9 (C-10), 123.1 (C-8a), 124.1(C-2′), 125.2 (C-9a), 126.1 (C-11), 134.0(C-13a), 135.3 (C-5), 139.6 (C-12a), 153.9 (C-1), 162.2 (C-13), 165.2 (C-3), 175.6 (C-6), 55.2 (3-OCH_3_), 47.4 (C-1′), 46.8 (C-7), 29.7 (1-NCH_3_), 21.4 (C-8); HRESIMS [M + Na]^+^ at *m*/*z* 412.1629 (calcd. *m*/*z* 412.1631 for C_23_H_23_N_3_O_3_Na).

#### 3.6.5. Allylation of Arborinine

Arborinine (**6**, 2.7 mg, 0.0095 mmol) was dissolved in dimethylformamide (300 µL), and potassium carbonate (2.5 µL, 0.047 mmol) was added to the medium. After complete dissolution, allyl bromide (3.4 mg, 0.028 mmol) was added and stirred at room temperature for 16 h. The reaction progress was monitored with LC-MS, and after the complete conversion of the starting material, the reaction was quenched with water and reduced to dryness on a lyophilisator. The reaction afforded *1-allylarborinine* (**6a**, 1.1 mg, 37%). Yellow oil; ^1^H-NMR (500 MHz; CDCl_3_): δ_H_ (mult, *J* in Hertz) 8.49 (dd, *J* = 8.0, 1.7 Hz, H-8), 7.65 (ddd, *J* = 8.6, 6.9, 1.7 Hz, H-6), 7.44 (d, *J* = 8.6 Hz, H-5), 7.24 (m, H-7), 6.62 (s, H-4), 6.29 (ddt, *J* = 16.7, 10.3, 6.0 Hz, H-2′), 5.41 (dq, *J* = 17.2, 1.6 Hz, H-3′), 5.22 (dq, *J* = 10.4, 1.3 Hz, H-3′), 4.69 (dt, *J* = 6.0, 1.4 Hz, H-1′), 4.02 (s, 3-OCH_3_), 3.90 (s, 2-OCH_3_), 3.36 (s, 3H); ^13^C-NMR (125 MHz, CDCl_3_): δ_C_ 177.6 (C-9), 158.5(C-2), 153.1 (C-1), 142.5 (C-4a), 142.3(C-5), 138.4(C-3), 133.7(C-6), 127.4(C-8), 123.7(C-8a), 121.8(C-7), 118.1(C-3′),115.1(C-5a), 112.2(C-9a), 93.5(C-4), 76.0(C-1′), 61.6(2-OCH_3_), 56.3(3-OCH_3_), 35.1(NCH_3_); HRESIMS ([M + Na]^+^) at *m*/*z* 348.1200 (calcd. *m*/*z* 348.1206 for C_19_H_19_NO_4_Na).

## 4. Conclusions

A completely different chemical profile of *Araliopsis soyauxii* was evidenced after the chemical analysis of the total alkaloid extract of its stem bark compared to previous reports on the same plant, with the isolation of ten alkaloids, including a new indolopyridoquinazoline alkaloid. This will contribute to the enrichment of the databases of organic compounds from Nature. SC-XRD was used as a powerful tool to determine the absolute and conformational stereochemistry of alkaloids and their counterions. The technique allowed us to carry out a structural revision of the previously described soyauxinium chloride to soyauxinium nitrate. The evaluation of the cytotoxic activity of *A. soyauxii* alkaloids revealed the isolated compounds to be almost inactive, except for maculine, which showed moderate activity against the KB-3-1 cancer cell line. The inactivity of the semi-synthetic derivatives after allylation and acetonidation reactions allowed us to conclude that increasing the lipophilicity of these compounds alone is insufficient to enhance their cytotoxic activity and the moderate activity of arborinine and soyauxinium nitrate might be related to their free hydroxyl and amine groups, respectively.

## Figures and Tables

**Table 1:** ^1^H-NMR data (600 MHz, CDCl_3_) and ^13^C-NMR data (150 MHz, CDCl_3_) of soyauxinine (**1**).

Position	1		
	*δ*c	*δ*_H_	HMBC
**1**	154.0		
**2**	94.8	6.18(1H, d, *J* = 2.4)	1, 3
**3**	165.4		
**4**	102.2	6.11 (1H, dd; *J* = 9.0, 2.4)	3, 5a
**5**	135.5	7.45 (1H, d, *J* = 9.0)	1, 3, 6
**5a**	109.0		
**6**	175.1		
**7**	47.8	4.15 (2H, t, *J* = 6.4)	6, 8a, 13
**8**	21.3	3.20 (2H, t, *J* = 6.4)	8a, 13a
**8a**	122.3		
**9a**	125.0		
**9**	120.7	7.63 (1H, d, *J* = 8.0)	12, 12a
**10**	120.7	7.16 (1H, t, *J* = 8.0)	9a, 12
**11**	126.0	7.28 (1H, t, *J* = 8.0)	9, 10, 12
**12**	113.0	7.14 (1H, t, *J* = 8.0)	9, 9a, 10
**12a**	138.4		
**13a**	126.4		
**13**	162.1		
**1-NCH_3_**	29.7	2.96 (3H, d, *J* = 5.0)	1
**1-NHCH_3_**		7.80 (1H, q, *J* = 5.0)	2
**3-OCH_3_**	55.3	3.81(1H, s)	3
**NH**		9.69, brs	8a, 12a

**Table 2:** IC_50_ (µM) values of cytotoxic activity of the extract, fractions, isolates, and semi-synthetic derivatives.

	IC_50_ (µM)
Samples	KB-3-1
**1**	˃50
**2**	Inactive
**3**	Inactive
**3a**	Inactive
**3b**	˃500
**3c**	˃500
**4**	Inactive
**4b**	Inactive
**5**	Inactive
**5a**	Inactive
**6**	50
**6a**	˃500
**7**	Inactive
**9**	10
**FTAE1**	Inactive
**FTAE2**	Inactive
**FTAE3**	Inactive
**TAE**	Inactive
**Griseofulvin**	17–21

TAE: Total alkaloid extract; FTAE1: Fraction 1 of total alkaloid extract; FTAE2: Fraction 2 of total alkaloid extract; FTAE3: Fraction 3 of total alkaloid extract

## Data Availability

Not applicable.

## Supplementary Materials

The following supporting information can be downloaded. Figure S1: ESI-HR Mass spectrum of **1**, Figure S2–S7: 1 and 2D NMR spectra of **1**, Figure S8: ESI-HR Mass spectrum of **3a**, Figure S9–S12: ^1^H and ^13^C-NMR spectra of **3a**, Figure S13: ESI-HR Mass spectrum of **3b**, Figure S14–S15: ^1^H and ^13^C-NMR spectra of **3b**, Figure S16: ESI-HR Mass spectrum of **3c**, Figure S17–S18: ^1^H and ^13^C-NMR spectra of **3c**, Figure S19: ESI-HR Mass spectrum of **4b**, Figure S20–S21: ^1^H and ^13^C-NMR spectra of **4b**, Figure S22: ESI Mass spectrum of **5a**, Figure S23: ^1^H-NMR spectrum of **5a**, Figure S24: ESI-HR Mass spectrum of **5a**, Figure S25: ESI-HR Mass spectrum of **6a**, Figure S26–S27: ^1^H and ^13^C-NMR spectra of **6a**, Figure S28: Mass spectrum of **5**, Figure S29: ^1^H spectrum of **5**, Figure S30: Mass spectrum of soyauxinium chloride, Figure S31–S35: 1 and 2D NMR spectra of soyauxinium chloride.

## Sample Availability

Samples of the compounds are not available from the authors.
