# Supplementary material for: Soyauxinine, a New Indolopyridoquinazoline Alkaloid from the Stem Bark of Araliopsis soyauxii Engl. (Rutaceae)
Source: Molecules. 2022 Feb 7;27(3):1104. doi: 10.3390/molecules27031104 (PMC8838469; doi:10.3390/molecules27031104)
Supplement: Supplementary file 1 [file molecules-27-01104-s001.zip › molecules-1505648-supplementary.pdf]

SUPPORTING INFORMATION

Soyauxinine, a New Indolopyridoquinazoline Alkaloid from the Stem Bark of *Araliopsis soyauxii* Engl. (Rutaceae)

Cédric Guy Tchatchouang Noulala [1,2], Judith Laure Nantchouang Ouete [1], Albert Fouda Atangana [3], Gabin Thierry Bitchagno Mbahbou [2,4], Ghislain Wabo Fotso [1,*], Hans-Georg Stammler [5], Bruno Ndjakou Lenta [6], Emmanuel Ngeufa Happi [7,*], Norbert Sewald [2,*], Bonaventure Tchaleu Ngadjui [1]

[1] Department of Organic Chemistry, Faculty of Science, University of Yaoundé 1, Yaoundé P.O. Box 812, Cameroon; cedric.noulala@hotmail.fr (C.G.T.N.); ouetejudit@yahoo.fr (J.L.N.O.); ngadjuibt@yahoo.fr (B.T.N.)

[2] Organic and Bioorganic Chemistry, Department of Chemistry, Bielefeld University, 33501 Bielefeld, Germany; bmgt198716@gmail.com

[3] Department of Chemistry, Faculty of Science, University of Maroua, Maroua P.O. Box 55, Cameroon; atangana_albert@yahoo.fr

[4] Department of Chemistry, Faculty of Science, University of Dschang, Dschang P.O. Box 67, Cameroon;

[5] Department of Chemistry, Inorganic and Structural Chemistry, Bielefeld University, D-33501 Bielefeld, Germany; georg.stammler@uni-bielefeld.de

[6] Department of Chemistry, Higher Teacher Training College, University of Yaoundé 1 1, P.O. Box 47, Yaoundé, Cameroon; lentabruno@yahoo.fr

[7] Department of Chemistry, Faculty of Science, University of Douala, Douala P.O. Box 24157, Cameroon;

* Correspondence: ghis152001@gmail.com (G.W.F.); ngeufa@yahoo.fr (E.N.H.); norbert.sewald@uni-bielefeld.de (N.S.)

Citation: Noulala, C.G.T.; Ouete, J.L.N.; Atangana, A.F.; Mbahbou, G.T.B.; Fotso, G.W.; Stammler, H.-G.; Lenta, B.N.; Happi, E.N.; Sewald, N.; Ngadjui, B.T. Soyauxinine, a New Indolopyridoquinazoline Alkaloid from the Stem Bark of Araliopsis soyauxii Engl. (Rutaceae). *Molecules* **2022**, *27*, 1104. https://doi.org/10.3390/molecules27031104

Academic Editors: Weishuo Fang, Yu Zhang and Ana Estévez-Braun

Received: 27 November 2021
Accepted: 2 February 2022
Published: 7 February 2022

Publisher's Note: MDPI stays neutral with regard to jurisdictional claims in published maps and institutional affiliations.

Copyright: © 2022 by the authors. Submitted for possible open access publication under the terms and conditions of the Creative Commons Attribution (CC BY) license (https://creativecommons.org/licenses/by/4.0/).

Abstract: The chemical investigation of the total alkaloid extract (TAE) of the stem bark of *Araliopsis soyauxii* (Rutaceae) afforded an unreported indolopyridoquinazoline (**1**) along with nine previously known alkaloids (**2–10**). In addition, six semi-synthetic derivatives (**3a–c, 4b, 5a** and **6a**) were prepared by allylation and acetonidation of soyauxinium nitrate (**5**), edulinine (**3**), ribalinine (**4**) and arborinine (**6**). The structures and spectroscopic data of five of them are reported herein for the first time. The suggested mechanism for the formation of the new *N*-allylindolopyridoquinazoline (**5a**) is presented. The structures of natural and derived compounds were determined employing extensive NMR and MS techniques. The absolute configuration of stereogenic centers in compounds **2–4** were determined using NOESY technique and confirmed by the single-crystal X-ray diffraction (SC-XRD) technique. The use of SC-XRD further enabled us to carry out a structural revision of soyauxinium chloride recently isolated from the same plant to soyauxinium nitrate (**5**). The TAE, fractions, compounds (**1–7** and **9**), and semi-synthetic derivatives (**3a–c**, **4b**, **5a** and **6a**) were evaluated for their cytotoxic activity towards the cervix carcinoma cell line KB-3-1. No significant activity was recorded for most of the compounds except for **9**, which showed moderate activity against the tested cancer cell lines.

Keywords: *Araliopsis soyauxii*; Rutaceae; alkaloids; soyauxinine, cytotoxic activity

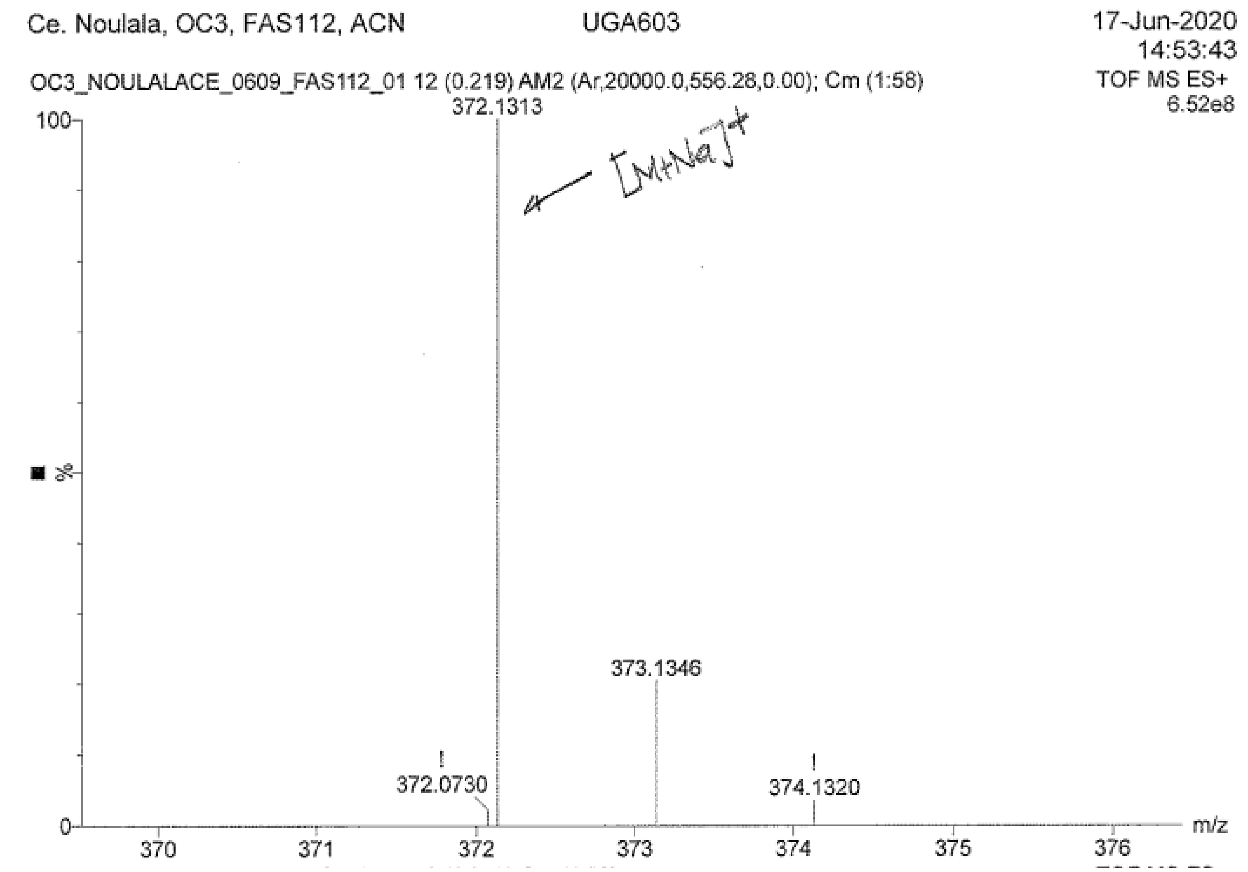

Figure S1: ESI-HR Mass spectrum of compound 1.

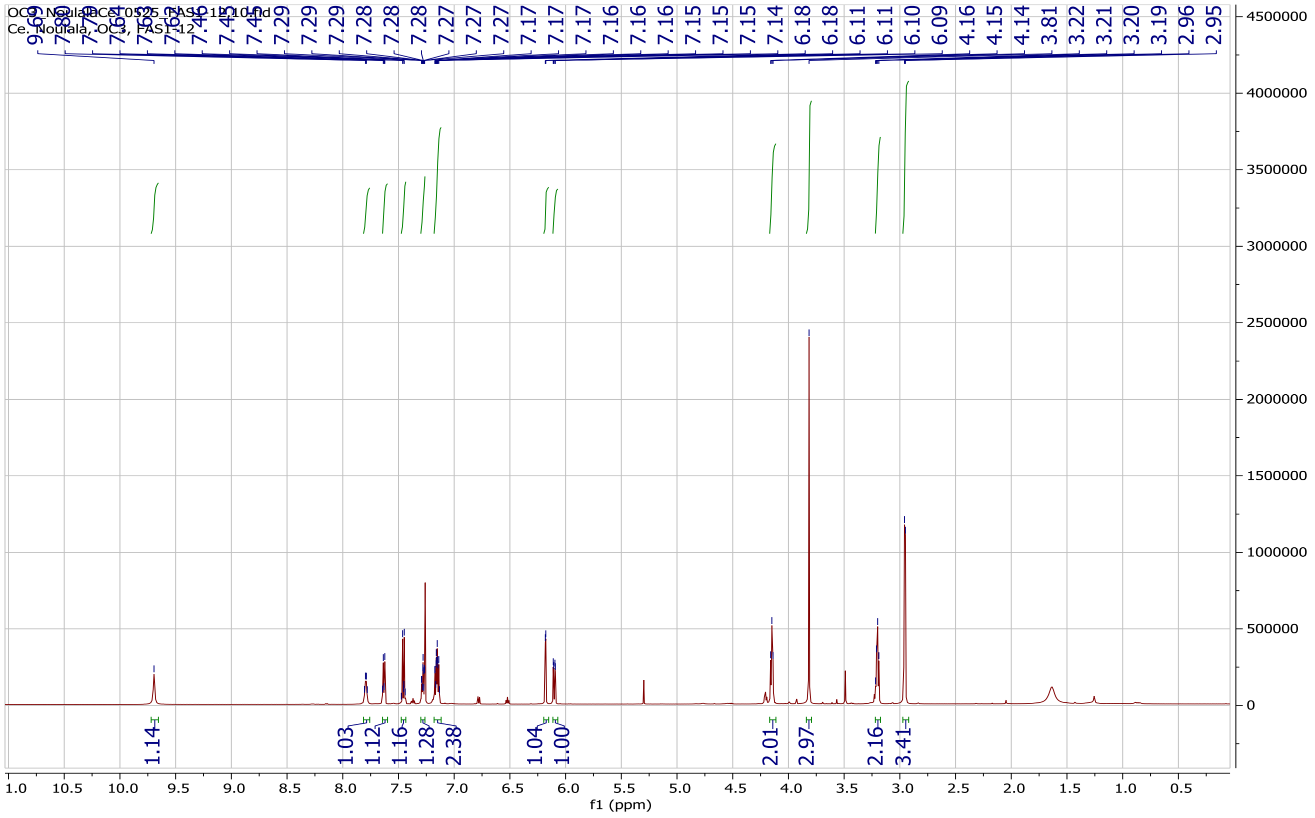

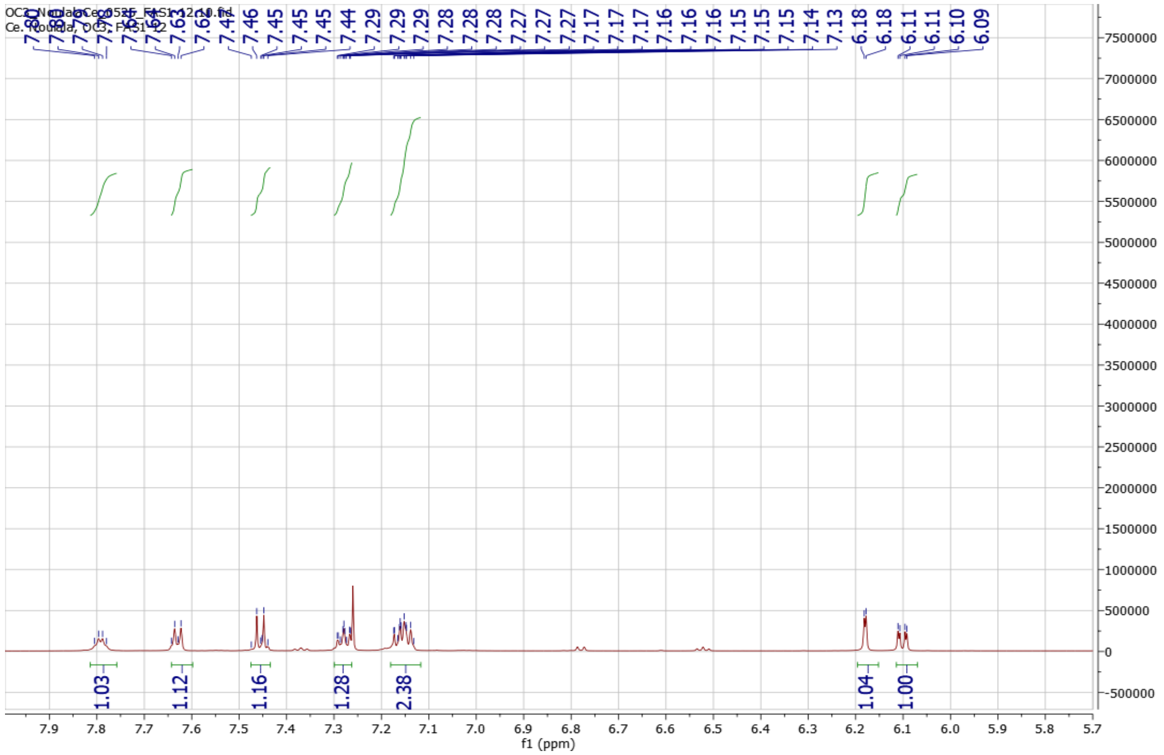

Figure S2: ^{1}H NMR (600 MHz, $CDCl_3$) spectra of compound 1.

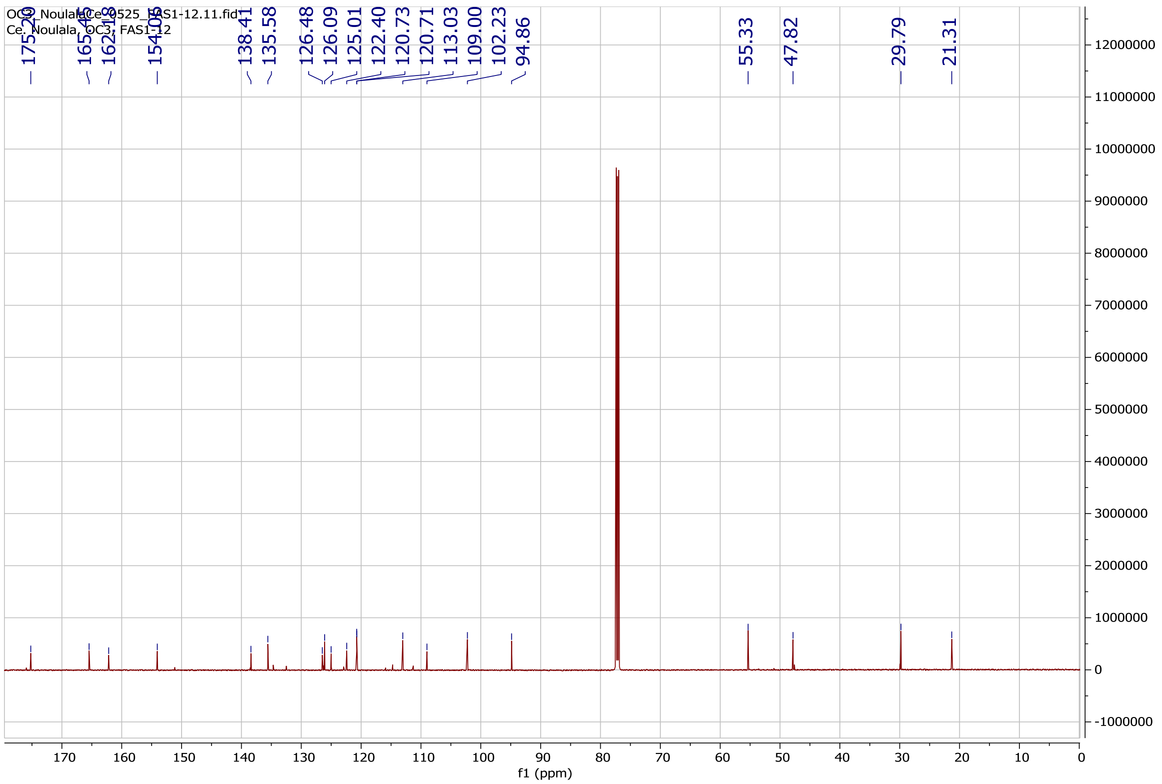

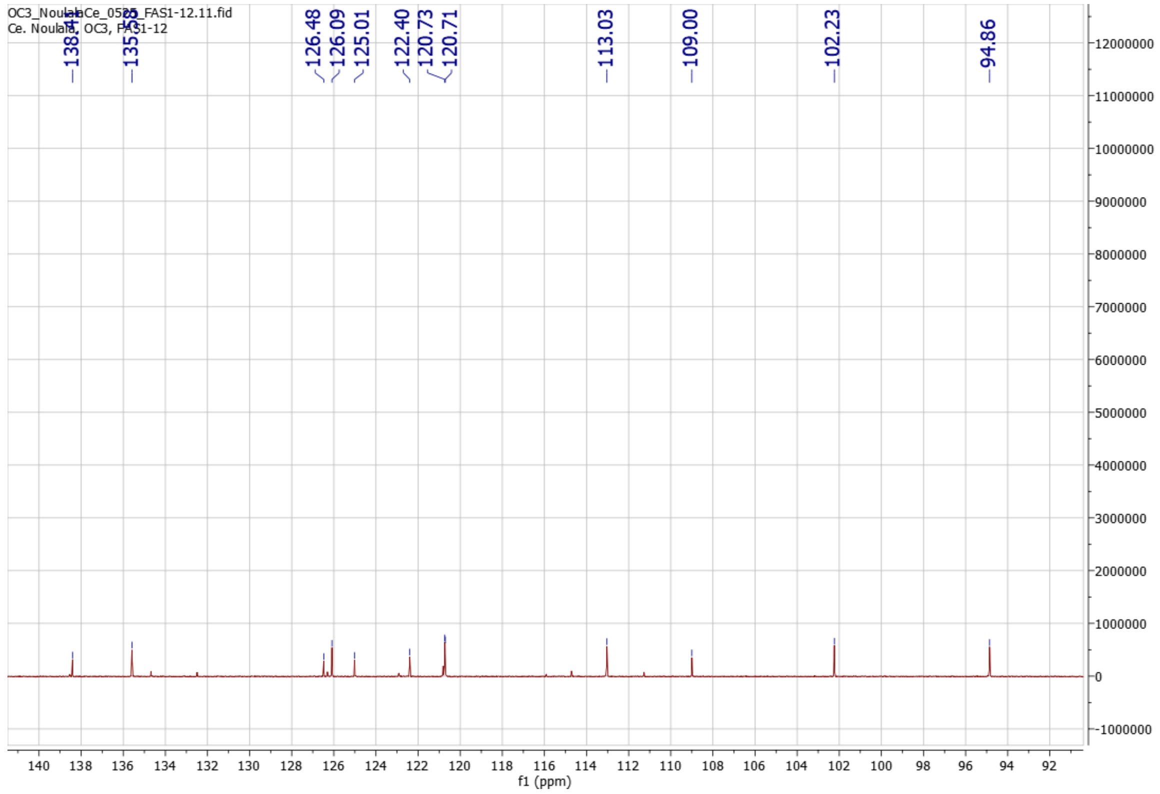

Figure S3: ^{13}C NMR (150 MHz, $CDCl_3$) spectra of compound 1.

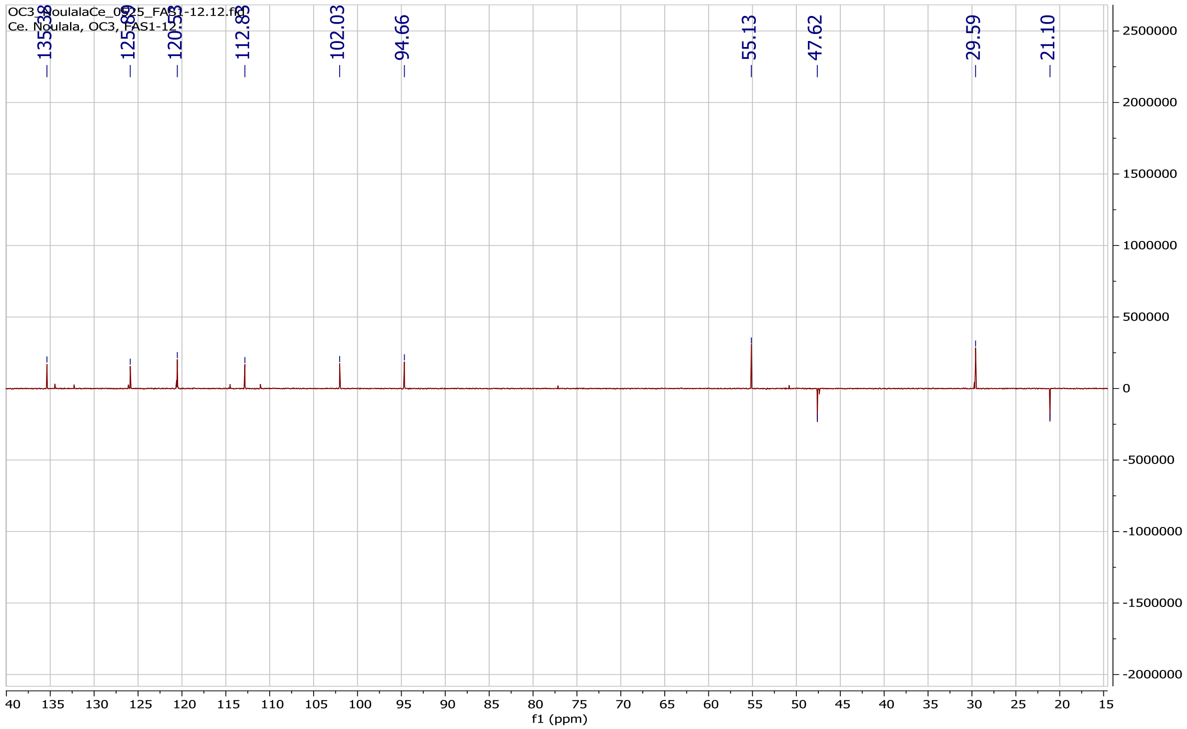

Figure S4: DEPT 135 (150 MHz, $CDCl_3$) spectra of compound 1.

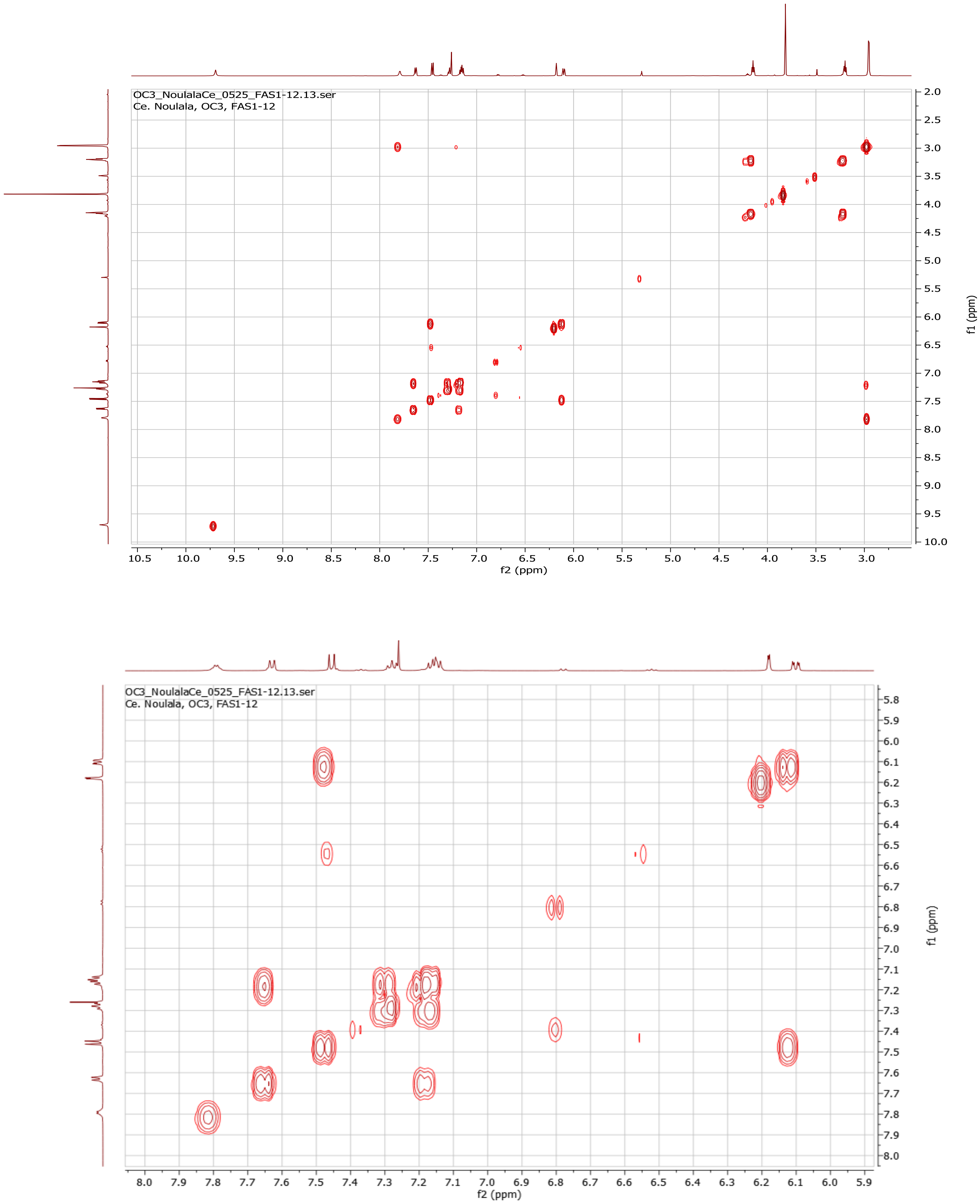

Figure S5: COSY spectra of compound 1.

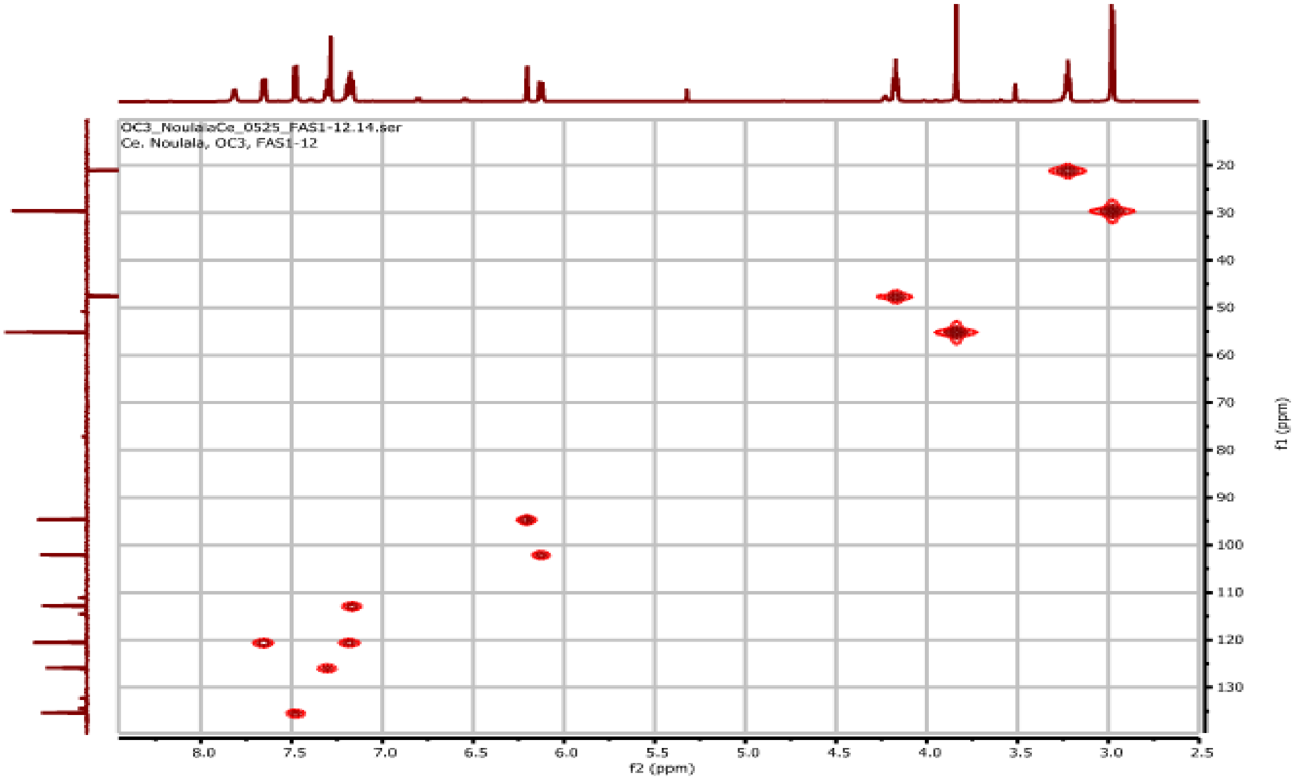

Figure S6: HSQC spectra of compound 1.

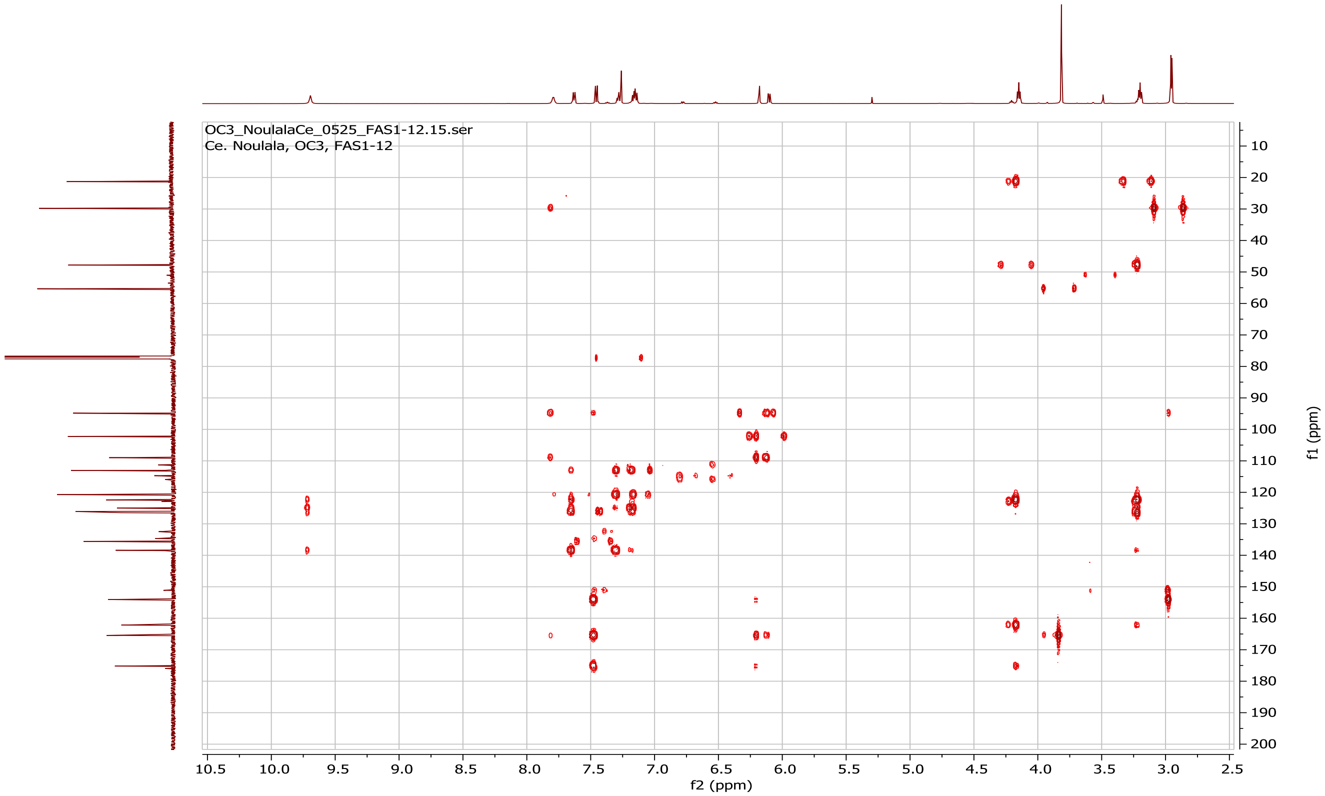

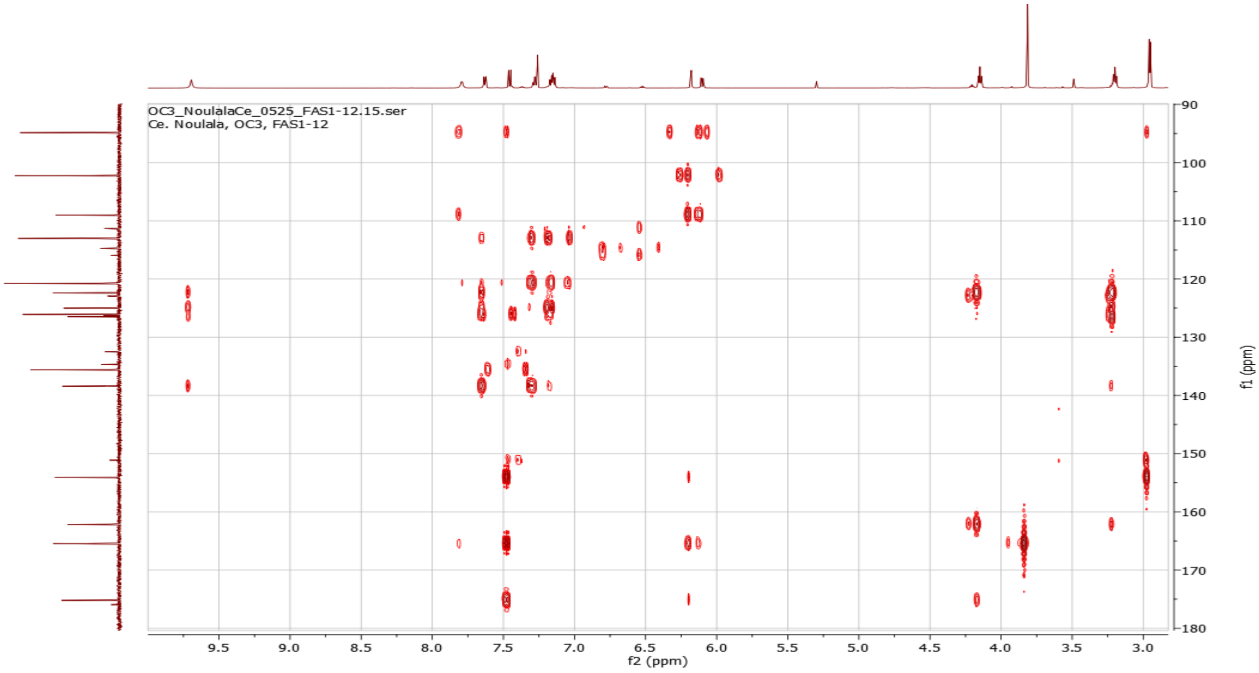

Figure S7: HMBC spectra spectra of compound 1.

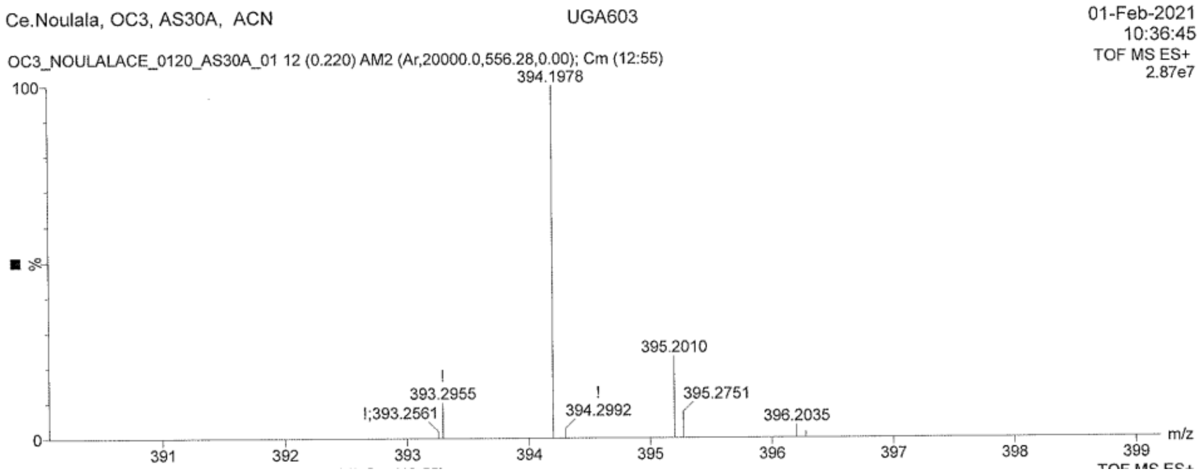

Figure S8: ESI-HR Mass spectrum of compound 3a.

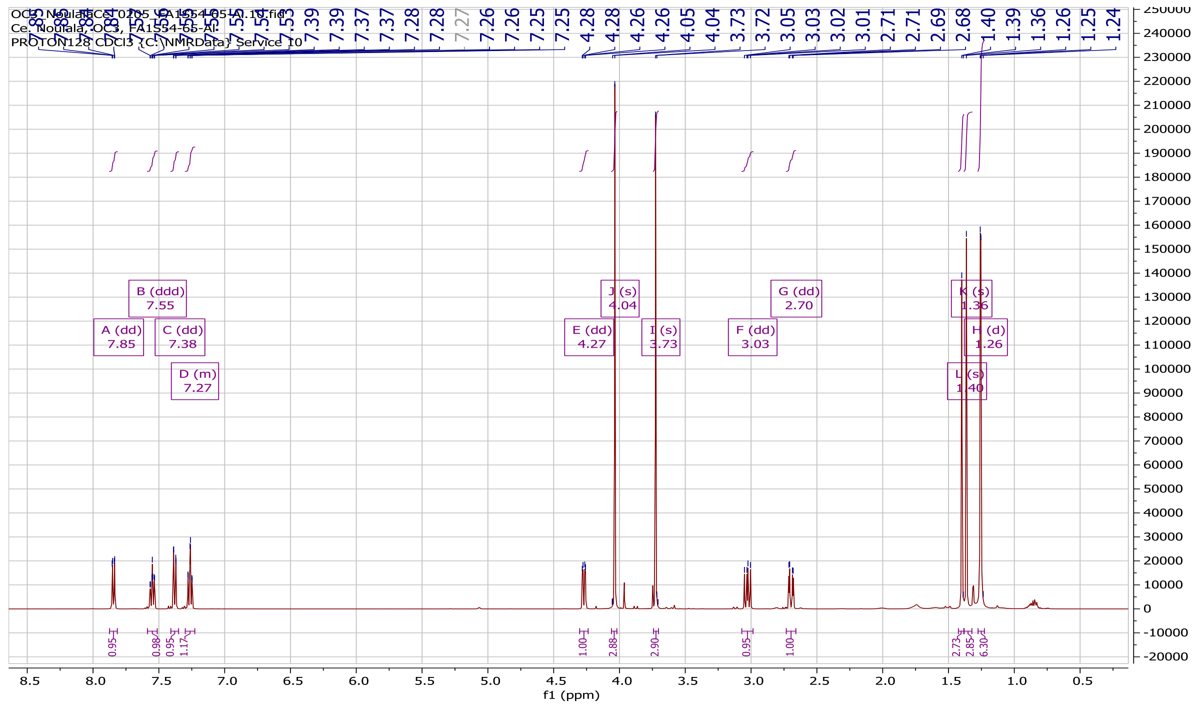

Figure S9: ^{1}H NMR (600 MHz, $CDCl_3$) spectrum of compound 3a.

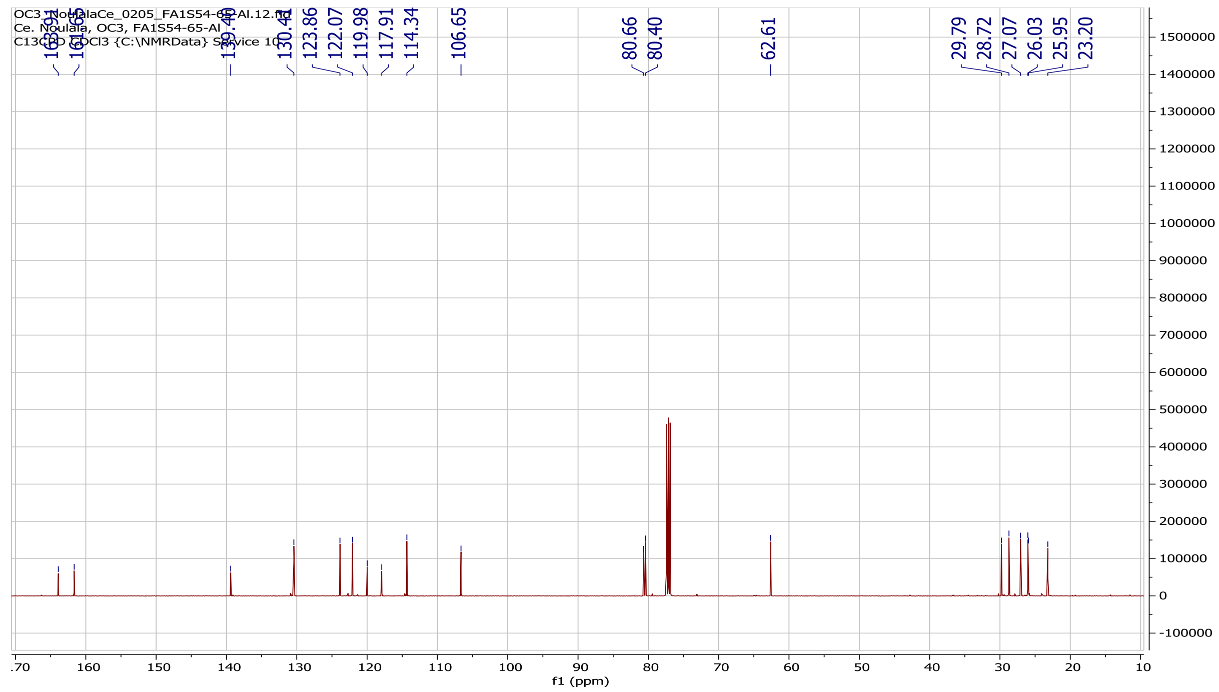

Figure S10: ^{13}C NMR (150 MHz, $CDCl_3$) spectrum of compound 3a.

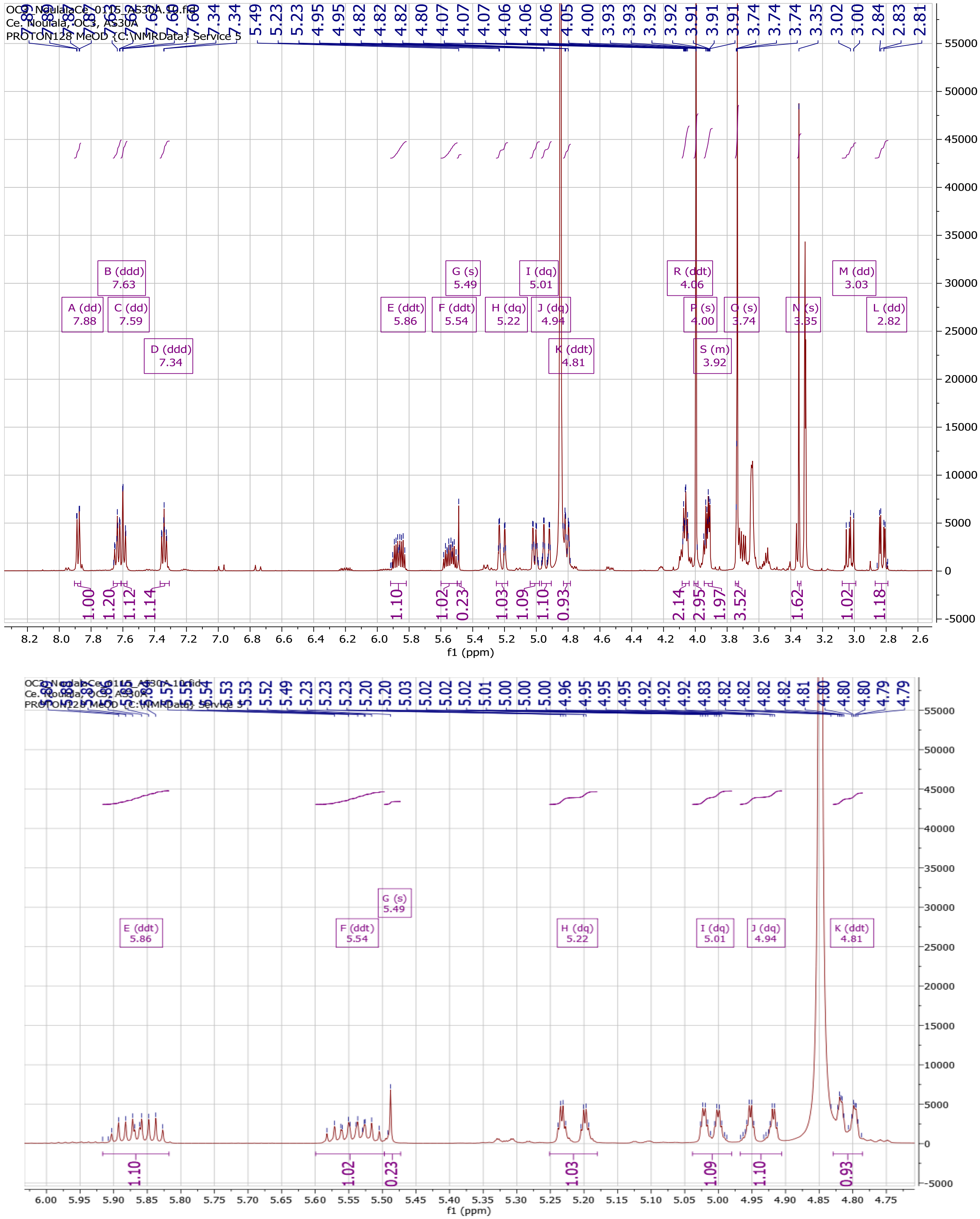

Figure S11: [1] H NMR (600 MHz, CD_3OD) spectra of compound 3a.

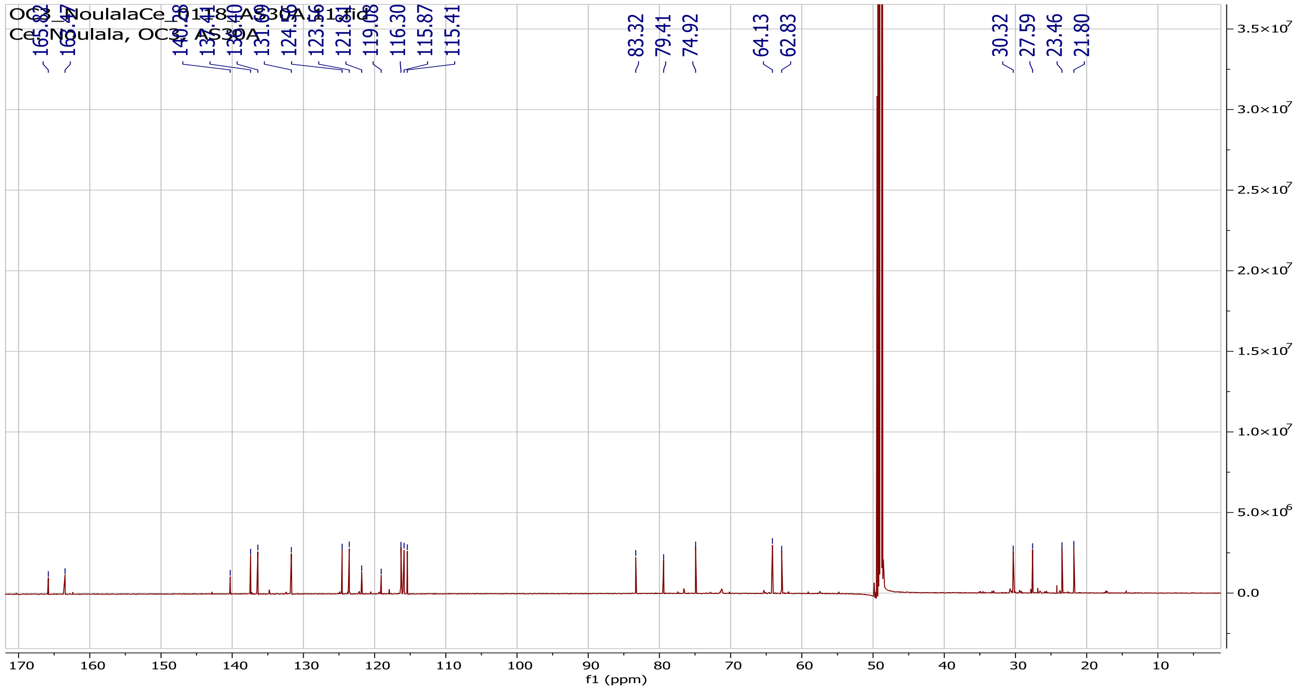

Figure S12: ^{13}CNMR (150 MHz, CD_3OD) spectra of compound 3a.

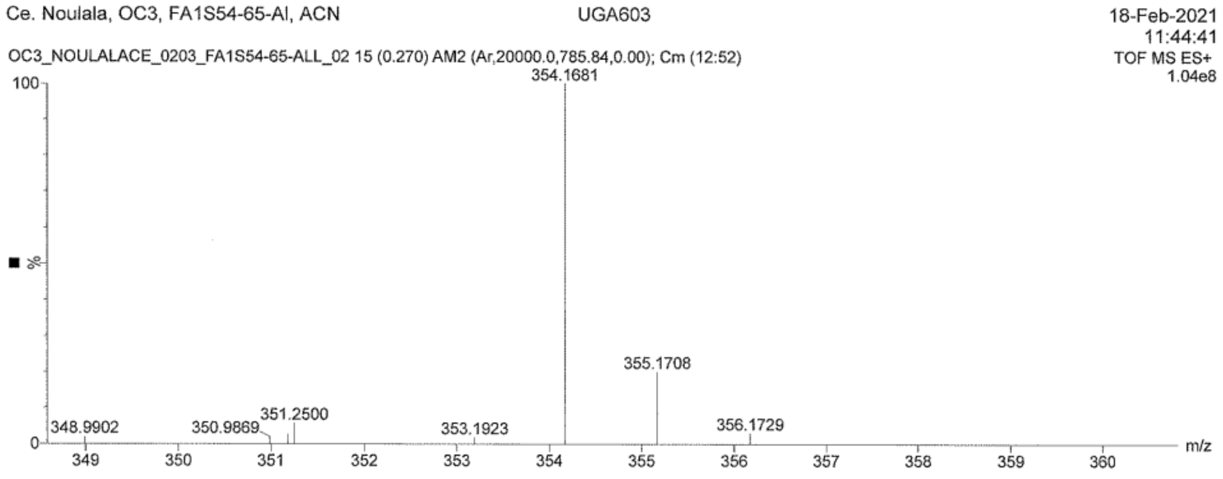

Figure S13: ESI-HR Mass spectrum of compound 3b.

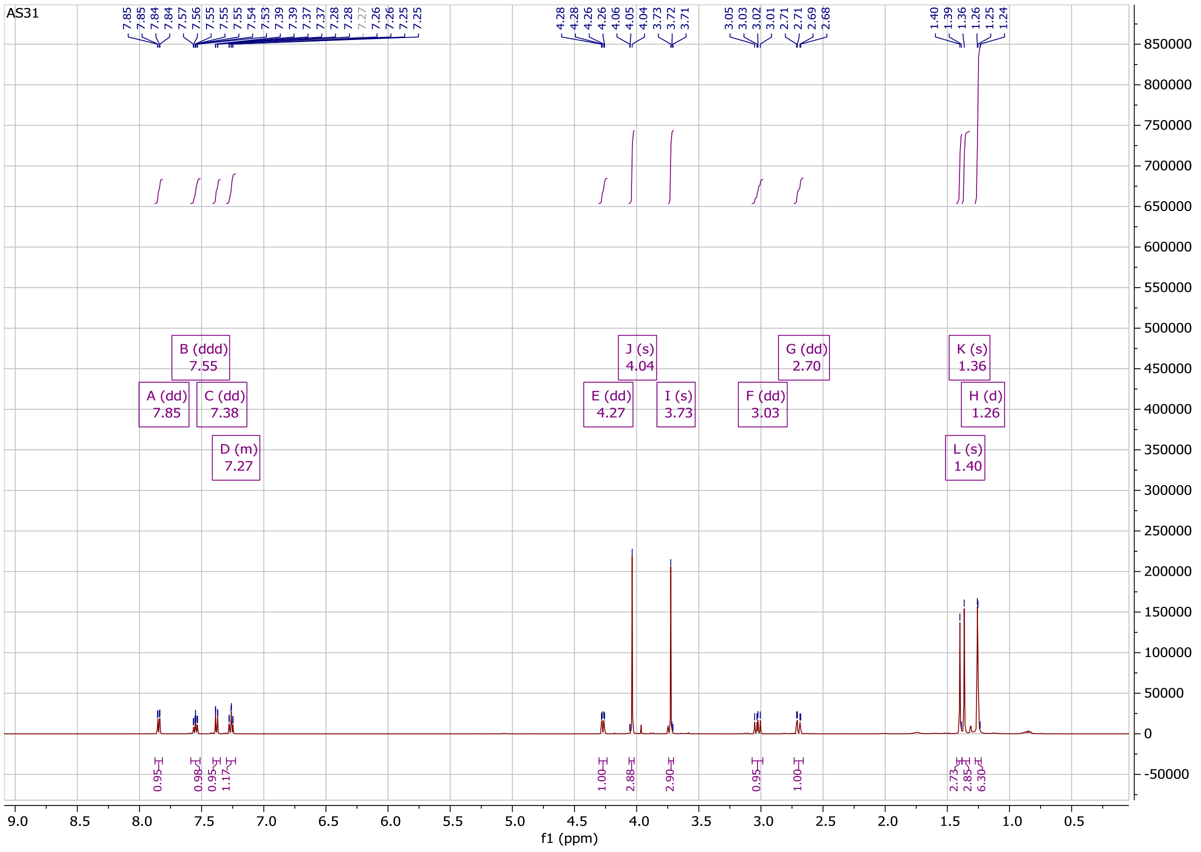

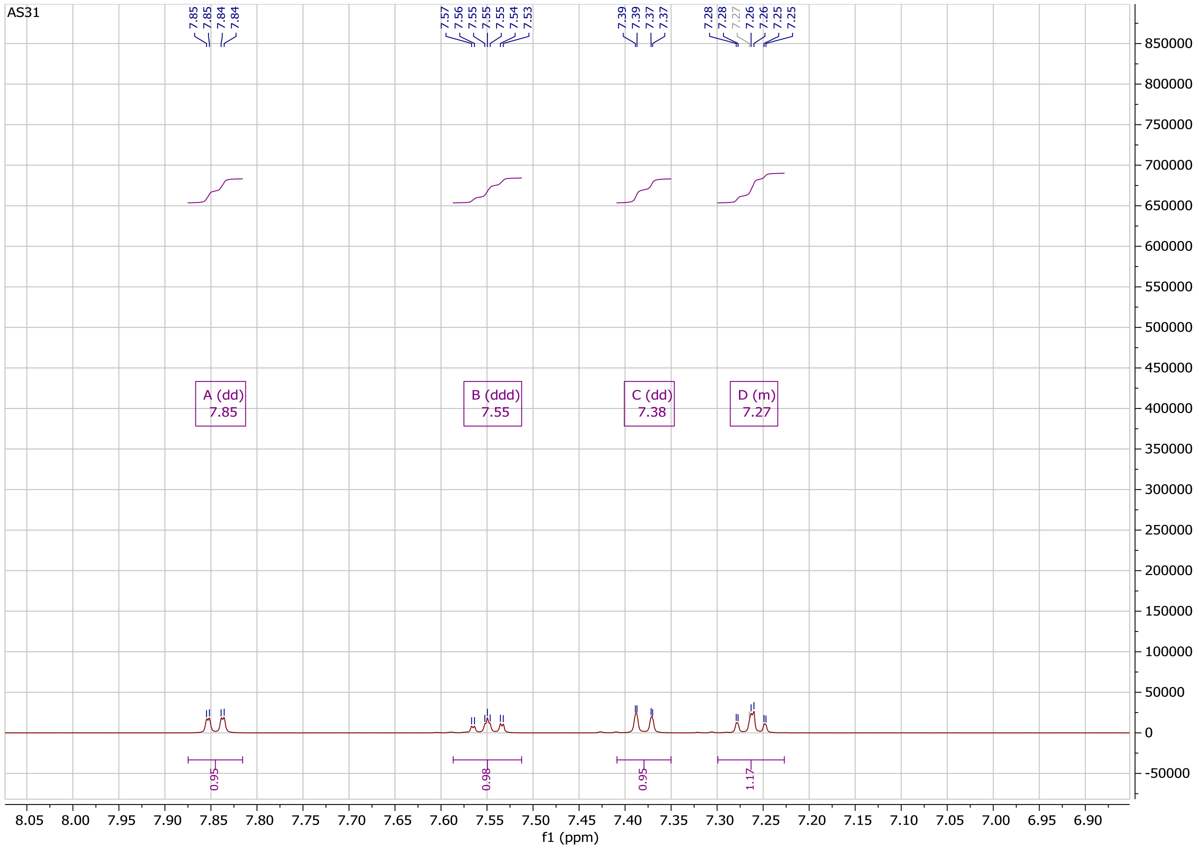

Figure S14 : [1] H NMR (500 MHz, $CDCl_3$) spectra of compound 3b.

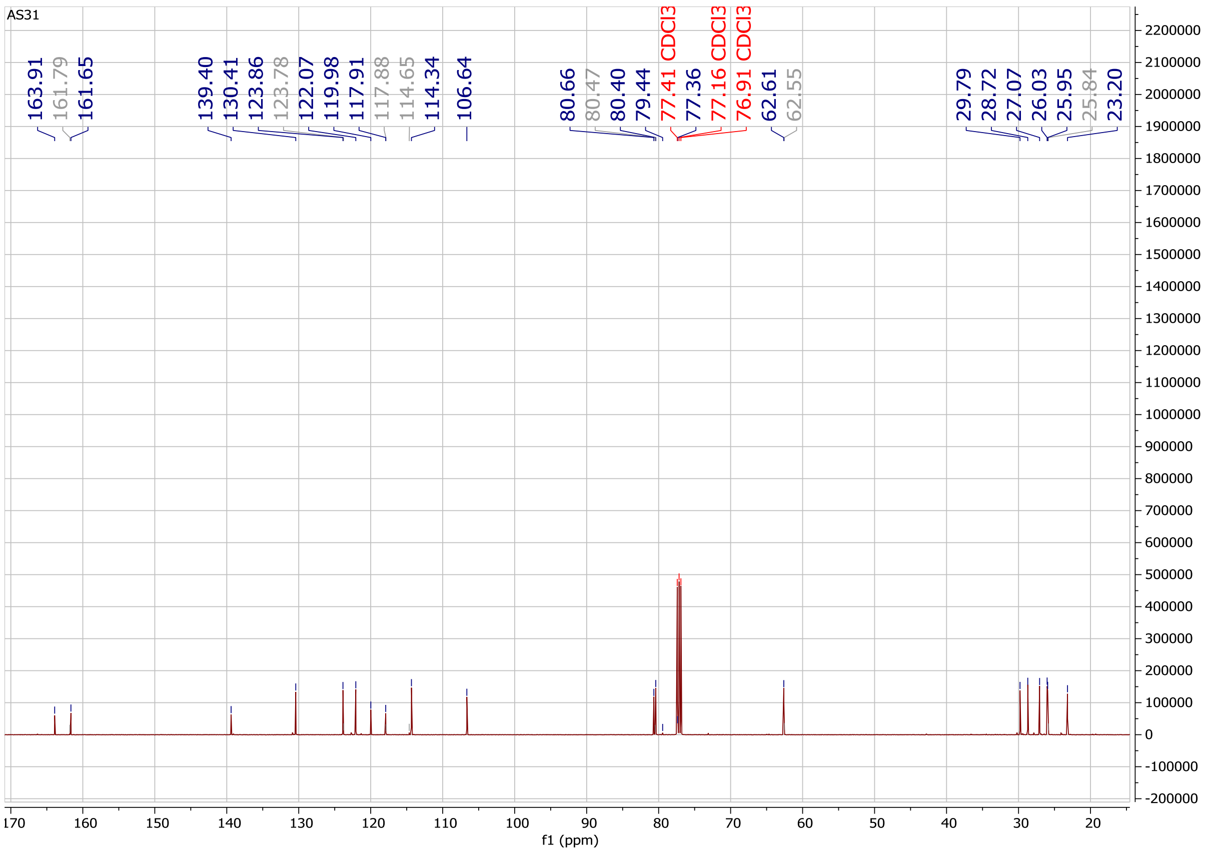

Figure S15: ^{13}C NMR (150 MHz, $CDCl_3$) spectra of compound 3b.

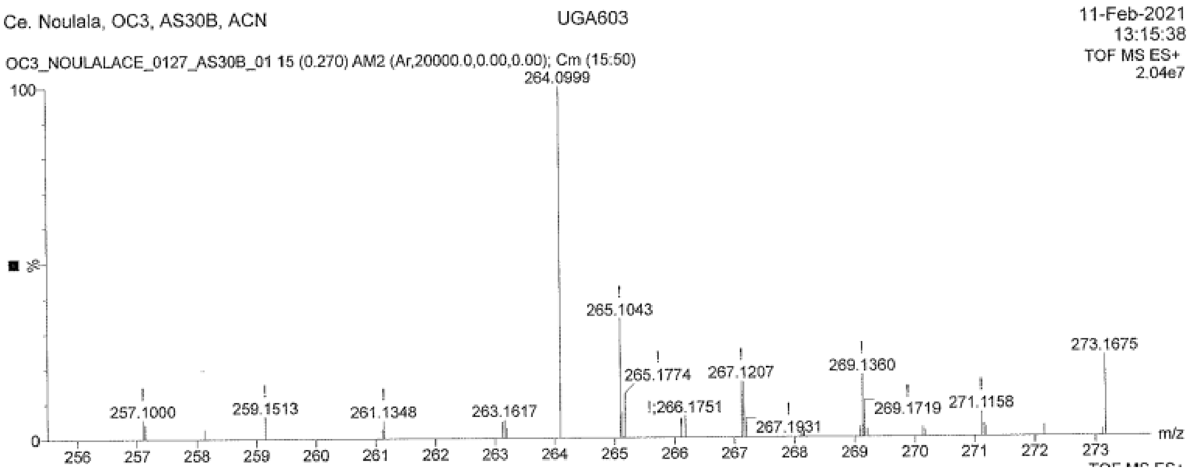

Figure S16: ESI-MS of compound 3c

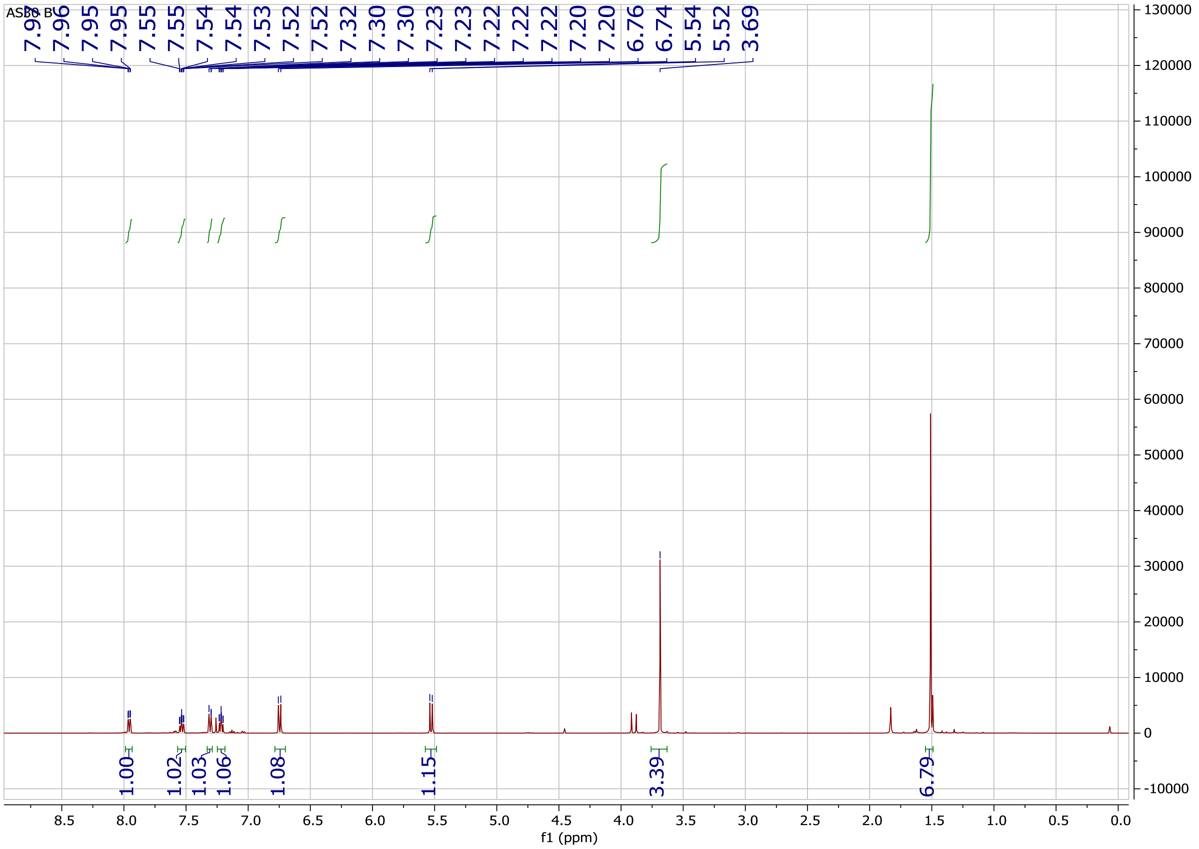

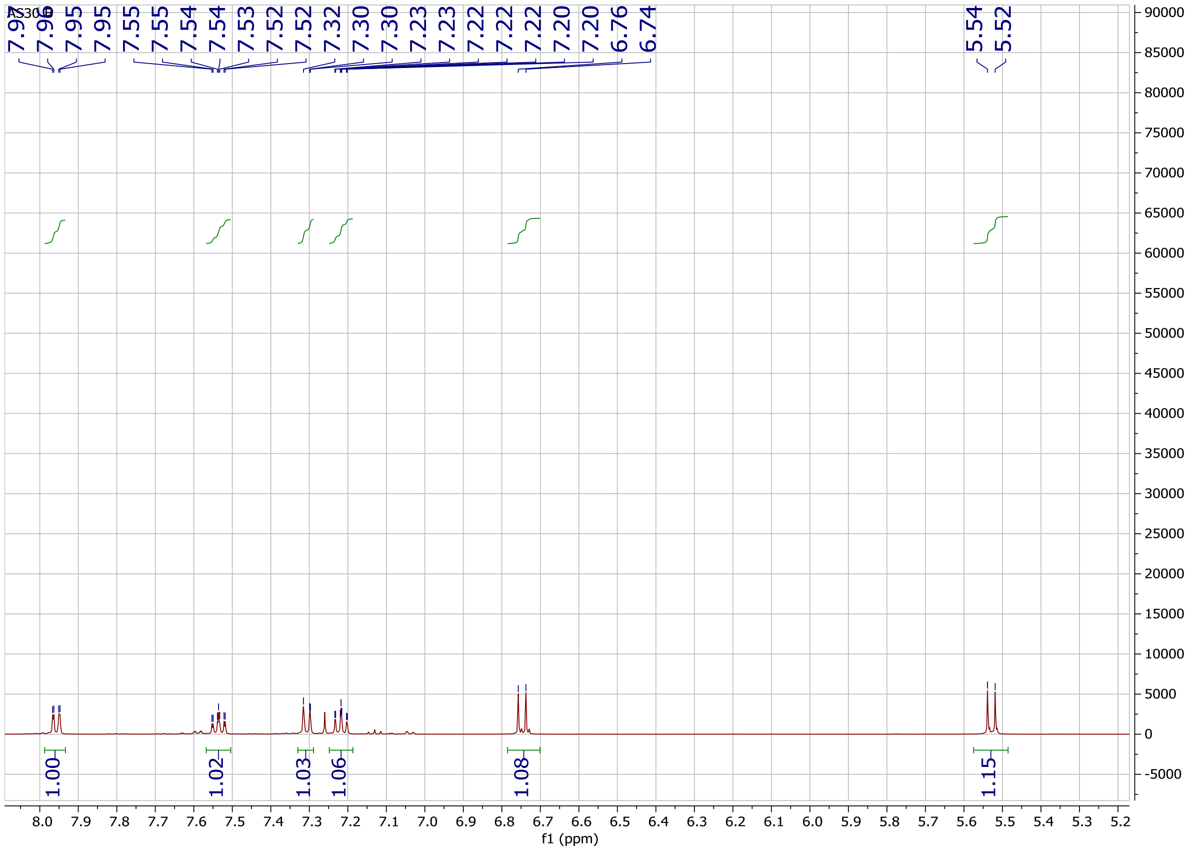

Figure S17: [1] H NMR (500 MHz, $CDCl_3$) spectra of compound 3c.

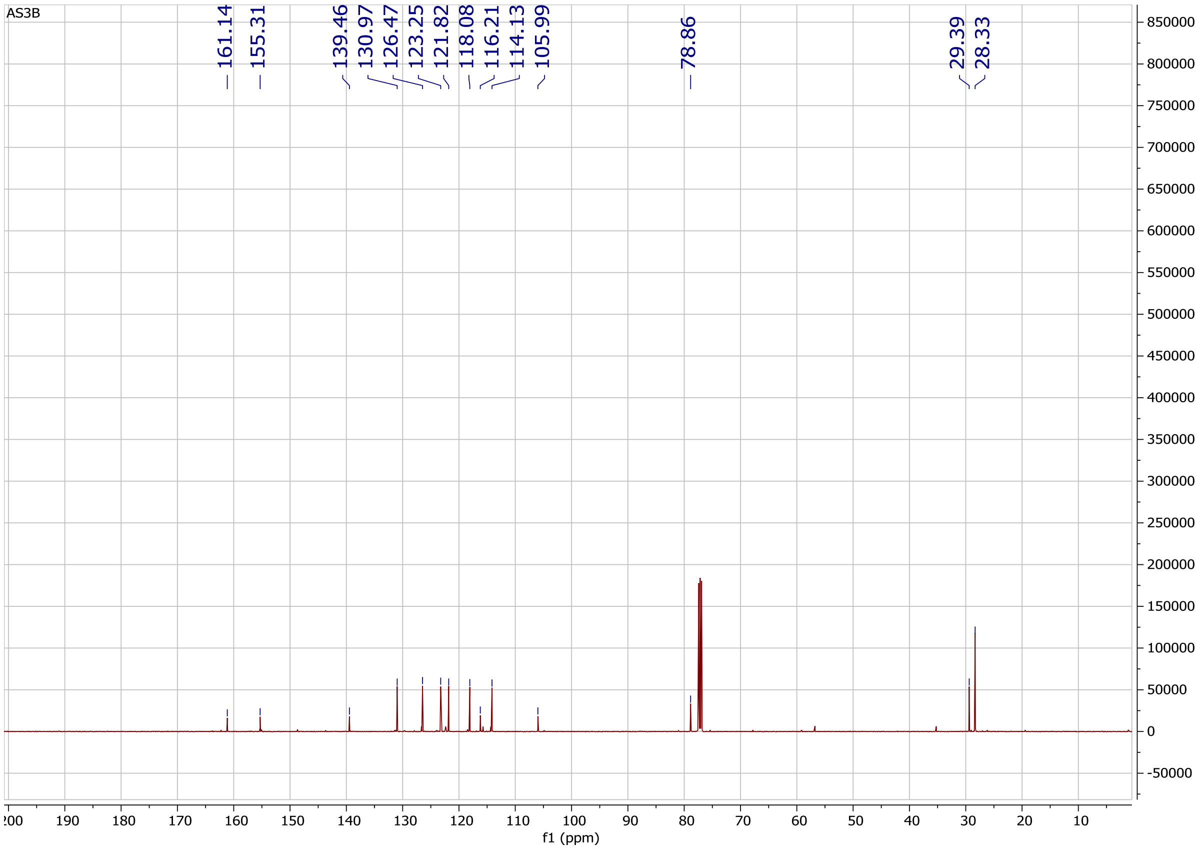

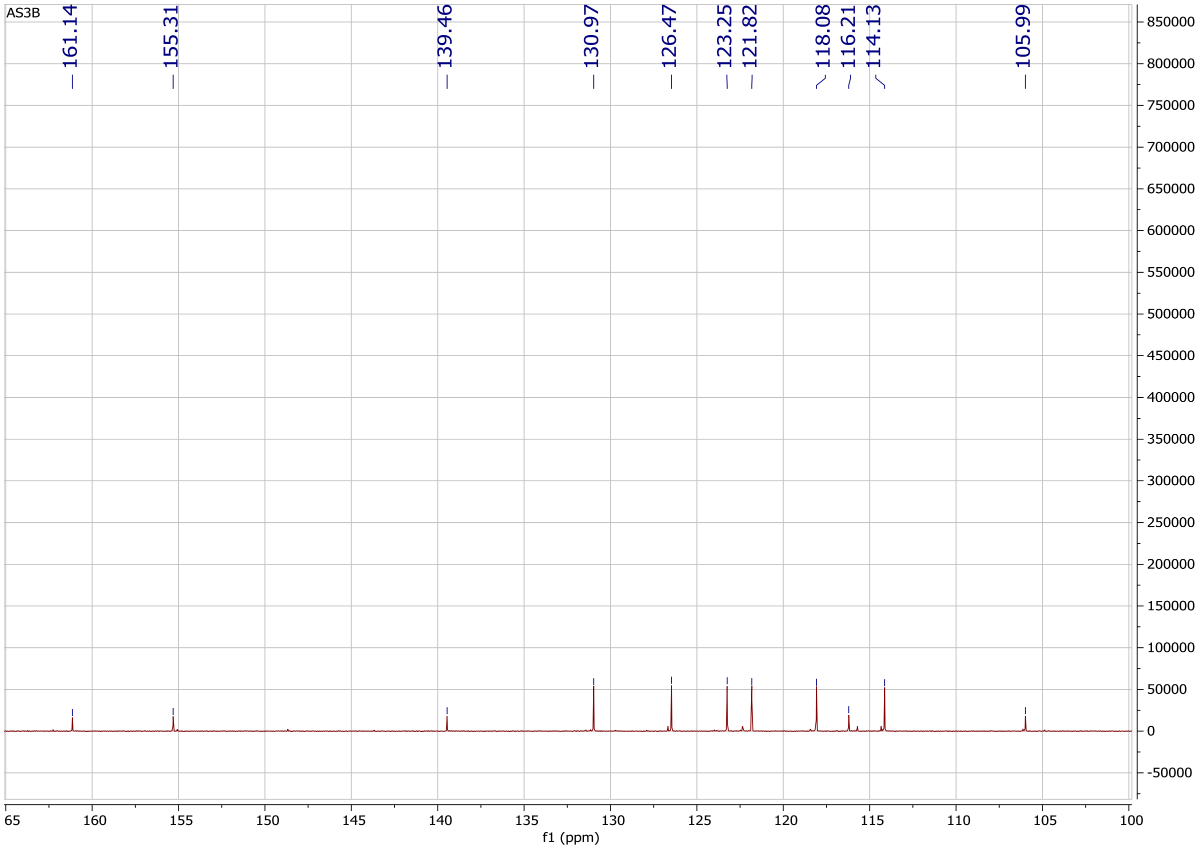

Figure S18: ^{13}C NMR (125 MHz, $CDCl_3$) spectra of compound 3c.

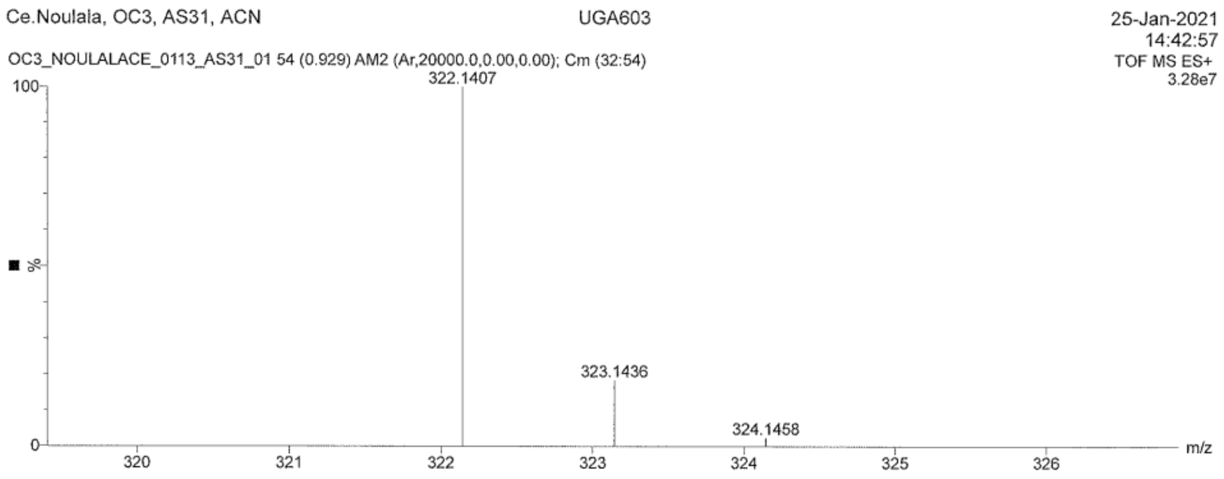

Figure S19: ESI-HR Mass spectrum of compound 4b.

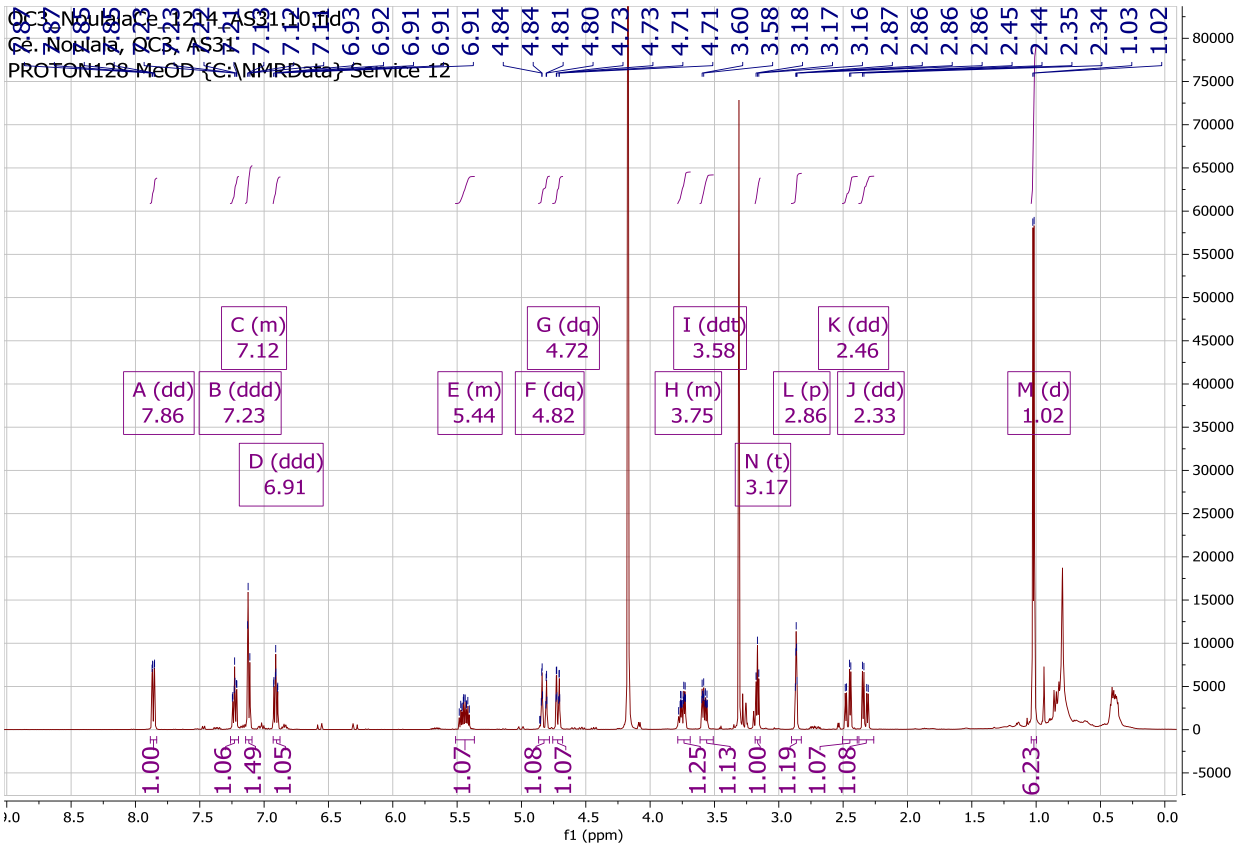

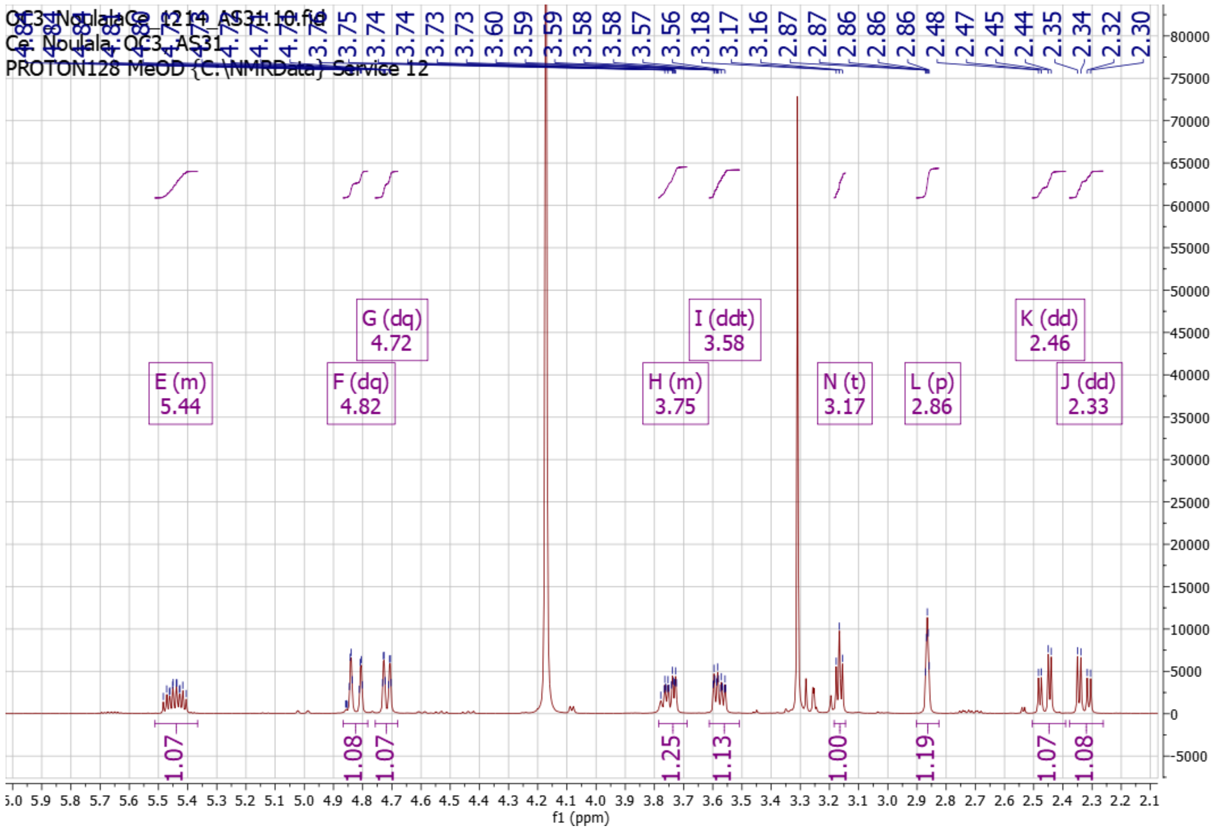

Figure S20: ^{1}H NMR (600 MHz, $CDCl_3$,) spectra of compound 4b.

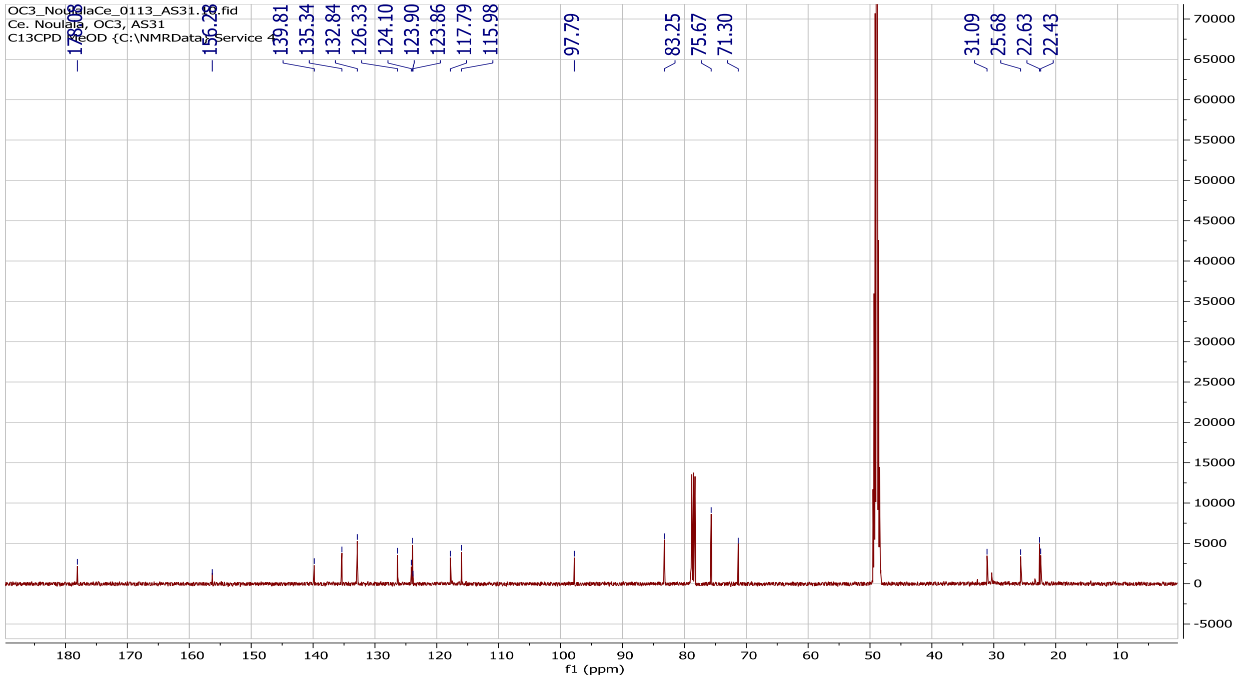

Figure S21: ^{13}C-NMR (150 MHz, $CDCl_3$) spectra of compound 4b.

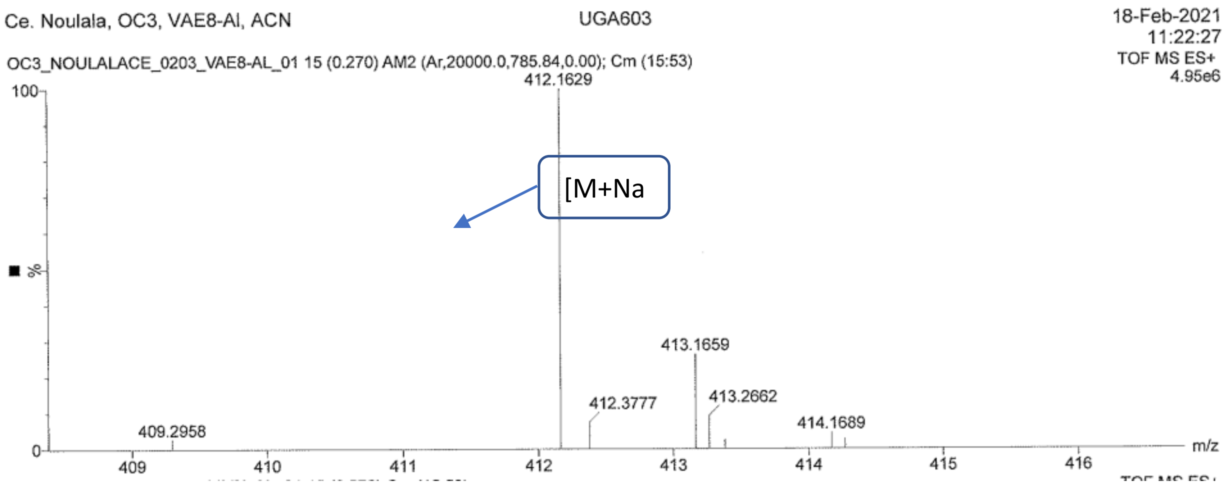

Figure S22: ESI-HR Mass spectrum of compound 5a.

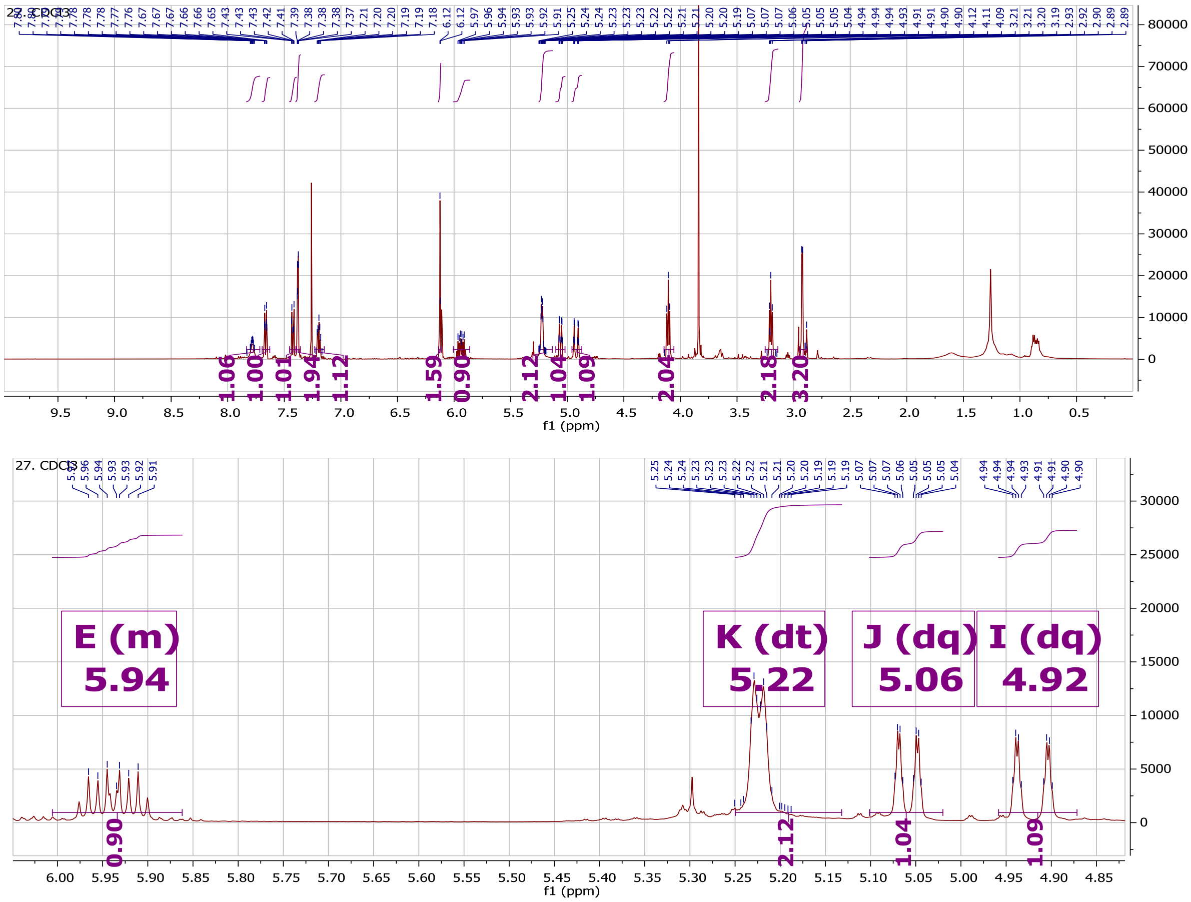

Figure S23: ^{1}H NMR (600 MHz, $CDCl_3$) spectra of compound 5a.

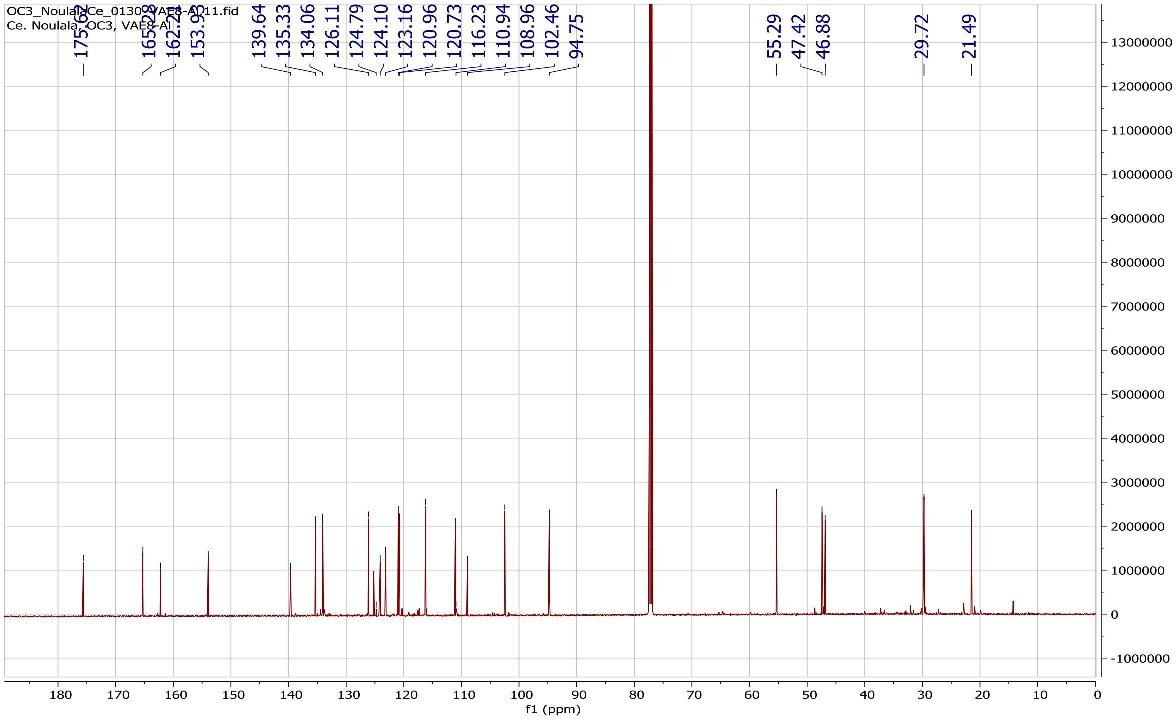

Figure S24: ^{13}CNMR (150 MHz, $CDCl_3$) spectra of compound 5a.

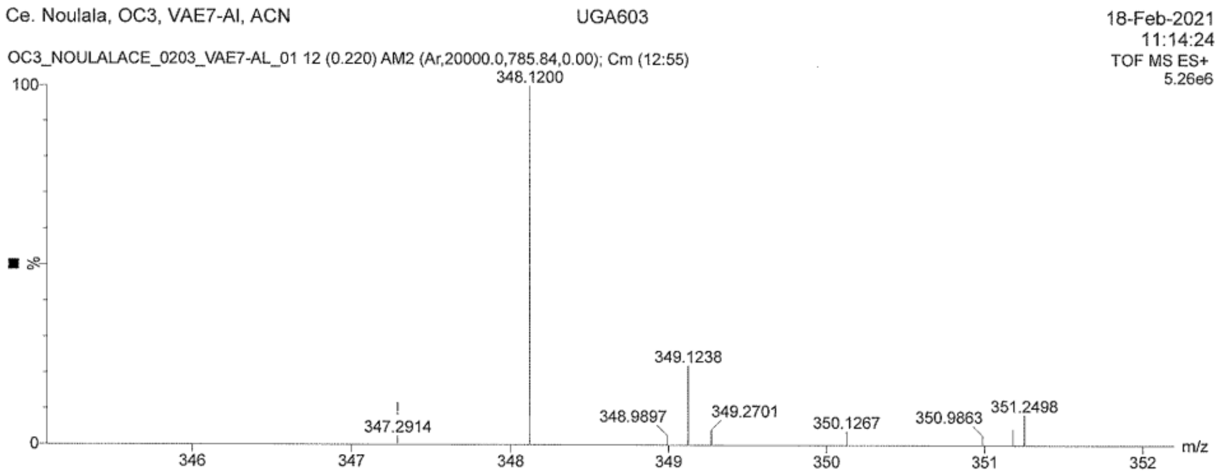

Figure S25: ESI-HR Mass spectrum of compound 6a.

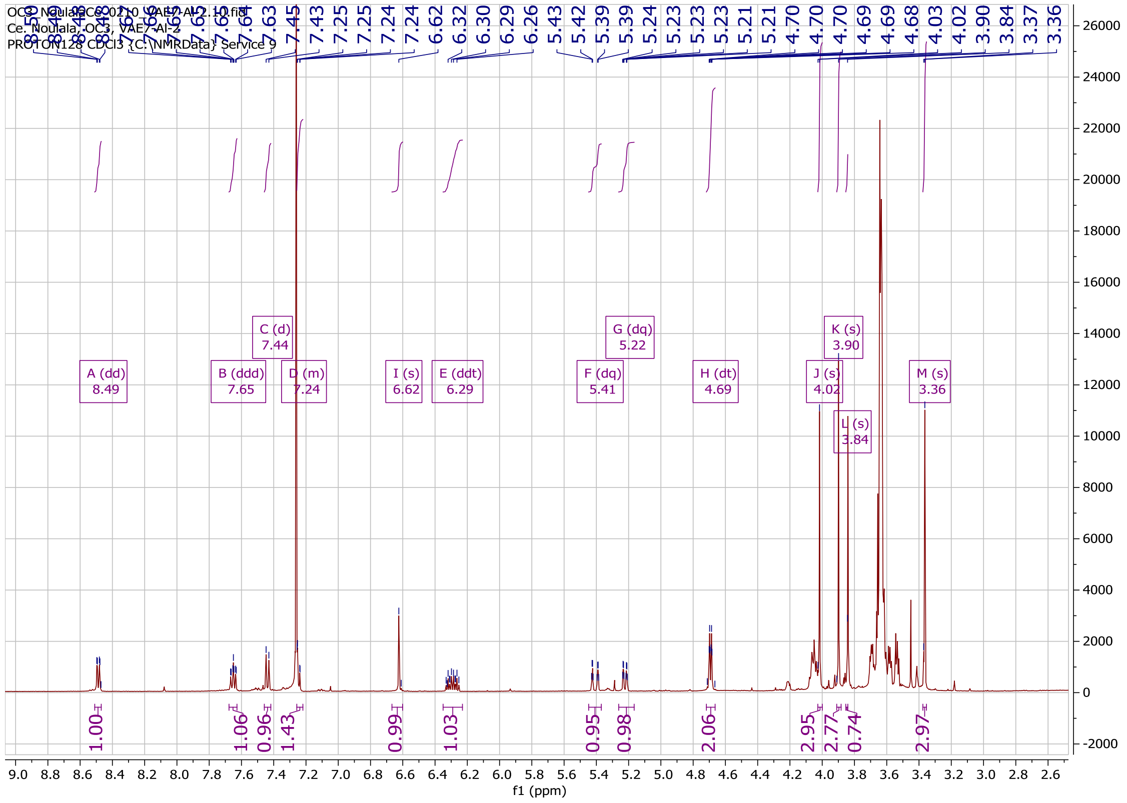

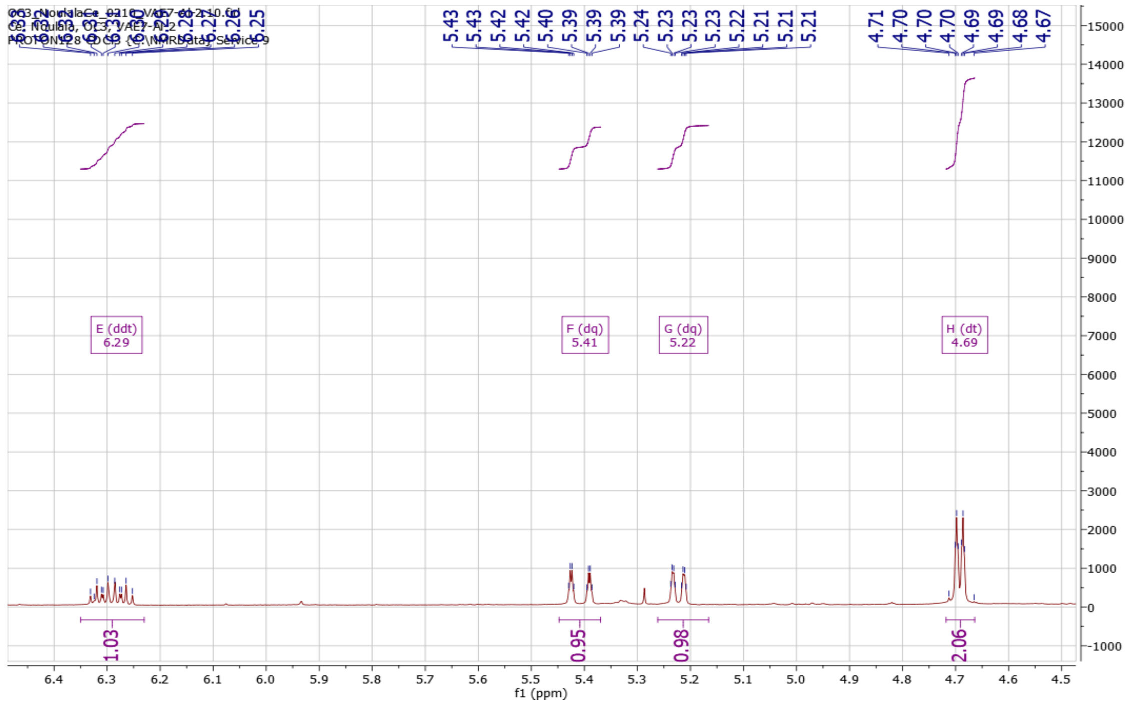

Figure S26: ^{1}H NMR (600 MHz, $CDCl_3$,) spectra of compound 6a.

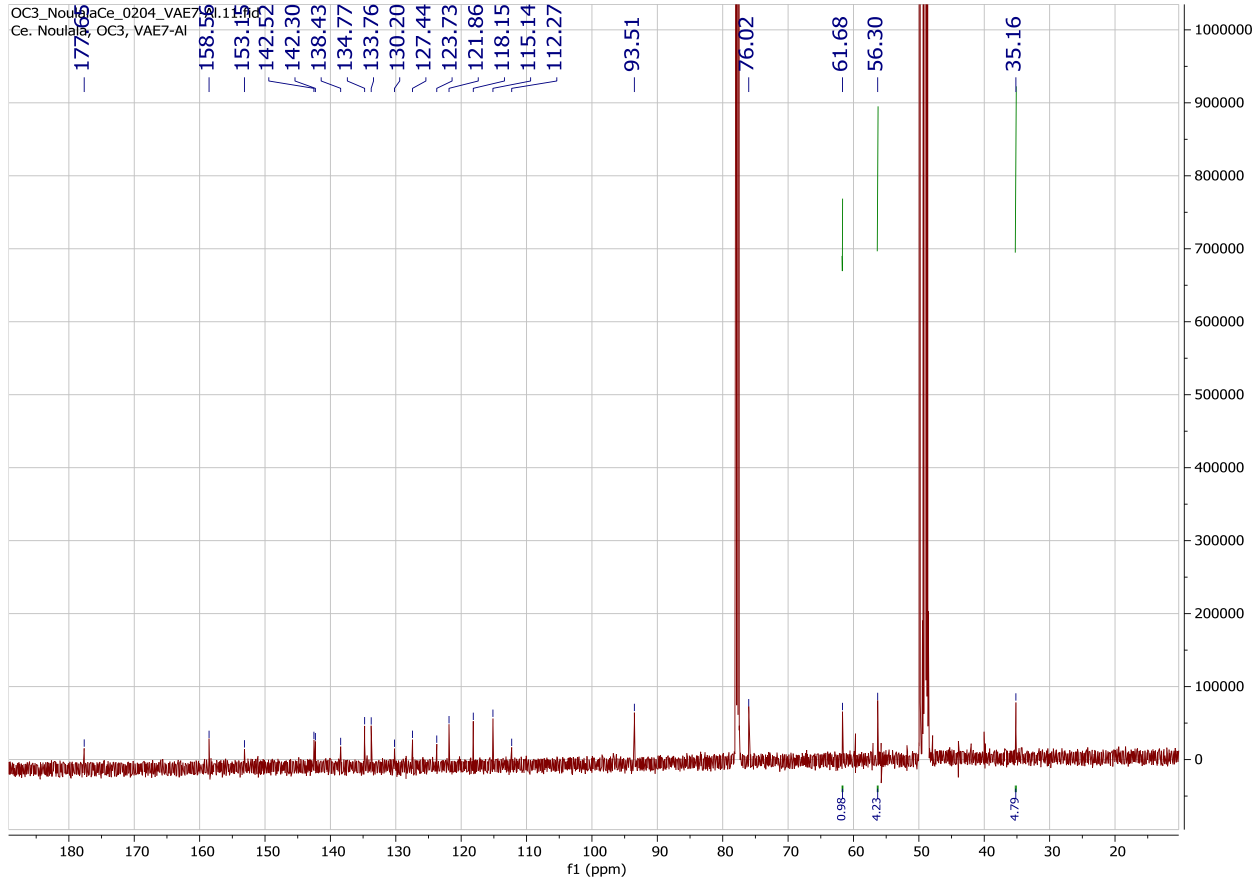

Figure S27: ^{13}CNMR (150 MHz, $CDCl_3$) spectra of compound 6a.

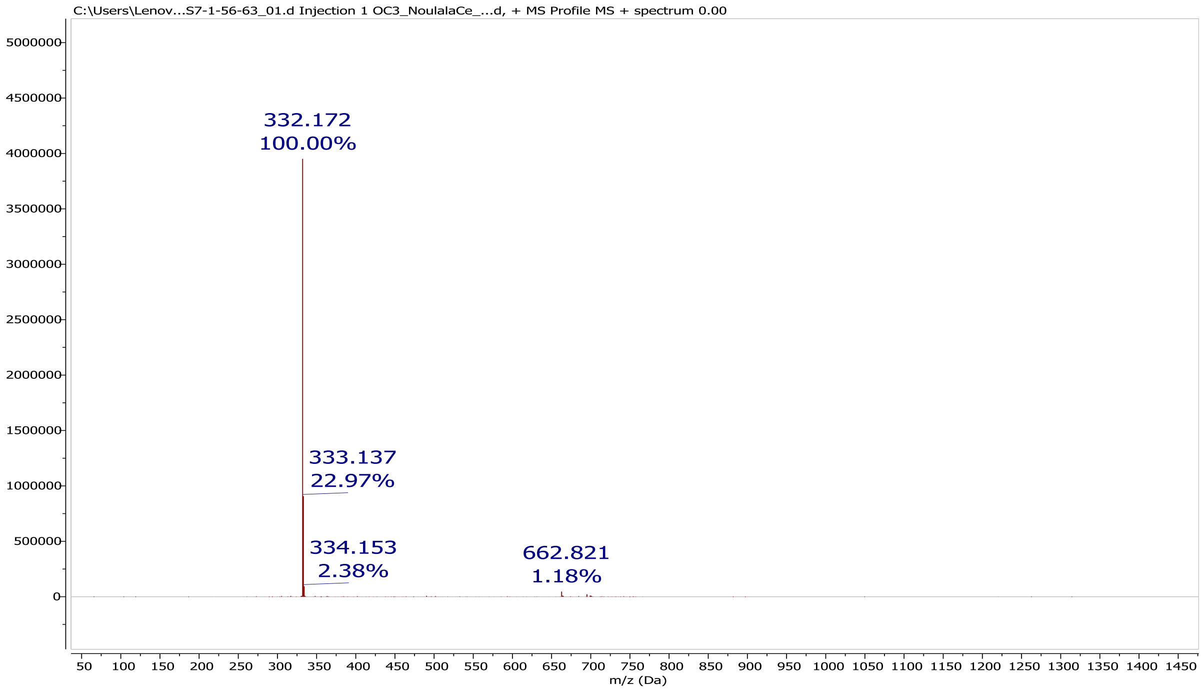

Figure S28: MS spectrum of compound 5.

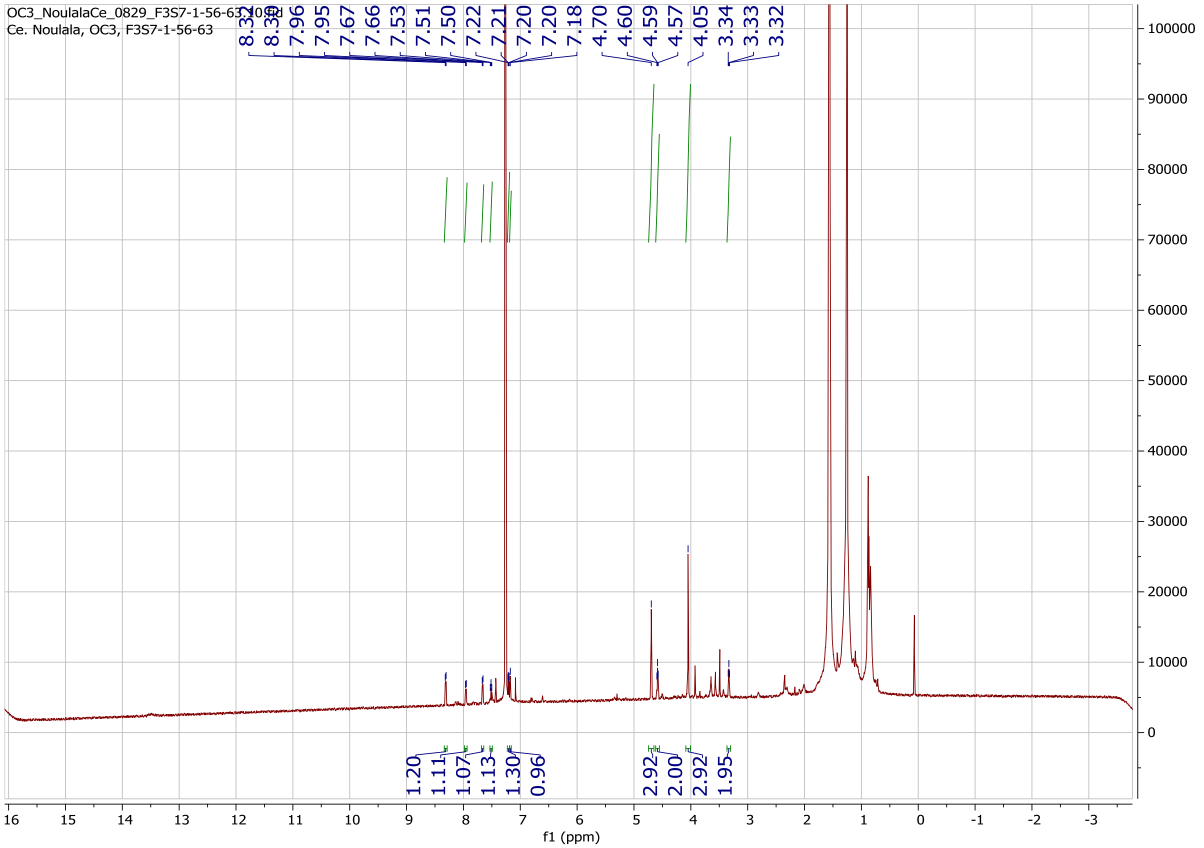

Figure S29: ¹H NMR (600 MHz, CDCl₃,) spectra of compound 5.

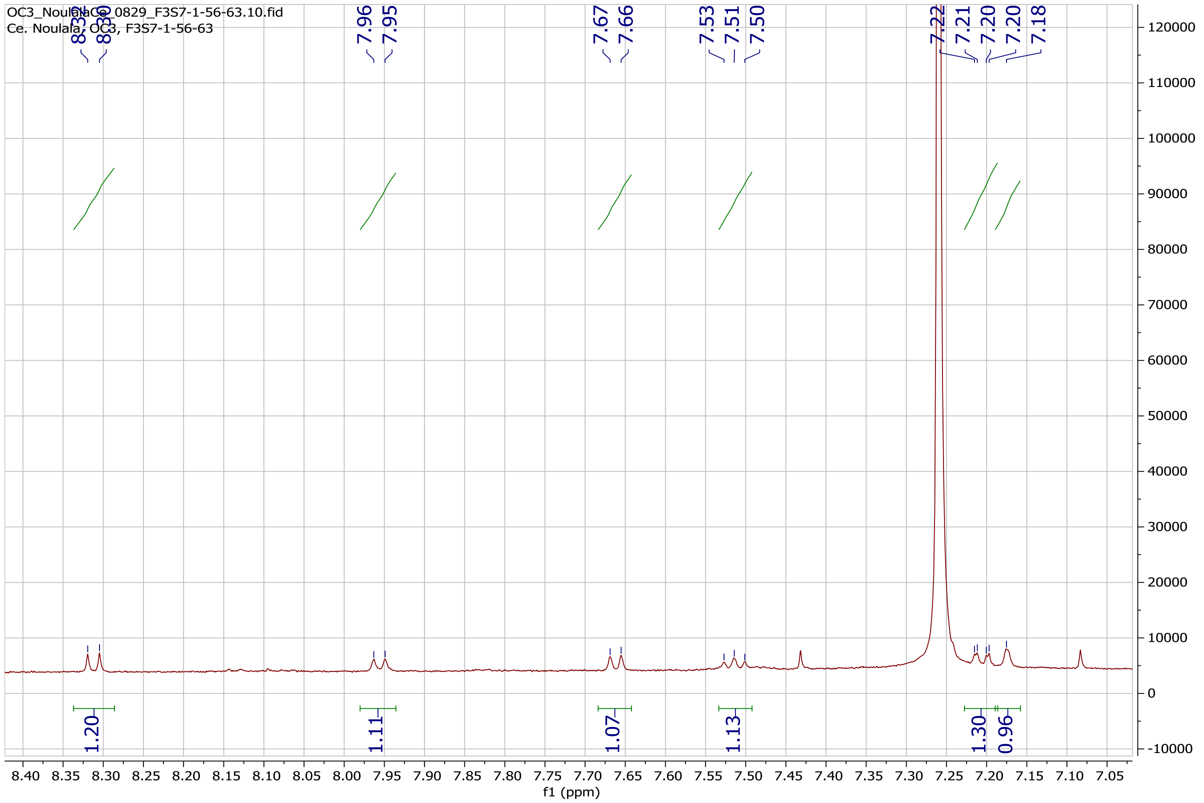

Figure S29a: ¹H NMR (600 MHz, CDCl₃,) spectra of compound 5(aromatic region).

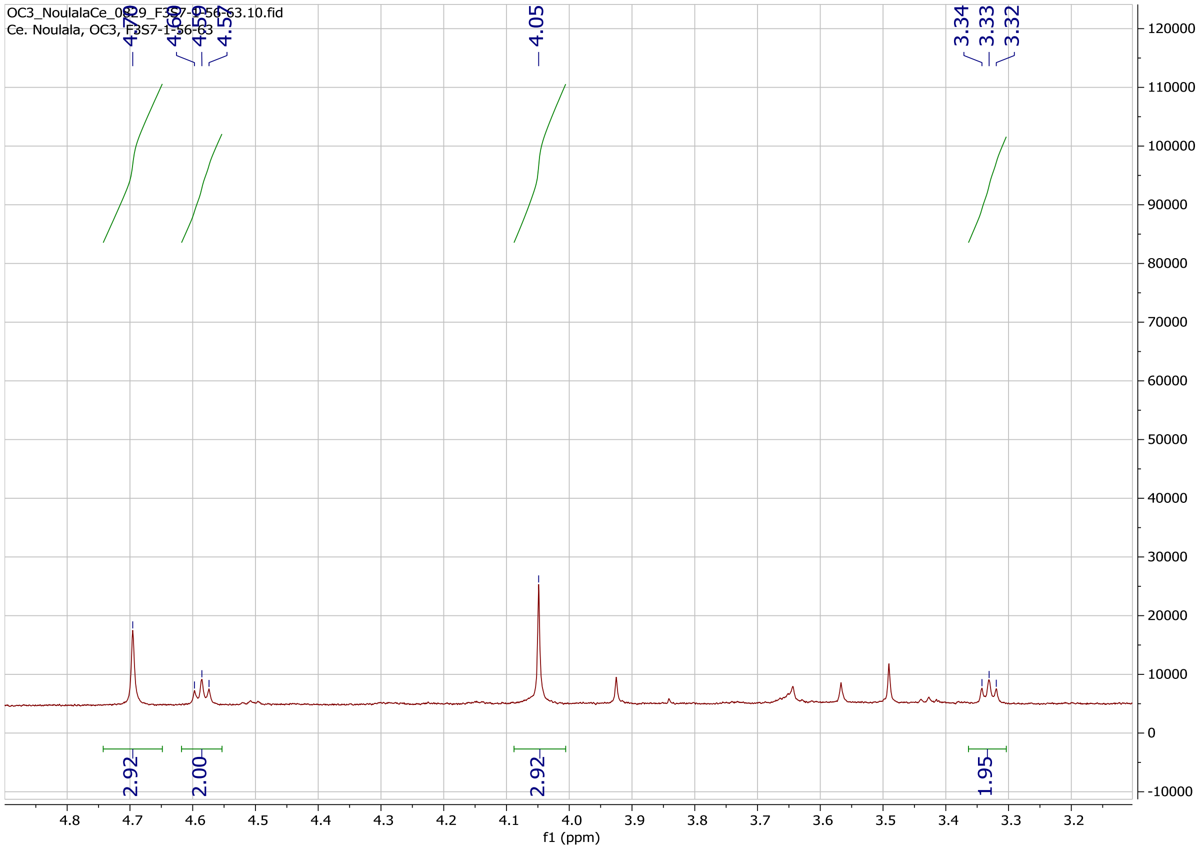

Figure S29b: ^{1}H NMR (600 MHz, $CDCl_3$,) spectra of compound 5 (aliphatic region).

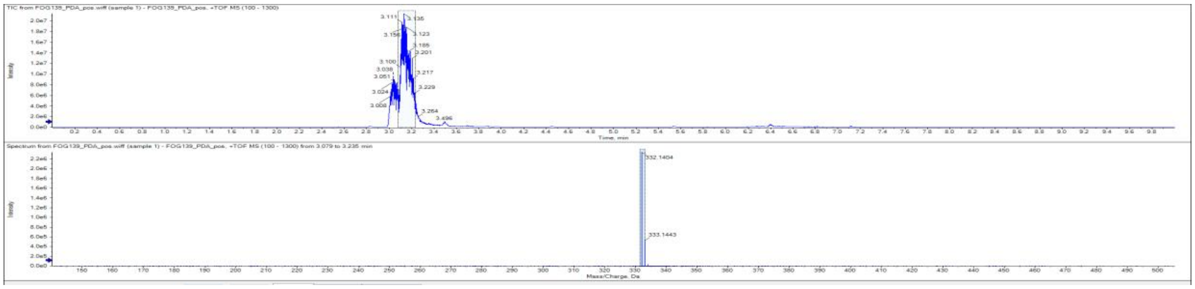

Figure S30 : LC-PDA MS spectrum of soyauxinium chloride

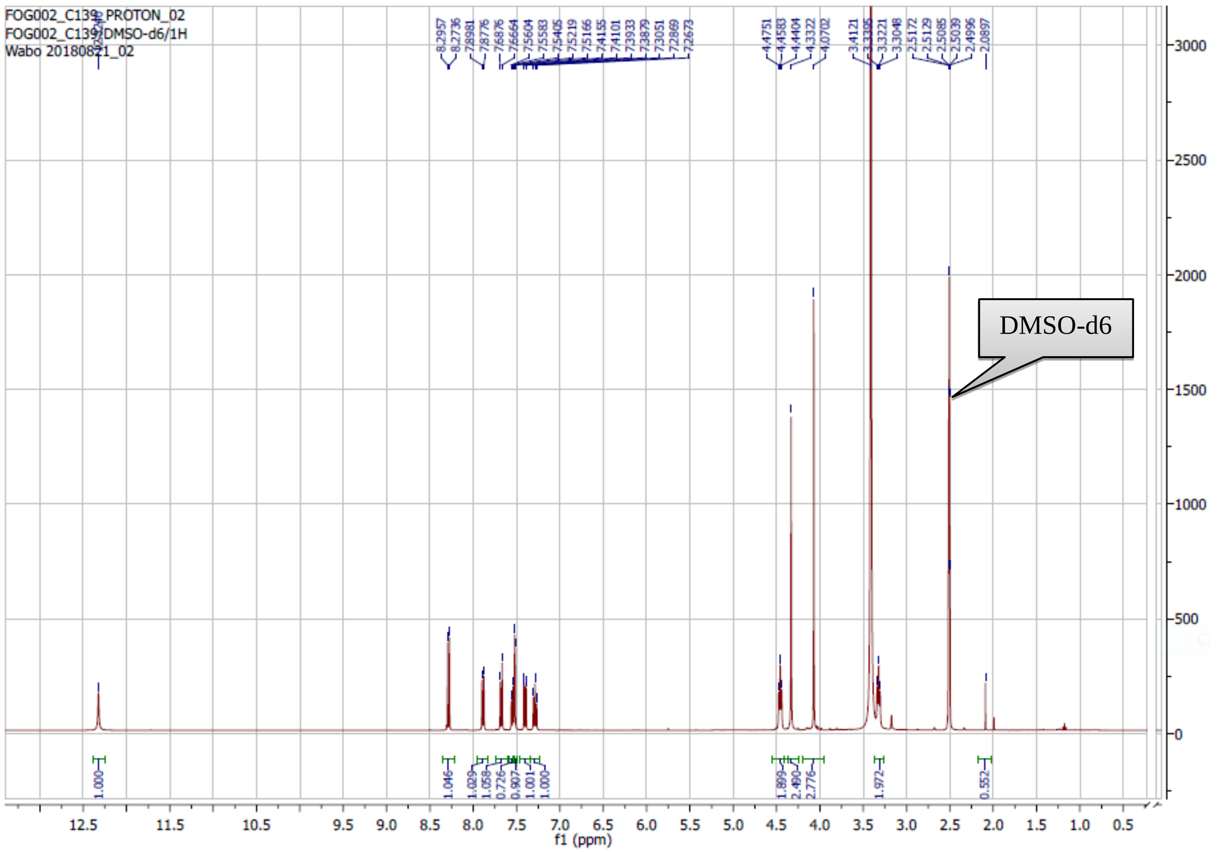
FOG002_C139_PROTON_02
FOG002_C139/DMSO-d6/1H
Wabo 20180821_02
DMSO-d6
f1 (ppm)

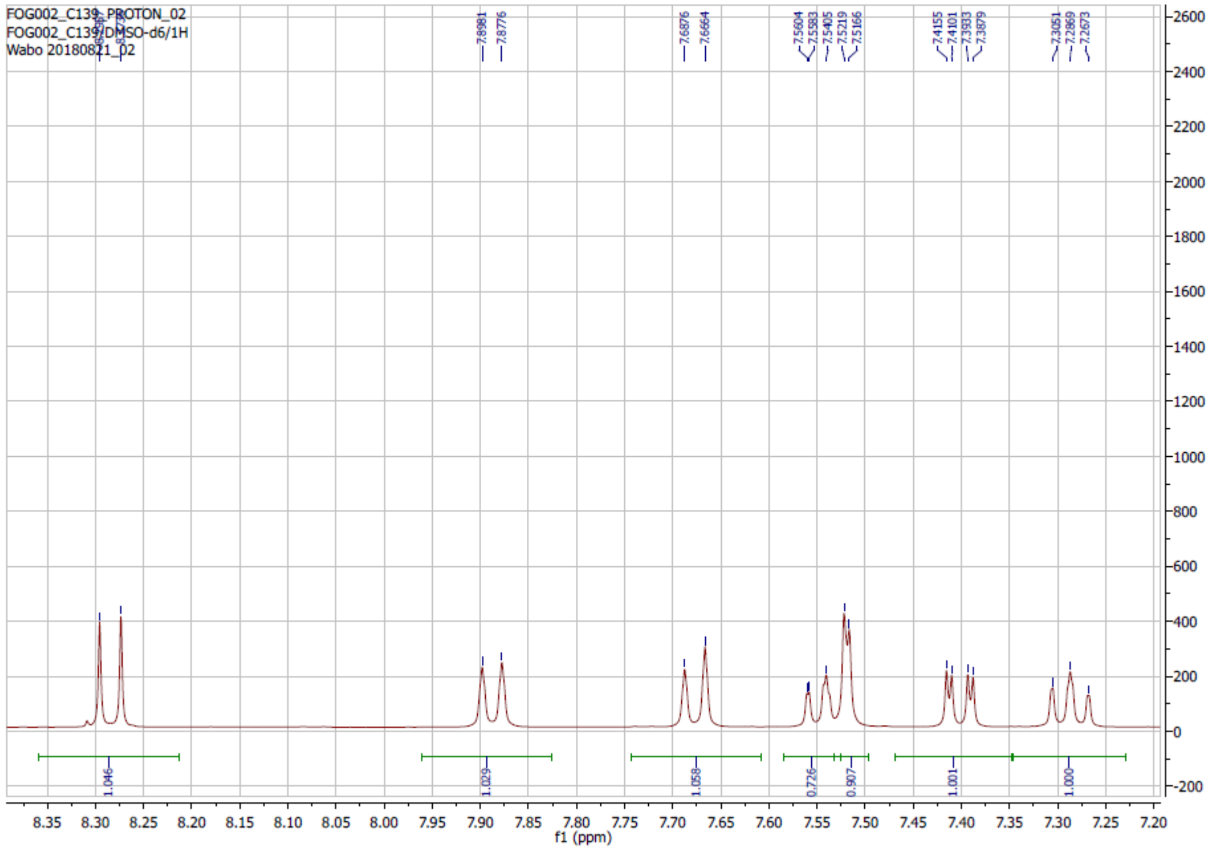
FOG002_C139_PROTON_02
FOG002_C139/DMSO-d6/1H
Wabo 20180821_02
f1 (ppm)

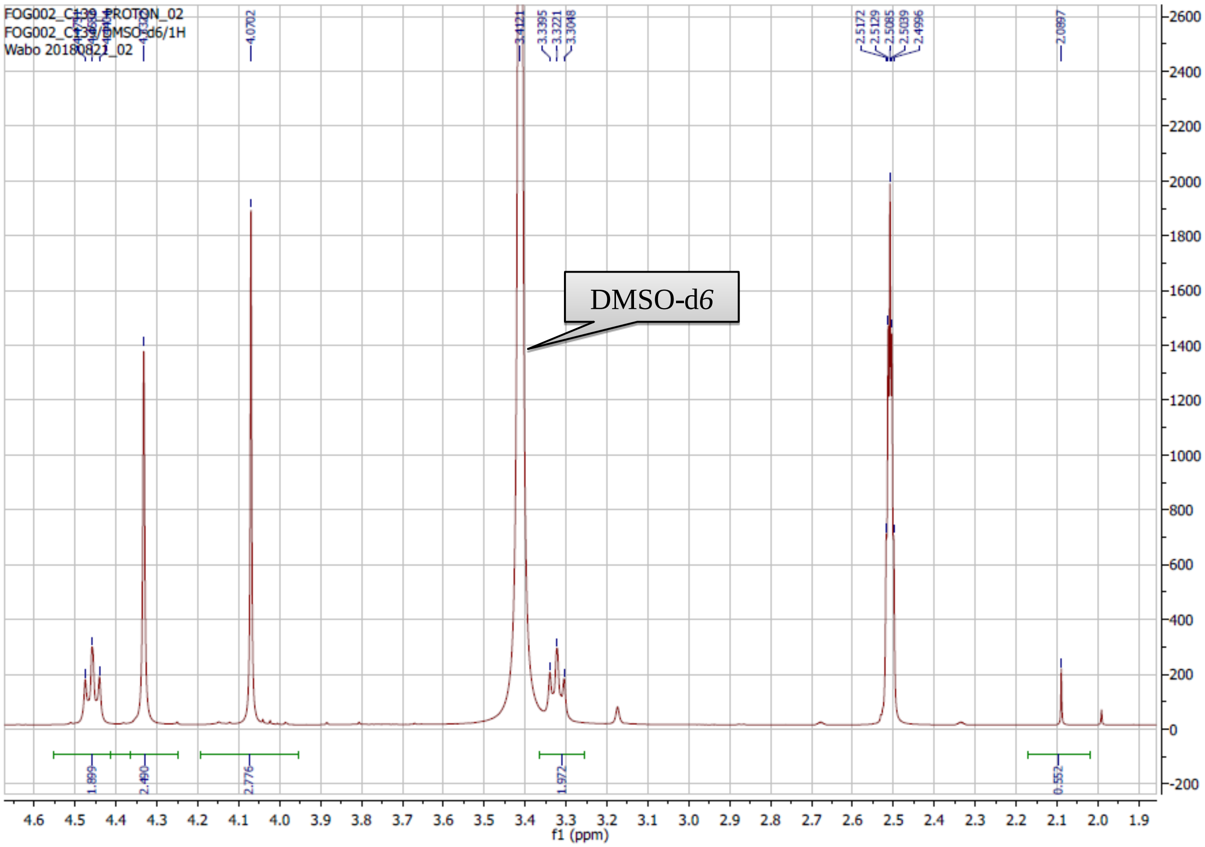

Figure S31 : ^{1}H NMR (DMSO-d6, 400 MHz) spectra of soyauxinium chloride.

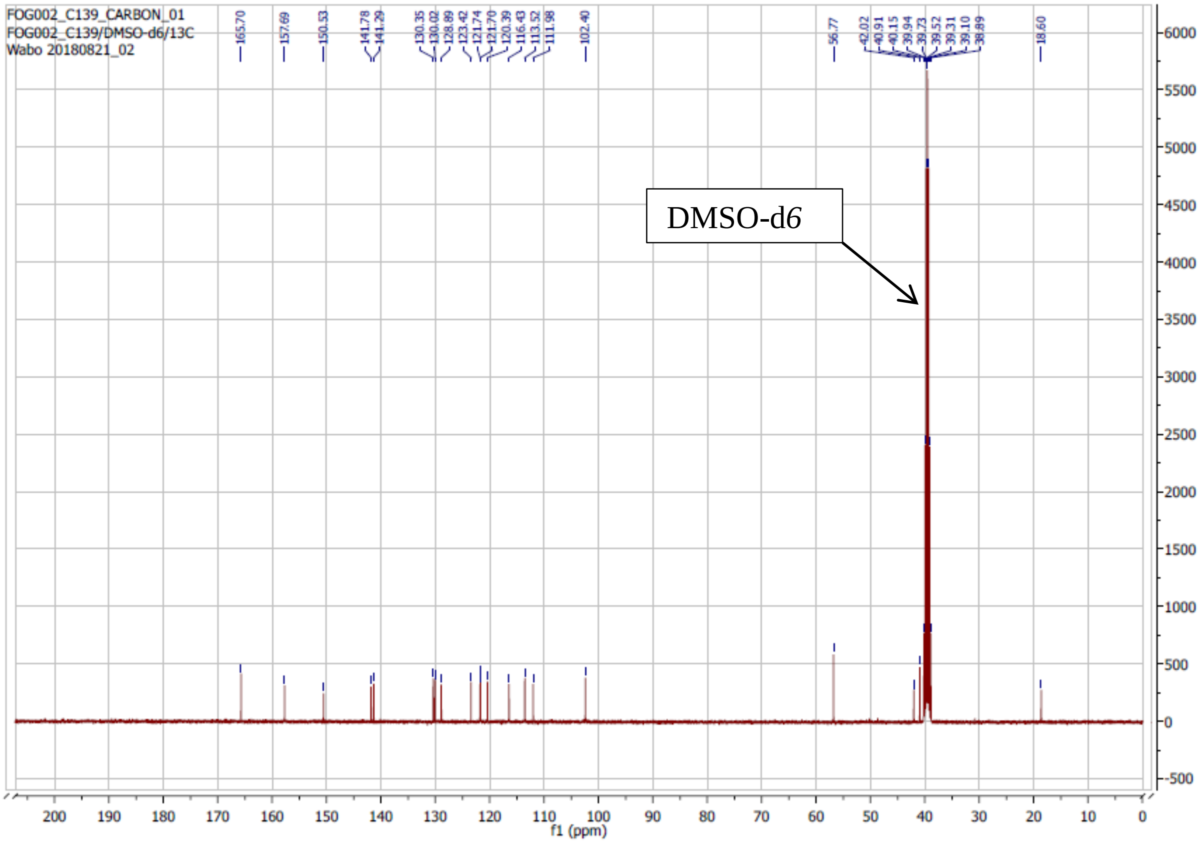

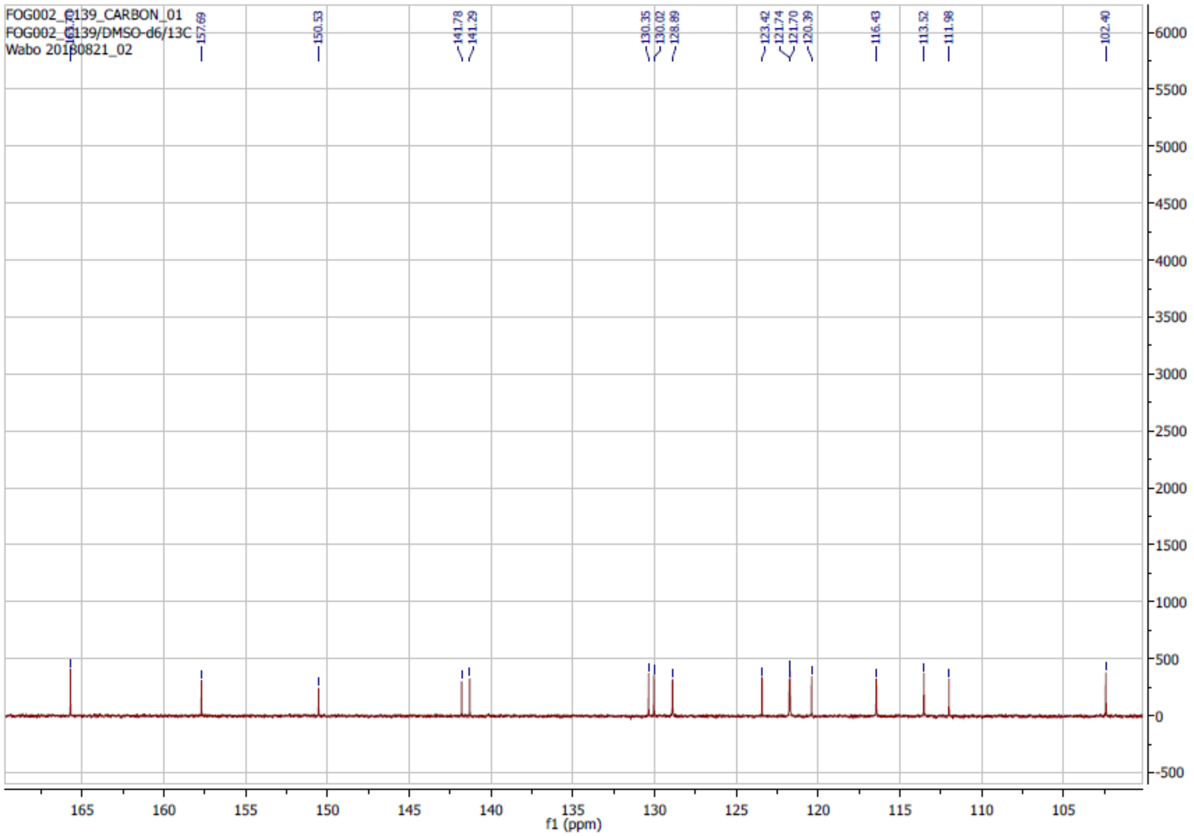

Figure S32 : ^{13}C (DMSO-d6, 100 MHz) spectra of soyauxinium chloride.

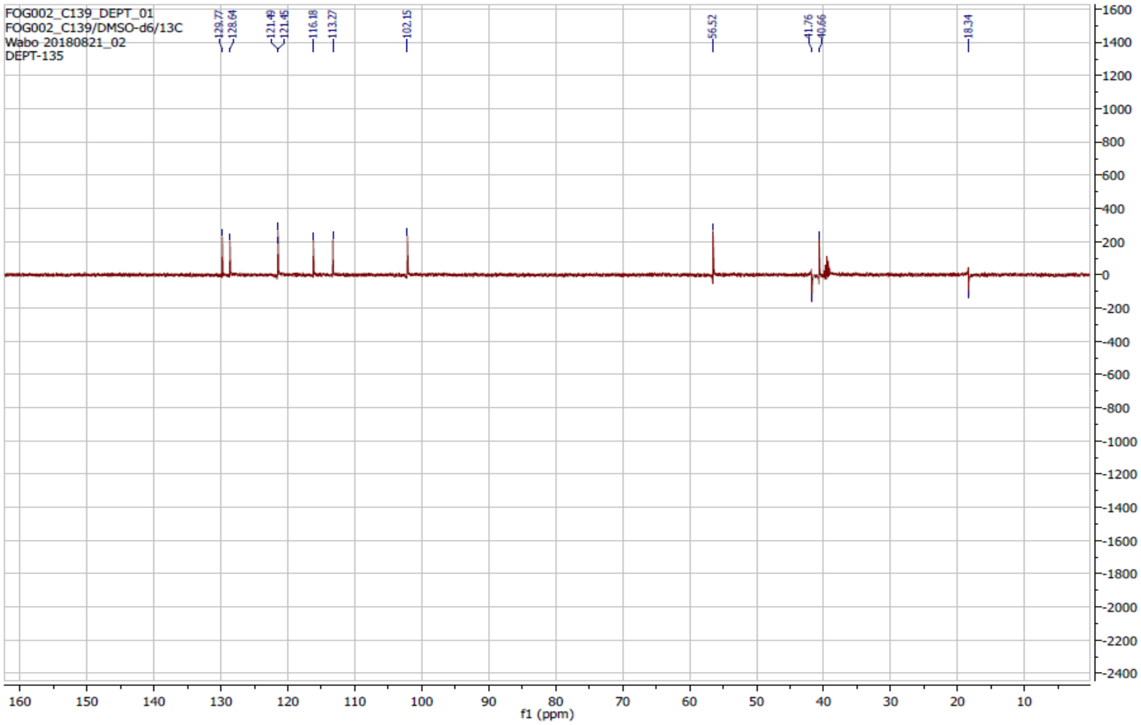

Figure S33 : DEPT 135 spectrum of soyauxinium chloride.

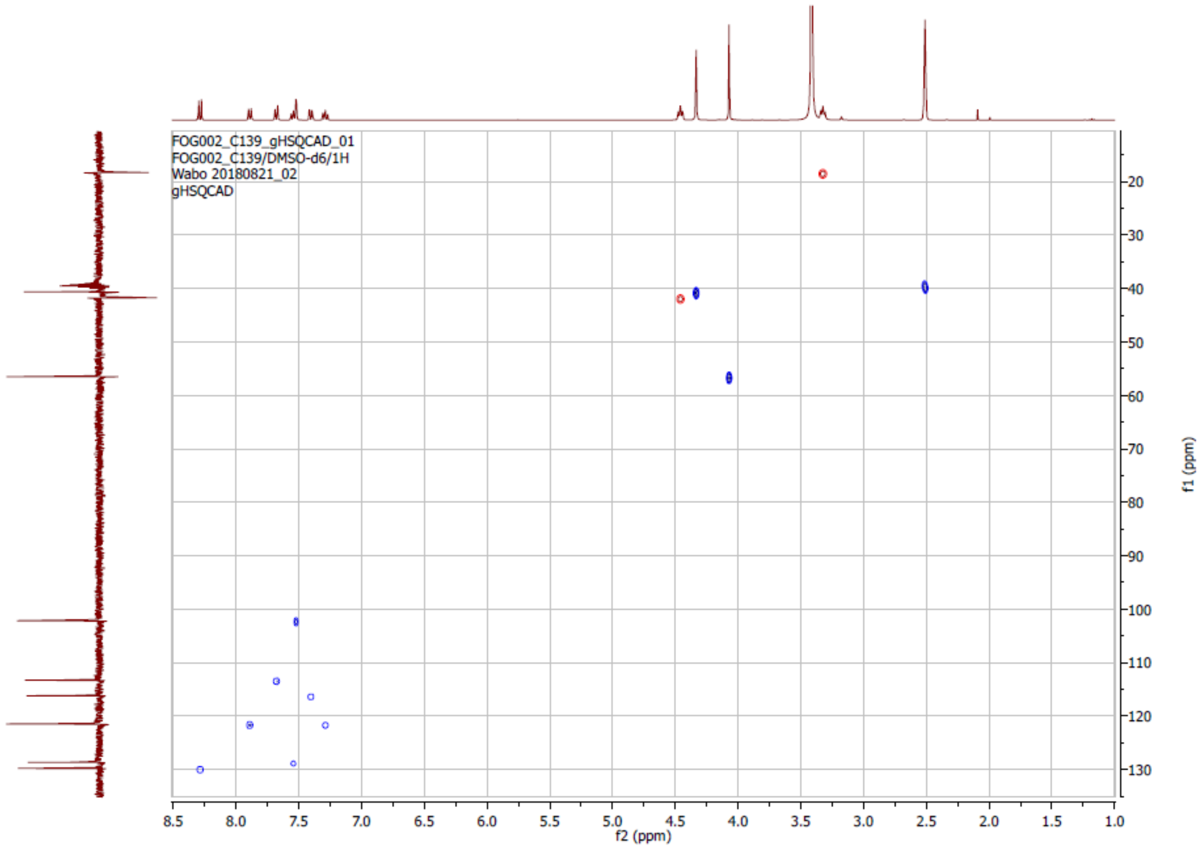

Figure S34 : HSQC spectrum of soyauxinium chloride.

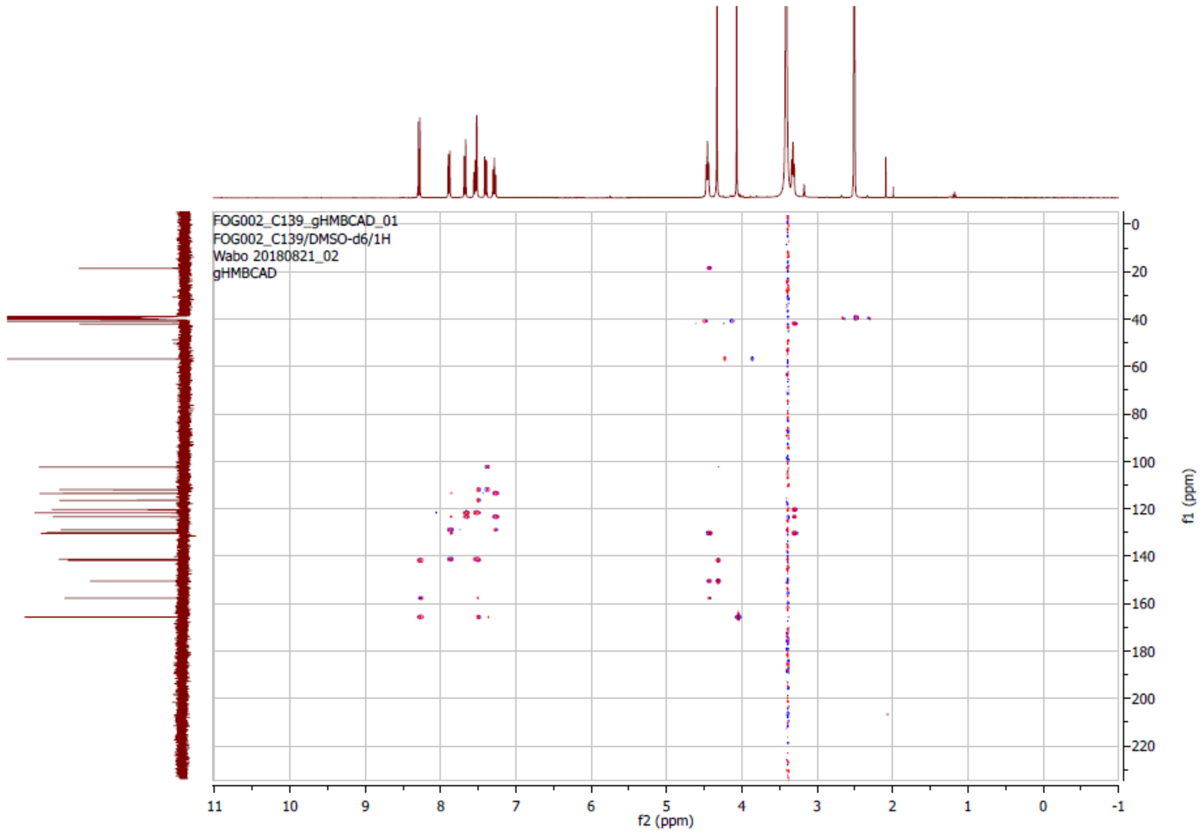

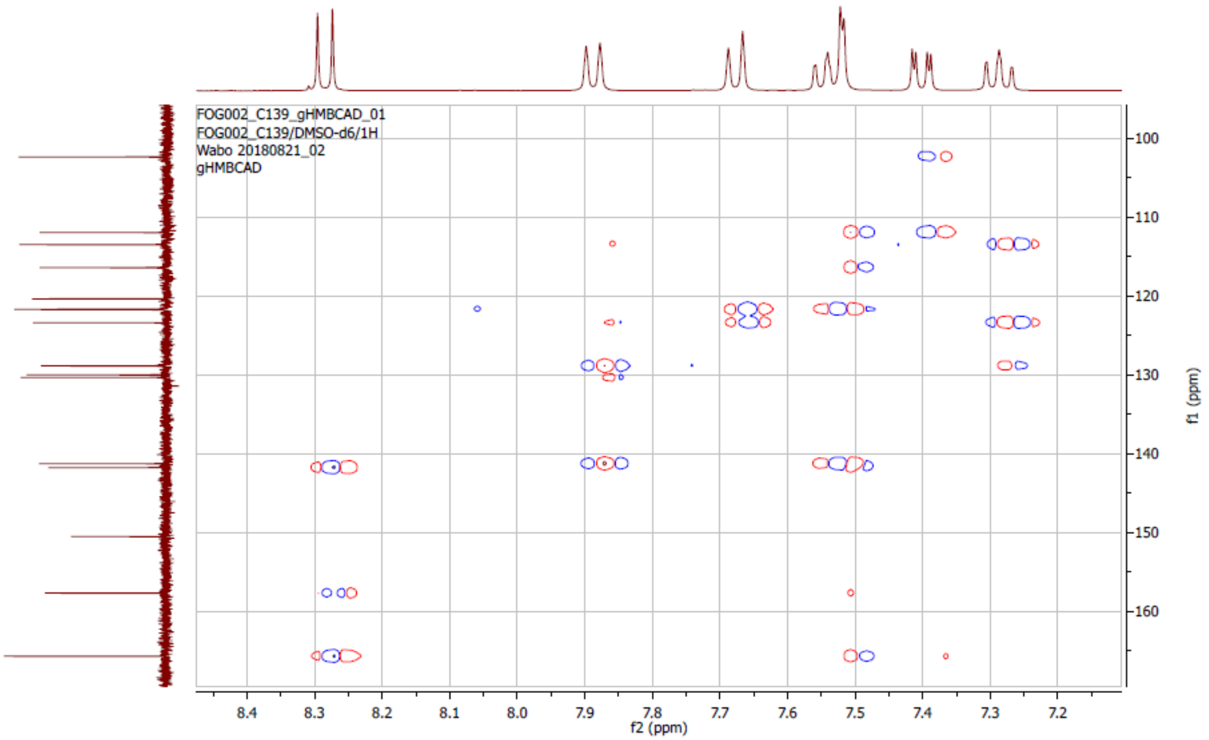

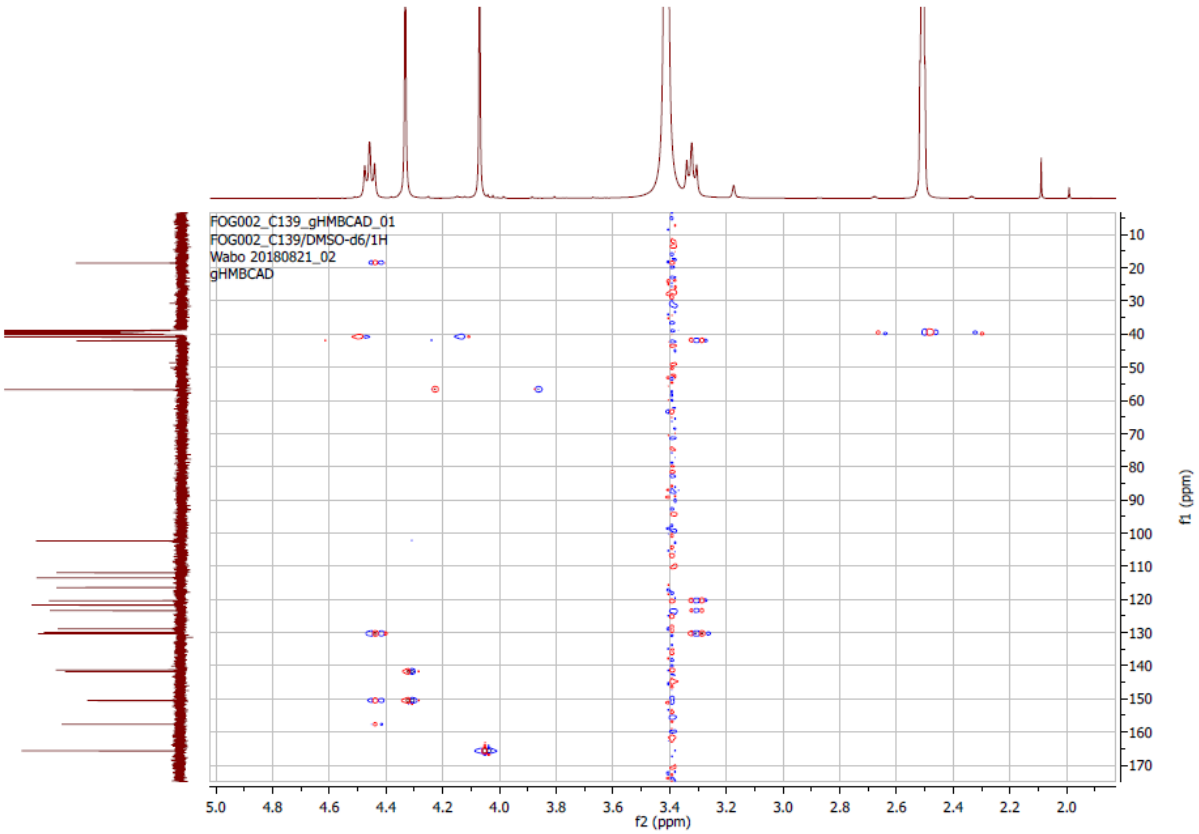

Figure S35 : HMBC spectra of soyauxinium chloride.

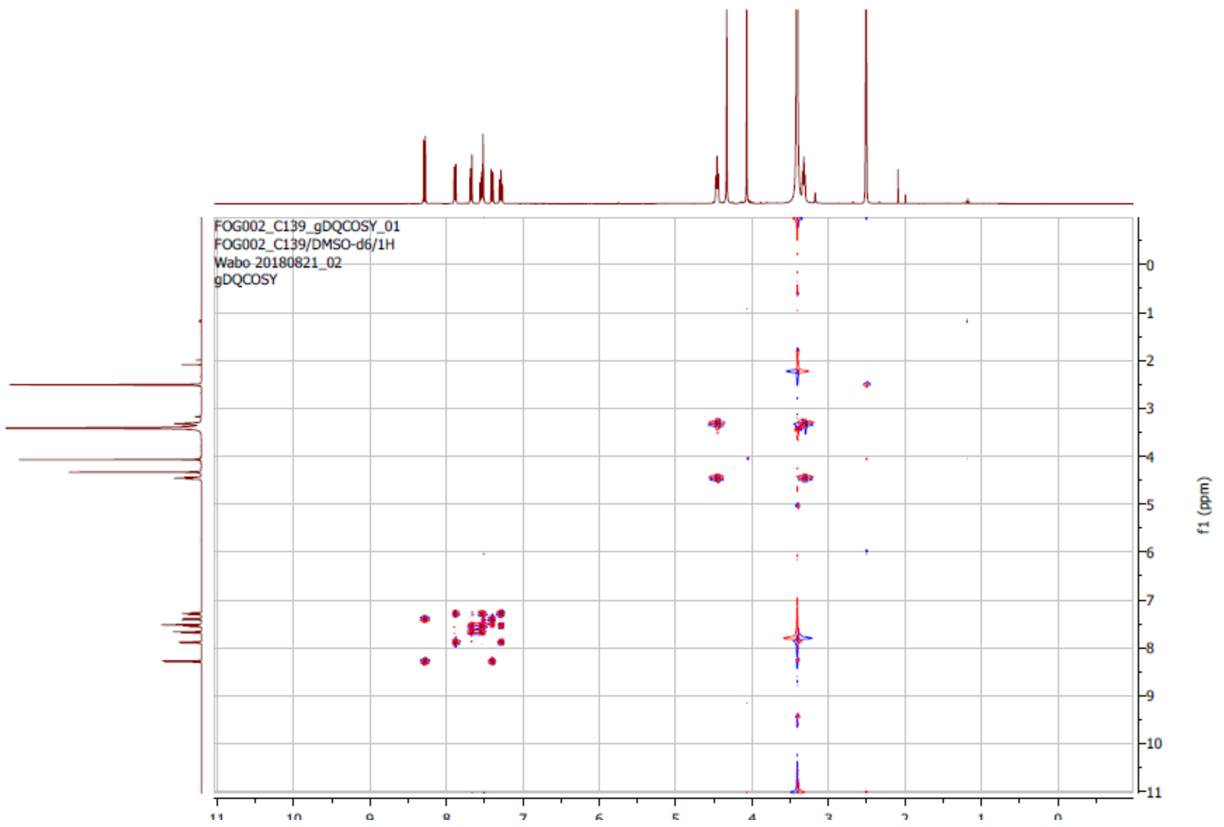
FOG002_C139_gDQCOSY_01
FOG002_C139/DMSO-d6/1H
Wabo 20180821_02
gDQCOSY
f1 (ppm)

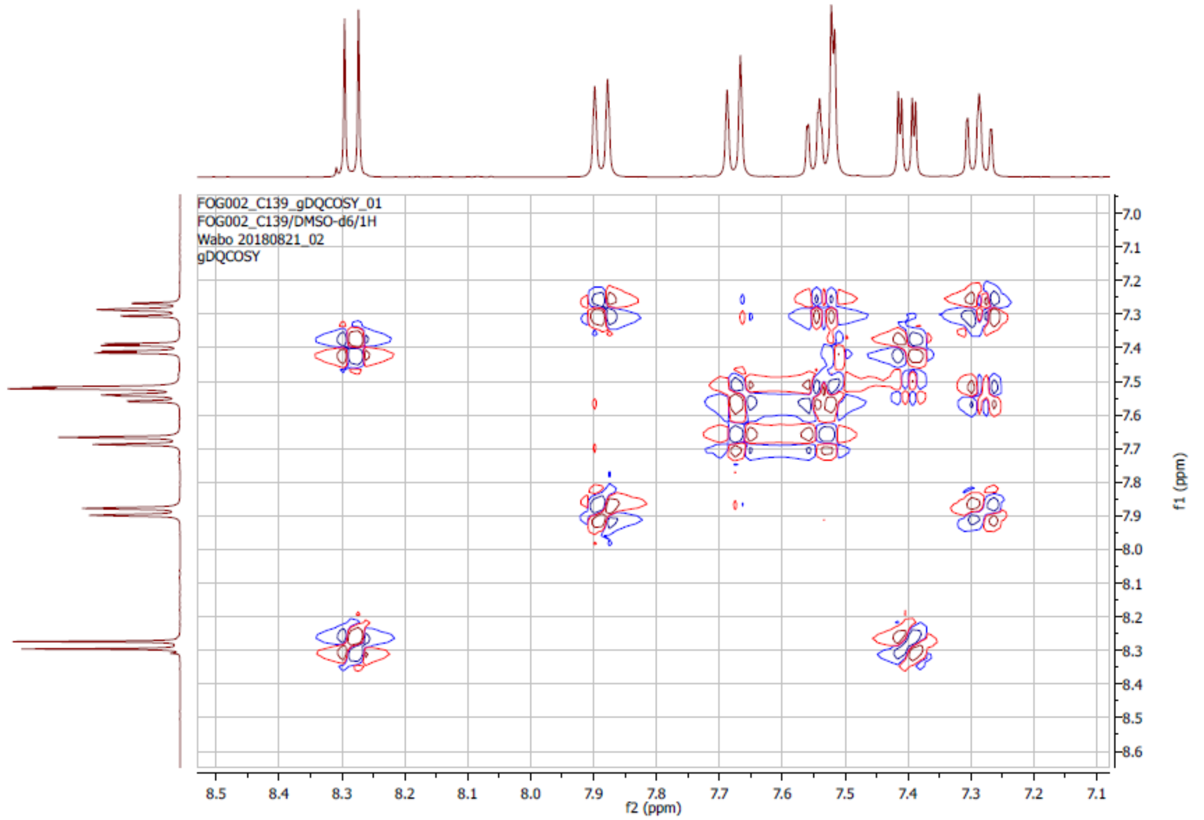
FOG002_C139_gDQCOSY_01
FOG002_C139/DMSO-d6/1H
Wabo 20180821_02
gDQCOSY
f1 (ppm)
f2 (ppm)

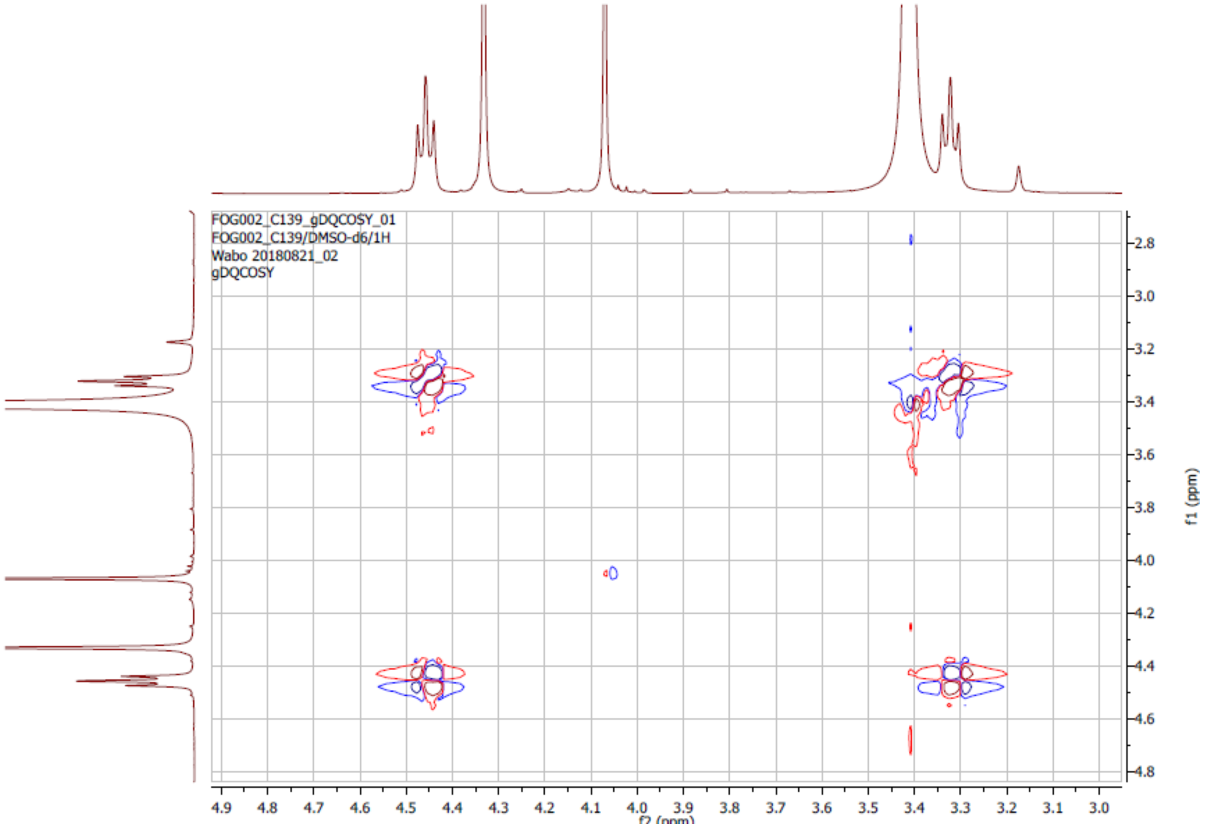

Figure S35 : COSY spectra of soyauxinium chloride
